# Engineered Biocatalyst for Enantioselective Hydrazone Reduction

**DOI:** 10.1002/anie.202424350

**Published:** 2025-04-30

**Authors:** Amy E. Hutton, Fei Zhao, Elizabeth Ho, Jack Domenech, Vanessa Harawa, Murray J. B. Brown, Gideon Grogan, Phillip D. Clayman, Nicholas J. Turner, Anthony P. Green

**Affiliations:** ^1^ Manchester Institute of Biotechnology and Department of Chemistry University of Manchester 131 Princess Street Manchester M1 7DN UK; ^2^ Disyn Biotec Ltd. 33 Matlock Close, Great Sankey Warrington, England WA5 3PZ UK; ^3^ Department of Chemistry University of York Heslington, York YO10 5DD UK; ^4^ GSK, Medicine Development & Supply, GSK GSK Medicines Research Centre Stevenage SG1 2NY UK; ^5^ GSK, Medicine Development & Supply GSK Collegeville PA USA

**Keywords:** Biocatalysis, Directed evolution, Hydrazines, Oxidoreductases, Protein engineering

## Abstract

Enantioselective reduction of hydrazones provides a convergent and versatile route to synthesize hydrazine‐containing motifs that are commonly found in pharmaceuticals and agrochemicals. However, current methods require the use of precious metals, costly chiral ligands, and/or forcing reaction conditions. Here, we report the development of a biocatalytic approach for enantioselective hydrazone reduction using engineered imine reductases. Following evaluation of an in‐house panel of >400 IRED sequences, we identified a single IR361 I127F L179V variant that promotes reduction of Cbz‐protected hydrazones. The introduction of additional two mutations via directed evolution afforded HRED1.1 that is 20‐fold more active than the parent template and promotes reduction of a variety of protected hydrazones in high yields and selectivities (>99% *e.e*.), including in preparative scale biotransformations. Structural analysis of HRED1.1 provides insights into the origins of its unique hydrazone reductase activity. This study offers a powerful biocatalytic route to synthesize valuable chiral hydrazine products and further expands the impressive range of transformations accessible with engineered imine reductases.

## Graphical abstract

Here, we report the discovery and engineering of a hydrazone reductase (HRED) biocatalyst that promotes efficient reduction of protected hydrazones with exceptional stereocontrol. These biotransformations offer a sustainable strategy for synthesizing valuable chiral N─N containing products that are commonly found in pharmaceuticals and agrochemicals.

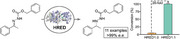

Chiral hydrazines are key structural motifs that are found in many natural products, agrochemicals, and clinically relevant pharmaceuticals (Scheme 1a).^[^ ^1^, ^2^, ^3^, ^4^, ^5^ ^]^ These N─N bond containing compounds can be synthesized from chiral precursors using a variety of methods (Scheme 1b), including alkylation of hydrazines, N─N bond formation using chloramines, and rearrangement of halogenated ureas.^[^ ^6^, ^7^, ^8^, ^9^, ^10^, ^11^ ^]^ Recently, asymmetric hydrogenation of hydrazones has emerged as a valuable alternative that doesn't rely on the use of optically enriched substrates.^[^ ^12^, ^13^, ^14^, ^15^, ^16^, ^17^, ^18^, ^19^, ^20^, ^21^ ^]^ Although powerful, this approach requires the use of transition metal catalysts involving costly chiral ligands along with precious and/or toxic metals. Furthermore, these transformations typically require forcing reaction conditions, including elevated temperatures and high pressures of hydrogen, which hinders their broader application.

**Scheme 1:**
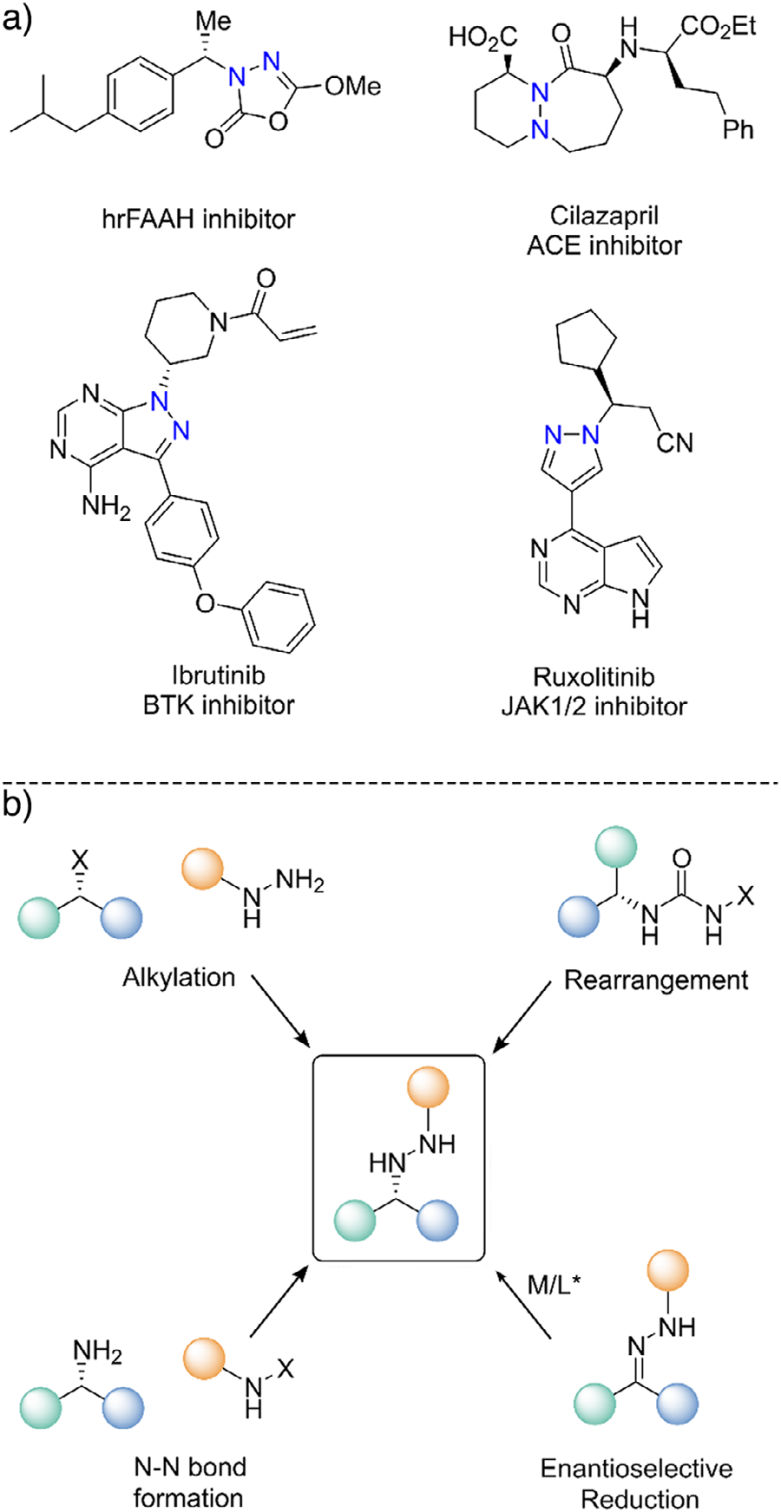
a) Representative bioactive compounds containing chiral hydrazine motifs. b) Synthetic strategies toward chiral hydrazines.

Increasingly, biocatalysis is viewed as an attractive alternative to more traditional synthetic methods due to the high catalytic efficiencies and exacting specificities associated with enzymes. Furthermore, the development of powerful enzyme engineering techniques such as directed evolution allows the properties of biocatalysts to be adapted to meet target applications, including adaption of substrate preferences, improvements in kinetic parameters, and enhanced stability under process conditions.^[^ ^22^, ^23^, ^24^ ^]^ Imine reductases (IREDs) are one such enzyme class that have become widely adopted as biocatalysts for the preparation of chiral amines.^[^ ^25^, ^26^, ^27^, ^28^, ^29^, ^30^, ^31^ ^]^ These nicotinamide adenine dinucleotide phosphate‐dependent enzymes catalyze enantioselective reductions of prochiral imines to a diverse array of amine products, making them attractive targets for industrial biocatalysis. For example, engineered IREDs have been developed for the synthesis of the chiral LSD1 inhibitor GSK2879552^[^ ^32^ ^]^ and for the manufacture of the JAK1 inhibitor acrocitinib.^[^ ^33^ ^]^

Although hydrazones have been generated using an artificially designed enzyme^[^ ^34^ ^]^ and have been shown to be substrates for transaminases,^[^ ^35^ ^]^ reports of enzymatic hydrazone reduction are scarce. To our knowledge there are only two reported cases involving (i) the selective coupling of 3‐cyclopentyl‐3‐oxopropanenitrile with hydrazine hydrate,^[^ ^36^ ^]^ and (ii) a preliminary study using the IRED from *Myxococcus stipitatus*, although no data on conversion or enantioselectivity was provided.^[^ ^37^ ^]^ Given the significant safety concerns associated with the use of hydrazine hydrate as a reagent for large scale synthesis,^[^ ^38^ ^]^ together with a need to identify enzymes capable of reducing C═N bonds in a broader range of functional groups, there remains considerable interest in developing more broadly applicable biocatalytic hydrazone reduction methodologies for applications in asymmetric synthesis. In addition to improved safety, the protected hydrazine products are commonly desired as they facilitate downstream synthetic manipulations. Here, we report the discovery and engineering of a hydrazone reductase (HRED) biocatalyst that furnishes a variety of chiral hydrazine products with high levels of stereocontrol.

We selected biocatalytic reduction of hydrazone **1**, derived from condensation of commercial (benzyloxycarbonyl)hydrazine (Cbz‐hydrazine) and acetophenone, as an initial synthetic target (Figure 1a). To identify a suitable starting template for enzyme engineering, an in‐house panel of >400 IREDs was evaluated for activity toward the target transformation. Biotransformations were performed using cell‐free extracts along with an established NADPH cofactor recycling system using glucose dehydrogenase (GDH). Of the enzymes tested, only one IRED sequence was identified with observable activity toward the conversion of **1** to **1a**, namely, I127F L179V double mutant of IR361 (henceforth named HRED1.0) that was recently engineered by our lab for reductive amination processes involving cyclic secondary amines as substrates.^[^ ^39^ ^]^ Notably, neither the wild‐type IRED361 nor the variants with a single mutation, I127F or L179V, displayed any observable activity under these assay conditions.

**Figure 1:**
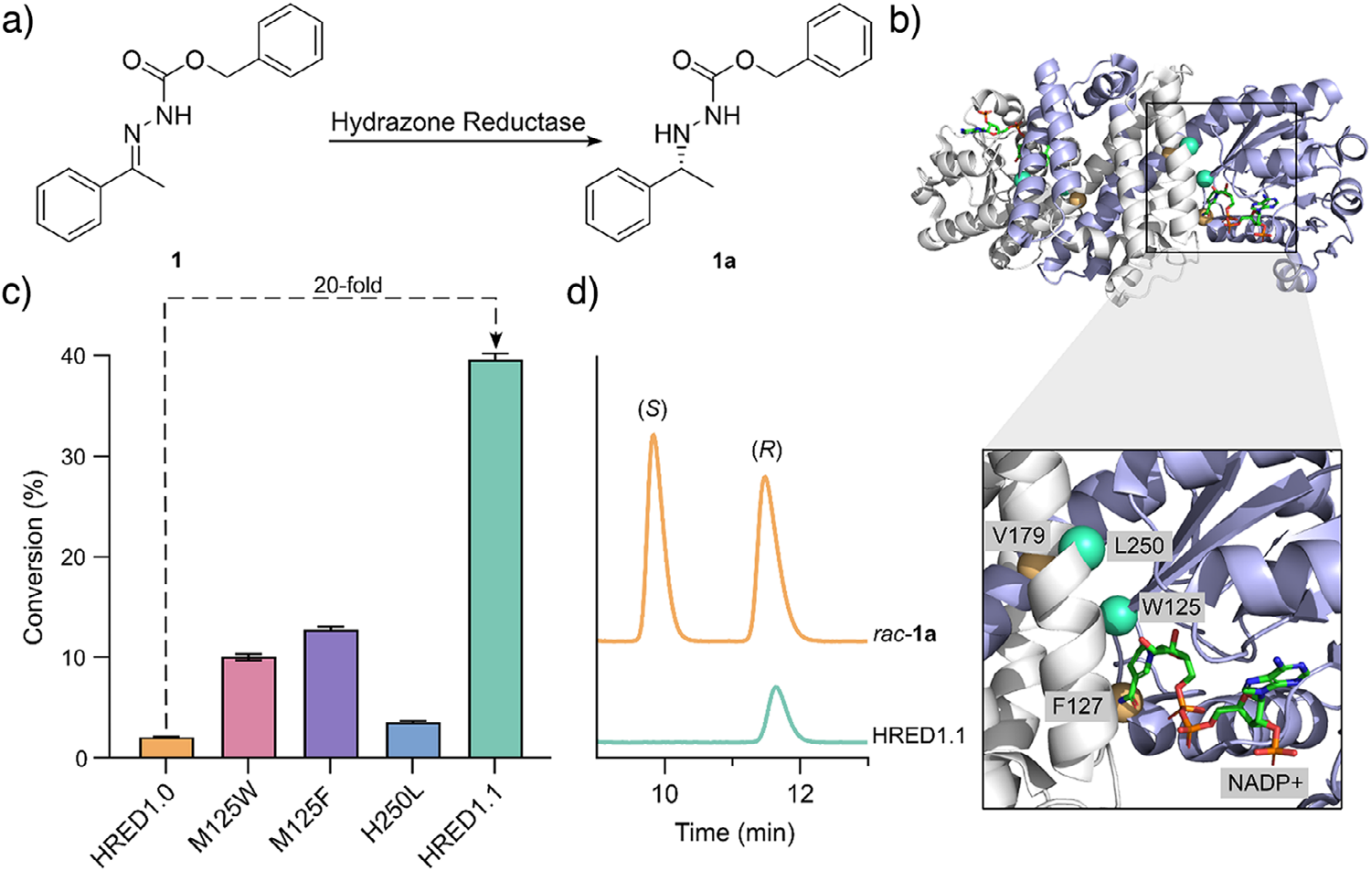
a) Target transformation for the reduction of hydrazone **1** into chiral hydrazine **1a**. b) Structure showing the amino acid positions mutated in HRED1.1. The mutations introduced are represented as spheres at the C_α_ mapped onto the crystal structure of the homodimer IR361 (PDB: 7OSN).^[^ ^40^ ^]^ I127F and L179V mutated in IR361 to afford HRED1.0 are in orange, and M125W and H250L to afford HRED1.1 are in green. The NADP^+^ cofactor is shown as atom‐colored sticks with green carbons. c) Bar chart showing the improvement in activity achieved with selected point mutations to HRED1.0 and the combination of two mutations to afford HRED1.1. Biotransformations were performed using **1** (5 mM) and enzyme (25 µM) in 100 mM KPi pH 7.0 with NADP^+^ (500 µM), GDH (1 mg mL^−1^), D‐glucose (50 mM), and 5% (v/v) DMSO as a cosolvent. Reactions were analyzed following 25 h incubation at 30 °C. Error bars represent the standard deviation of measurements made in triplicate. d) Chiral HPLC trace of *rac*‐**1a** and the product of a biotransformation of HRED1.1 under the same conditions as Figure 1c.

To enhance hydrazone reduction activity, HRED1.0 was subjected to a single round of evolutionary optimization. We targeted 22 positions within the active site, including those in close proximity to the NADPH cofactor and in the putative substrate‐binding pocket, which were individually randomized using NNK degenerate codons. Individual library variants were arrayed in 96‐well plates and evaluated as clarified cell lysate using a UPLC assay monitoring the conversion of **1** to **1a**. Analysis of >2000 variants led to the identification of four point mutations (M125F, M125W, H250L, A100T) displaying increased activity. Substitution of Met125 to either Phe or Trp gave substantial 6‐fold and 5‐fold increases in reaction conversion, respectively, under assay conditions used for evolution (Figure 1c and Table S1), with more modest ca. 1.5‐fold improvements observed with the H250L and A100T mutations (Figure S1 and Table S1). These beneficial mutations were subsequently combined by DNA shuffling, leading to the identification of a M125W H250L double mutant (HRED1.1) that displays a significant 20‐fold improvement in reaction conversion, and a 19‐fold improvement in reaction rate, over the parent HRED1.0 template (Figures 1b,c and S2; Table S1).

Using HRED1.1 as a biocatalyst, complete conversion of **1** (5 mM) to **1a** can be achieved within 18 h (Figure 2 and Table S1). For comparison, under identical assay conditions, only 5% conversion is observed with HRED1.0. Furthermore, HRED1.1 displays exceptional levels of stereocontrol, affording (*R*)‐enantiomer of **1a** in >99% *e.e*. (Figure 1d). The synthetic utility of HRED1.1 was demonstrated in a preparative scale biotransformation to yield 123 mg of (*R*)‐**1a** (98% conversion, 92% isolated yield) (Table S1). Optimization of reaction conditions (Figures S3–S9) afforded a 10‐fold reduction in NADP^+^ and GDH‐901 concentrations compared to those used during evolution without significant reduction in conversion. The high yields achieved by HRED1.1 are particularly notable given that the biotransformations are heterogeneous due to the low solubility of both hydrazone **1** and hydrazine **1a**. Beyond reduction of preformed hydrazones, HRED1.1 can also achieve reductive coupling of acetophenone and Cbz‐hydrazine with modest conversions and exceptional stereocontrol (Figure 2).

**Figure 2:**
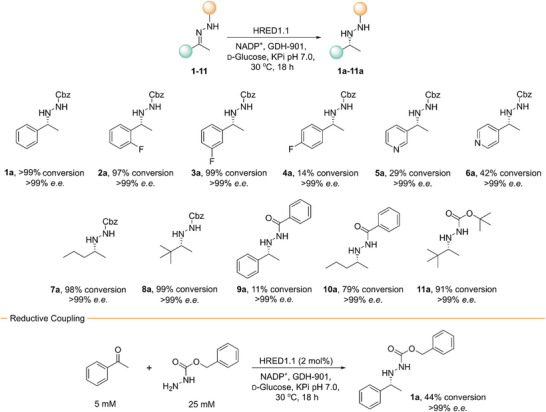
HRED1.1 promotes the reduction of a range of substrates with high conversions and excellent selectivity. Biotransformations were performed using substrate (5 mM) and HRED1.1 (2–5 mol%) in 100 mM KPi pH 7.0 with NADP^+^ (500 µM), GDH (1 mg mL^−1^), D‐glucose (50 mM), and 5% (v/v) DMSO as a cosolvent. Reactions were analyzed following 18 h incubation at 30 °C. The absolute stereochemistry of **1a** was assigned by comparison to optical rotation values reported in the literature.^[^ ^7^ ^]^ The stereochemistry of **2a–8a** and **10a**–**11a** was assigned by analogy to HRED1.1‐derived (*R*)‐**1a**. The stereochemistry of **9a** was assigned by comparison of HPLC retention times to those reported in Ref. [41]. HRED1.1 (2 mol%) promotes the reductive coupling between acetophenone (5 mM) and benzyloxycarbonyl)hydrazine (25 mM) with modest conversion and exceptional stereocontrol.

To further explore its synthetic utility, HRED1.1 was evaluated for activity toward a range of protected hydrazones **1**–**11** (Figure 2 and Table S2). In all cases, HRED1.1 achieves exceptional levels of stereocontrol (>99% *e.e*.). Derivatives of **1** containing *ortho*‐, *meta*‐, or *para*‐fluoro substituents (**2**–**4**) are all well tolerated, albeit with reduced conversions for the *para*‐derivative **4**. Analogues **5** and **6** containing pharmaceutically relevant pyridyl motifs are also substrates for HRED1.1.^[^ ^42^, ^43^ ^]^ Substrates containing larger aryl‐substituents are not accommodated by HRED1.1 due to steric constraints within the active site pocket (Figure S10). Notably, the scope of HRED1.1 is not limited to aromatic hydrazones; substrates **7** and **8**, derived from the aliphatic ketones pentanone and *tert*‐butyl methyl ketone, respectively, were efficiently converted in high yields. Finally, we explored the activity of HRED1.1 with a selection of substrates derived from commercial hydrazine reagents featuring orthogonal benzoyl (**9**–**10**) and Boc (**11**) protecting groups. Importantly, these modifications are also well tolerated by HRED1.1, thus further expanding the synthetic utility of our hydrazone reductase biocatalyst.

The improved ability of HRED1.1 to accept the hydrazone **1** was investigated using X‐ray crystallography and modeling. The structure was determined to a resolution of 2.77 Å, with two molecules, A and B, in the asymmetric unit comprising one dimer. The structure was very similar overall to that of wild‐type IR361 (PDB 7OSN),^[^ ^40^ ^]^ displaying the canonical IRED fold, in which an N‐terminal Rossman fold domain is attached to a C‐terminal helical bundle through a long interdomain helix. Two active sites are formed at the interfaces of the N‐terminal domain of one monomer and the C‐terminal bundle of the other through reciprocal domain sharing. The crystals contained a large proportion of water (71%) and the modest resolution was accompanied by poor electron density in the N‐terminal domain of monomer B, leading to some zero‐occupancy residues and many side‐chains not being modeled in that region. However, the density within monomer A and the C‐terminal bundle of B was good, permitting the building of side chains and NADP^+^ molecule within the active site at the interface of the domains. Omit density for the more structurally consequential mutations (M125W and I127F) was also clear (Figure S11). Superimposition of the HRED1.1 structure with that of wild‐type IR361 revealed the consequences of the mutations (Figure 3a). The substitution of I for F in position 127 at the bottom of the active site as presented in Figure 3a has, counterintuitively, made more space in the active site through the displacement of the side chain of (B)Y221. Mutation of M125 to W at the top of the active site has again created more space, despite being a larger residue as it projects upward and makes a new stabilizing interaction with the side chain of D176 through the N‐atom on the indole ring. Further stabilization of this conformation may be provided by a stacking interaction between the indole and the mutated 179 position from L to V, which lies on the interdomain helix on the other side of the active site cavity. (B)H250L is situated at the interface between the N‐terminal domain of one monomer and the C‐terminal bundle of its neighbor and may provide stabilizing hydrophobic interactions in a mode that has been observed for other IREDs in engineering studies.^[^ ^29^, ^32^, ^33^, ^44^ ^]^

**Figure 3:**
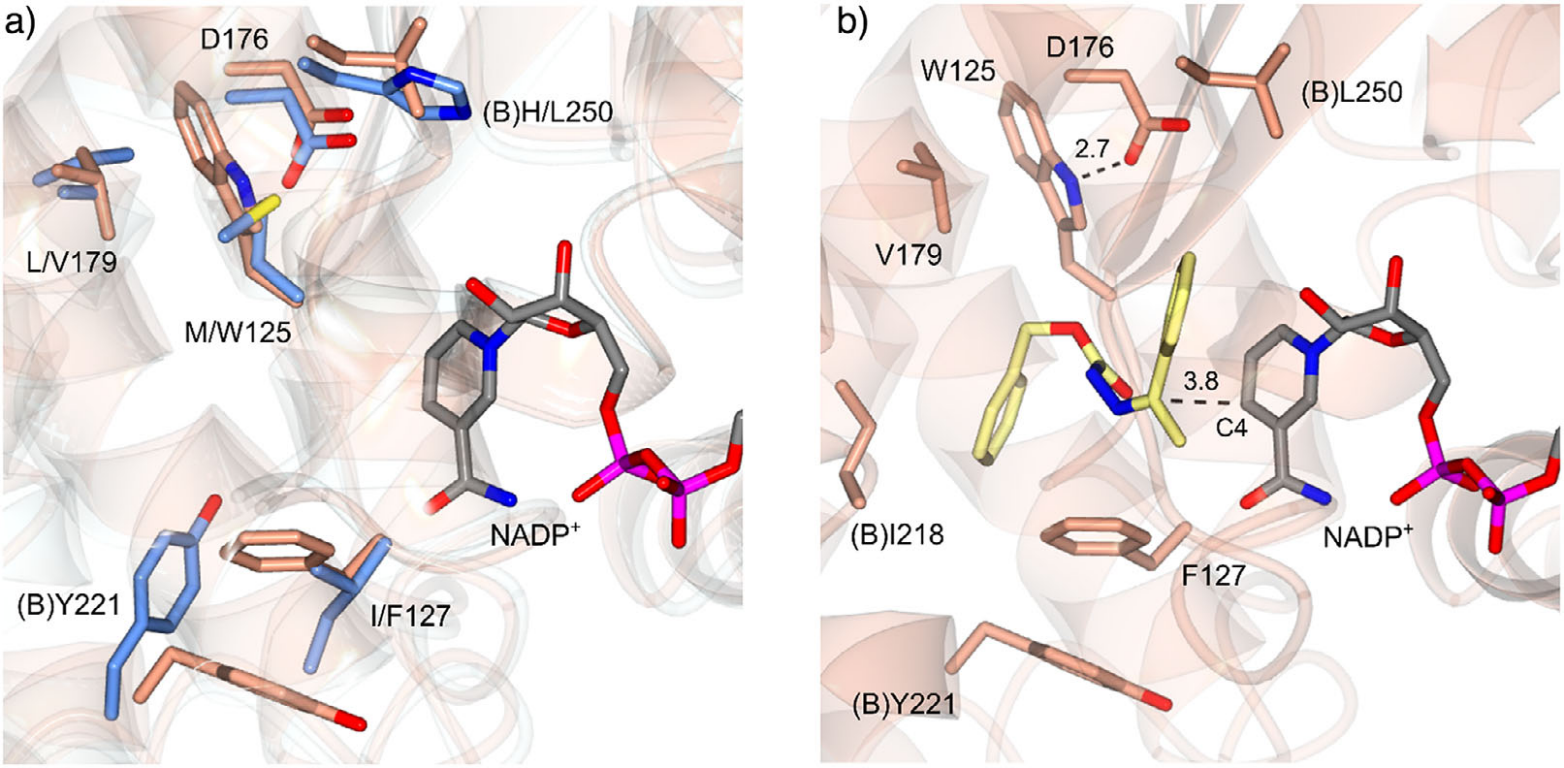
Crystal structure and active site residues of HRED1.1. a). Superimposition of active sites of wild‐type IR361 (PDB 7OSN)^[^ ^40^ ^]^ with HRED1.1. Carbon atoms are in blue and pink, respectively. b). Active site of HRED1.1 modeled with hydrazone **1** (carbon atoms in yellow). Selected interaction is shown as a black dashed line with the distance in Å.

The structure of HRED1.1 was modeled with the substrate **1** (Figure 3b) using Autodock VINA.^[^ ^45^ ^]^ One of the lowest energy conformations positioned the ligand with the electrophilic carbon of C═N bond at a suitable approximate distance (∼3.8 Å) from the C4 of the nicotinamide ring of NADP^+^ to receive a hydride, and on the correct *si* prochiral face to furnish the experimentally observed (*R*)‐enantiomer of **1a**. The Cbz‐group was observed to project backward into the active site into the space made accessible by the M125W and I127F mutations described above. The F127 residue may also participate in productive π–π interactions with the Cbz aromatic ring. It appears, therefore, that the improved activity of the mutant toward **1** may derive from better accommodation of the Cbz group.

In summary, we have established a biocatalytic platform for selective reduction of hydrazones to afford valuable chiral hydrazine motifs that are common precursors for pharmaceutical and agrochemical synthesis. Our analysis of >400 IRED sequences suggests that hydrazone reduction is a rare activity within the imine reductase family. Nevertheless, introduction of only a handful of mutations into IR361 has led to the development of an engineered HRED biocatalyst that achieves exceptional selectivities across a range of hydrazone substrates, with high reaction yields also achievable with elevated enzyme loadings (2 mol%). Moving forward, more extensive engineering will undoubtedly deliver more potent HRED biocatalysts, including those that are tailored toward specific target products and/or adapted to operate under viable process conditions. In contrast to existing methods of hydrazone reduction, our biocatalytic approach is not reliant on precious/toxic metals, costly ligands, or forcing reaction conditions, and therefore offers a more sustainable strategy for synthesizing valuable chiral N─N containing products.

## Supporting Information

The authors have cited additional references within the Supporting Information.^[^ ^46^, ^47^, ^48^, ^49^, ^50^, ^51^, ^52^, ^53^ ^]^

## Conflict of Interests

The authors declare no conflict of interest.

## Supporting information



Supporting Information

## Data Availability

The data that support the findings of this study are available from the corresponding author upon reasonable request.
