# Supplementary material for: Engineered Biocatalyst for Enantioselective Hydrazone Reduction
Source: Angew Chem Int Ed Engl. 2025 Apr 30;64(26):e202424350. doi: 10.1002/anie.202424350 (PMC12184316; doi:10.1002/anie.202424350)
Supplement: Supplementary file 1 — Supporting Information [file ANIE-64-e202424350-s001.pdf]

Supplementary Information

An Engineered Biocatalyst for Enantioselective Hydrazone Reduction

Amy E. Hutton[a,b], Fei Zhao[a], Elizabeth Ho[c], Jack Domenech[c], Vanessa Harawa[d], Murray J. B. Brown[d], Gideon Grogan[c]*, Phillip D. Clayman[e]*, Nicholas J. Turner[a]* and Anthony P. Green[a]*

[a] Manchester Institute of Biotechnology and Department of Chemistry, University of Manchester, 131 Princess Street, Manchester, M1 7DN, UK

[b] Disyn Biotec, 33 Matlock Close, Great Sankey, Warrington, England, WA5 3PZ, UK.

[c] Department of Chemistry, University of York, Heslington, York, YO10 5DD, UK

[d] GSK, Medicine Development & Supply, GSK Medicines Research Centre, Stevenage, SG1 2NY, UK

[e] GSK, Medicine Development & Supply, Collegeville, PA, USA

Contents

Experimental Procedures 2

Supplementary Figures 15

Supplementary Tables 22

DNA and Protein Sequences 27

NMR Spectra 28

Chiral Chromatograms 54

References 65

Experimental Procedures

Materials

All chemicals and biological materials were obtained from commercial suppliers. Substrates from Sigma Aldrich, Thermo Scientific or Fluorochem; $NADP^+$ from Prozomix; D-glucose dehydrogenase (GDH) (CDX-901) from Codexis; Lysozyme, DNase I, IPTG, Terrific Broth media and kanamycin were purchased from Sigma Aldrich; polymyxin B sulfate from AlfaAesar; LB agar and LB media Formedium; *Escherichia coli* 5 alpha, *Escherichia coli* BL21(DE3), Q5 DNA polymerase, T4 DNA ligase and endonucleases from New England BioLabs; and oligonucleotides were synthesized by Integrated DNA Technologies.

NMR spectroscopy was recorded using a Bruker Advance, *J* values are given in Hz and reported chemical shifts (δ, ppm) are relative to the deuterated solvent residual protic signal. High-resolution mass spectrometry (HRMS) was recorded using a Waters LCT time-of-flight mass spectrometer, connected to a Waters Alliance LC (Waters, Milford, MA, USA).

Screening of IRED panel

The panel of >400 IRED sequences were screened in cell-free lysate using either lyophilised lysate or freshly lysed cell pellets. Cell pellets were obtained from the expression of the IRED sequences in 96-well plate format as 500 µL cultures and lysed a using the same method as described for library screening below. For the biotransformations, 5 µL of **1** (5 mM final concentration) in DMSO (5% v/v final), 20 µL of KPi buffer pH 7.0 and 25 µL turnover mix (500 µM $NADP^+$, 1 mg/mL GDH and 50 mM D-glucose, final concentration) were added to a 96-well polypropylene microtiter plate. The reactions were initiated by the addition of 50 µL of either clarified lysate or resuspended lyophilised lysate (10 mg/mL). Assay plates were sealed and incubated for 20 h at 30 °C, 80% humidity with shaking 850 r.p.m. Reactions were quenched with addition of 100 µL MeCN, heat sealed and incubated for 2 h at 850 r.p.m. at 30 °C. Precipitated protein was removed by centrifugation at 2,900*g* for 10 min. 100 µL of the clarified reactions were transferred to 96-well polypropylene microtiter plates and heat sealed with pierceable foil for UPLC analysis as described below.

Protein production and purification

For expression of HRED1.0 (IR361 I127F L179V)[1], HRED1.1 and variants, chemically competent *E. coli* BL21 (DE3) cells were transformed with the relevant pET29b(+)_HRED constructs. Single colonies of freshly transformed cells were cultured for 18 h in 5 mL LB medium containing 50 µg/mL kanamycin. Starter cultures (500 µL) were used to inoculate 50 mL Terrific Broth (TB) medium supplemented with 50 µg/mL kanamycin. Cultures were grown at 37 °C, 180 r.p.m. to an optical density at 600 nm (OD_{600}) of around 0.6. Protein expression was induced with the addition of IPTG to a final concentration of 0.1mM. Induced cultures were incubated for 20 h at 25 °C and the cells were subsequently collected by centrifugation (3,220*g* for 10 min). Pelleted cells were resuspended in lysis buffer (100 mM KPi, 300 mM NaCl, pH 7.5 containing 20 mM imidazole) and lysed by sonication. Cell lysates were cleared by centrifugation (27,216*g* for 30 min) and supernatants were subjected to affinity chromatography using Ni-NTA Agarose (Qiagen). Purified protein was eluted using 100 mM KPi, 300 mM NaCl, pH 7.5 containing 250 mM imidazole. Proteins were desalted using 10DG desalting columns (Bio-Rad) with 100 mM KPi pH 7.0 and

analyzed by SDS PAGE. Proteins were aliquoted, flash-frozen in liquid nitrogen and stored at −80 °C. Protein concentrations were determined by measuring the absorbance at 280 nm and assuming an extinction coefficient of 35410 M^{-1} cm^{-1} for HRED 1.1 and HRED1.0 M125W and 29910 M^{-1} cm^{-1} for all other variants. HRED1.0 and HRED1.1 were obtained in yields of 356 mg and 290 mg per litre of culture, respectively.

Library Construction

For saturation mutagenesis, 22 positions were individually randomized using NNK degenerate codons (Table S4). DNA libraries were constructed using overlap extension PCR. The linear fragments and the pET29b(+) vector were digested using *NdeI* and *XhoI* endonucleases, gel-purified and subsequently ligated using T4 DNA ligase.

Recombination of beneficial mutations

The four mutations identified from the library screening were combined by rational recombination of fragments generated by overlap extension PCR. Primers were designed to encode either the parent amino acid or the identified mutation. There primers were used to generate short fragments which were mixed appropriately in overlap extension PCR to generate genes containing all possible combinations of mutations. The linear fragments and the pET29b(+) vector were digested using *NdeI* and *XhoI* endonucleases, gel-purified and subsequently ligated using T4 DNA ligase.

Library screening

For protein expression and screening, all transfer and aliquotting steps were performed using Hamilton liquid-handling robots. Chemically competent *E. coli* BL21(DE3) cells were transformed with the libraries. Freshly transformed clones were used to inoculate 150 µL of LB medium supplemented with 50 µg/mL kanamycin in Corning® Costar® 96-well microtitre round bottom plates. For reference, each plate contained 6 freshly transformed clones of the parent template and 2 clones containing a pET29b(+)_RFP (red fluorescent protein). Plates were incubated overnight at 30 °C, 80 % humidity in a shaking incubator at 850 r.p.m. 20 µL of overnight culture was used to inoculate 480 µL TB medium supplemented with 50 µg/mL kanamycin. The cultures were incubated at 30 °C, 80 % humidity with shaking at 850 r.p.m. until an OD_{600} of about 0.6 was reached, and IPTG was added to a final concentration of 0.1 mM. Induced plates were incubated for 20 h at 30 °C, 80 % humidity with shaking at 850 r.p.m. Cells were harvested by centrifugation at 2,900*g* for 10 min. The supernatant was discarded, the pelleted cells were resuspended in 400 µL lysis buffer (100 mM KPi pH 7.0 buffer supplemented with 1.0 mg/mL lysozyme, 0.5 mg/mL polymyxin B and 10 µg/mL DNaseI) and incubated for 2 h at 30 °C, 80 % humidity with shaking at 850 r.p.m. Cell debris was removed by centrifugation at 2,900*g* for 10 min. 50 µL clarified lysate was transferred to 96-well microtitre plates containing 50 µL reaction mix (final concentrations 5 mM **1**, 500 µM $NADP^+$, 1 mg/mL GDH and 50 mM D-glucose, 100 mM KPi pH 7.0, 5% DMSO). Reactions were incubated for 18 h at 30 °C, 80 % humidity with shaking at 850 r.p.m. The reactions were quenched with 100 µL MeCN and incubated for 2 h at 30 °C, 80 % humidity with shaking at 850 r.p.m. Precipitant was removed by centrifugation at 2,900*g* for 10 min. 100 µL of quenched reaction mix was transferred to fresh 96-well microtitre plates and analysed by UPLC. Reaction conversions of individual variants were normalized to the average of

the 6 parent clones on that plate. The most active variants were rescreened as purified proteins. Proteins were produced and purified as described above, however starter cultures were inoculated from glycerol stocks prepared from the original overnight cultures.

General procedure for analytical scale biotransformations

Analytical scale biotransformations (100 µL) were performed using **1** (5 mM) and the relevant biocatalyst (25-100 µM) in 100 mM KPi pH 7.0 with 5% (v/v) DMSO as a cosolvent. A NADPH cofactor recycling system was used (500 µM $NADP^+$, 1 mg/mL GDH and 50 mM D-glucose, final concentration). Reactions were incubated at 30 °C for specified timepoints with shaking at 850 r.p.m. Following incubation, reactions were quenched with 1 volume MeCN. Quenched reactions were shaken (850 r.p.m) for 2 h. Precipitated protein was removed by centrifugation (14,000*g* for 10 minutes) and supernatants were transferred for UPLC analysis to a fresh plate (see chromatographic analysis). For chiral HPLC analysis, the substrates and products were extracted with 1 volume of ethyl acetate. Precipitated protein was removed by centrifugation (14,000*g* for 10 minutes), the organic phase was separated and directly injected onto the chiral HPLC.

Reaction Component	Concentration of stock solution	Volume added to 100 µL reaction (µL)	Order of addition
Substrate **1** in DMSO	100 mM	5	1
100 mM KPi pH 7.0	N/A	20	2
Cofactor recycling system	4 x stock[a]	25	3
Enzyme	2 x stock (e.g. 200 µM for 100 µM reaction)	50	4

[a] to make 4 mL of 4 x stock of cofactor recycling system: 6 mg $NADP^+$, 16 mg GDH, 144 mg Glucose in 100 mM KPi pH 7.0.

General procedure for substrate scope biotransformations

100 µL scale reactions were performed using the specified hydrazone (**1**-**11**, 5 mM) and HRED1.1 (either 2 or 5 mol%) in 100 mM KPi pH 7.0 with 5% (v/v) DMSO, 500 µM $NADP^+$, 1 mg/mL GDH and 50 mM D-glucose. Reactions were incubated at 30 °C for 18 h with shaking at 850 r.p.m. Following incubation, reactions were quenched with 1 volume MeCN. Quenched reactions were shaken (850 r.p.m) for 2 h. Precipitated protein was removed by centrifugation (14,000*g* for 10 minutes) and supernatants were transferred for UPLC analysis to a fresh plate (see chromatographic analysis). For chiral HPLC analysis, the substrates and products were extracted with 1 volume of ethyl acetate. Precipitated protein was removed by centrifugation (14,000*g* for 10 minutes), the organic phase was separated and directly injected onto the chiral HPLC.

Biotransformation for reductive coupling of Cbz-hydrzaine and acetophenone to form 1a

Analytical scale biotransformations (100 µL) were performed using (benzyloxycarbonyl)hydrazine (25 mM) and acetophenone (5 mM) with HRED1.1 (2 mol%) in 100 mM KPi pH 7.0 with 5% (v/v) DMSO as a cosolvent. A NADPH cofactor recycling system was used (500 µM $NADP^+$, 1 mg/mL GDH and 50 mM D-glucose, final concentration). Reactions were incubated at 30 °C for 18 h with shaking at 850 r.p.m. Following incubation, reactions were quenched with 1 volume MeCN. Quenched reactions were shaken (850 r.p.m) for 2 h. Precipitated protein was removed by centrifugation (14,000*g* for 10 minutes) and supernatants were transferred for UPLC analysis to a

fresh plate (see chromatographic analysis for the substrate scope). Product conversion was calculated using the extinction coefficient of 155 mM^{-1} cm^{-1} for acetophenone, 1187 mM^{-1} cm^{-1} for **1** and 399 mM^{-1} cm^{-1} for **1a** measured at 220 nm.

Chromatographic analysis

UPLC analysis was performed on a 1290 Infinity II Agilent LC system with a Kinetex® 5 µm XB-C18 100 Å LC Column, 50 x 2.1 mm (Phenomenex). For library screening an isocratic method using 33% MeCN in water (0.1% TFA) at 1.2 mL/min for 2.6 minutes was used. Peaks were integrated using Agilent OpenLab software. Product conversions were calculated using the extinction coefficient of 1187 mM^{-1} cm^{-1} for **1** and 399 mM^{-1} cm^{-1} for **1a** measured at 220 nm. For characterising the substrate scope, substrates and products were eluted over 5 minutes using a gradient of 5-95% MeCN in water at 1 mL/min. Peaks were assigned by comparison to chemically synthesised standards and the peak areas were integrated using Agilent OpenLab software. Product conversions were calculated using the extinction coefficients stated in Table S2.

Chiral analysis was performed on a 1200 series Agilent LC system. Products **2a**, **5a**, **6a**, **8a** and **10a** were separated using a Diacel CHIRALPAK® AD-H column (particle size 5 µm, dimensions: 4.6 x 250 mm) over either 30 minutes (**2a** and **8a**) or 50 minutes (**5a**, **6a** and **10a**) with an isocratic method (20:80 EtOH: Hexane 0.1% DEA) at 1 mL/min. Products **1a**, **4a** and **9a** were separated using a Diacel CHIRALCEL OJ-H column (particle size 5 µm, dimensions: 4.6 x 250 mm) over 20 minutes with an isocratic method (20:80 EtOH: Hexane 0.1% DEA) at 1 mL/min. Product **7a** was separated using a Diacel CHIRALPAK® IA column (particle size 5 µm, dimensions: 4.6 x 250 mm) over 30 minutes with an isocratic method (20:80 EtOH: Hexane 0.1% DEA) at 1 mL/min. Product **3a** was separated using a Diacel CHIRALPAK® IE column (particle size 5 µm, dimensions: 4.6 x 250 mm) over 30 minutes with an isocratic method (20:80 EtOH: Hexane) at 1 mL/min. Peaks were assigned by comparison to chemically synthesised standards and the peak areas were integrated using Agilent OpenLab software monitoring at 254 nm.

Chiral analysis of product **11a** was performed using Agilent 7890A GC system, an FID detector, and an Agilent J&W GC column (CP-Chirasil-Dex CB, 25 m x 0.25 mm, 0.25 µm film). A 1 µL sample was injected with a detector temperature 200 °C. The temperature gradient started from 80 °C, then increased to 200 °C (5 °C per min) and held for 2 min. The total run time was 30 min. Peaks were assigned by comparison to chemically synthesised standards and the peak areas were integrated using Agilent OpenLab software.

Mass spectrometry

Purified protein samples were buffer-exchanged into 0.1% acetic acid using a 10k MWCO Vivaspin unit (Sartorius) and diluted to a final concentration of 0.5 mg/mL. MS was performed using a 1200 series Agilent LC system, with a 5 µl injection into 5% MeCN (with 0.1% formic acid), and desalted inline for 1 min. Protein was eluted over 1 min using 95% MeCN with 5% water. The resulting multiply charged spectrum was analysed using an Agilent QTOF 6510 instrument and deconvoluted using Agilent MassHunter software.

Preparative scale biotransformation

A 100 mL preparative scale biotransformation was performed using **1** (134 mg, 5 mM), purified HRED1.1 (100 μM), and cofactor recycling system (500 μM $NADP^+$, 1 mg/mL GDH and 50 mM D-glucose) in 100 mM KPi Ph 7.0 with 5 % (v/v) DMSO as a cosolvent. The reaction was incubated at 30 °C with shaking at 200 r.p.m. for 18 h. An aliquot (100 μL) was removed and quenched with MeCN for UPLC analysis, which showed the reaction had proceeded to 98 % conversion. Ethyl acetate (100 mL) was added to the reaction mixture, vortexed and centrifuged 2,900*g* for 15 min. The organic layer was removed, and the reaction mixture was extracted with ethyl acetate a further two times. The combined organic layers were dried over $MgSO_4$, filtered and the solvent was removed *in vacuo*. The crude product was purified by flash column chromatography (Petroleum/EtOAc: 4:1→Petroleum/EtOAc: 2:1) to give **1a** as a white solid (124 mg, 92% yield, $[\alpha]^{23}_D$ = + 61° (*c* = 1, $CHCl_3$)). Spectral data is consistent with literature values.[2] ^{1}H NMR (400 MHz, Methanol-d_4) δ 7.39 – 7.17 (m, 10H), 5.04 (s, 2H), 4.13 (q, *J* = 6.6 Hz, 1H), 1.29 (d, *J* = 6.7 Hz, 3H); ^{13}C NMR (101 MHz, Methanol-d_4) δ 159.21, 144.34, 138.04, 129.38, 129.34, 128.92, 128.67, 128.33, 128.29, 67.42, 60.67, 21.28. Stereochemistry assigned by comparison to optical rotation values reported in the literature.[2]

General procedure for the preparation of hydrazone 1-11

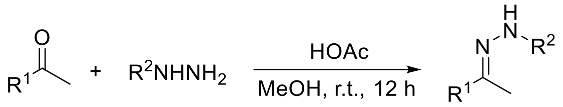

To a solution of ketones (5 mmol) and hydrazides (5 mmol) in MeOH (15 mL) was added HOAc (1 mmol), and the resulting mixture was stirred at room temperature for 12 h. After cooling in an ice bath, the solids precipitated out were separated by filtration and then washed with cold MeOH to give the desired hydrazones. If the precipitation did not occur, the reaction mixture was concentrated in *vacuo* to remove the MeOH, then saturated aqueous $NaHCO_3$ solution (40 mL) was added to the residue obtained. The resulting mixture was extracted with ethyl acetate (3 × 15 mL), and the combined organic layers were washed with brine and dried with Na_2SO_4. After filtration and removal of the solvents in *vacuo*, the residue was purified by flash chromatography on silica gel to give the desired hydrazones.

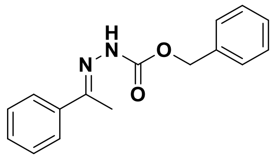

benzyl (*E*)-2-(1-phenylethylidene)hydrazine-1-carboxylate (1): this compound was obtained by filtration as white solid (1.18 g, 87% yield). ^{1}H NMR (500 MHz, Methanol-d_4) δ 7.83 – 7.77 (m, 2H), 7.47 – 7.41 (m, 2H), 7.41 – 7.34 (m, 5H), 7.34 – 7.29 (m, 1H), 5.25 (s, 2H), 2.24 (s, 3H); ^{13}C NMR (126 MHz, Methanol-d_4) δ 156.94, 152.36, 139.68, 137.82, 130.21, 129.53, 129.29, 129.26, 127.56, 68.20, 14.18. HRMS (ESI) m/z: $[M + Na]^+$ Calcd for $C_{16}H_{16}N_2NaO_2$ 291.1104; Found 291.1092.

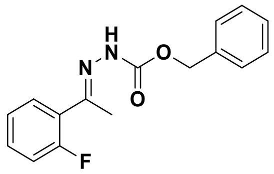

benzyl (*E*)-2-(1-(2-fluorophenyl)ethylidene)hydrazine-1-carboxylate (2): this compound was obtained by filtration as white solid (982 mg, 68% yield). ^{1}H NMR (500 MHz, Methanol-d_4) δ 7.65 – 7.57 (m, 1H), 7.47 – 7.29 (m, 6H), 7.22 – 7.15 (m, 1H), 7.15 – 7.08 (m, 1H), 5.25 (s, 2H), 2.24 (d, J = 2.8 Hz, 3H); ^{13}C NMR (126 MHz, Methanol-d_4) δ 162.00 (d, J_{C-F} = 247.9 Hz), 156.74, 150.43, 137.74, 131.96 (d, J_{C-F} = 8.5 Hz), 131.16 (d, J_{C-F} = 3.3 Hz), 129.54, 129.29, 128.64 (d, J_{C-F} = 12.3 Hz), 125.34 (d, J_{C-F} = 3.4 Hz), 116.84 (d, J_{C-F} = 22.3 Hz), 68.26, 17.52 (d, J_{C-F} = 5.7 Hz); ^{19}F NMR (376 MHz, $CDCl_3$) δ -116.98. HRMS (ESI) m/z: [M + Na]$^+$ Calcd for $C_{16}H_{15}FN_2NaO_2$ 309.1010; Found 309.0999.

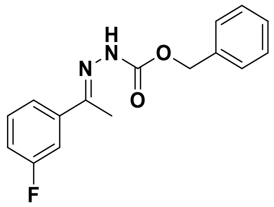

benzyl (*E*)-2-(1-(3-fluorophenyl)ethylidene)hydrazine-1-carboxylate (3): this compound was obtained by filtration as white solid (995 mg, 69% yield). ^{1}H NMR (500 MHz, Methanol-d_4) δ 7.65 – 7.57 (m, 2H), 7.47 – 7.41 (m, 2H), 7.41 – 7.37 (m, 2H), 3.37 – 7.29 (m, 2H), 7.13 – 7.05 (m, 1H), 5.26 (s, 2H), 2.23 (s, 3H); ^{13}C NMR (126 MHz, Methanol-d_4) δ 164.27 (d, J_{C-F} = 243.6 Hz), 156.87, 150.61, 142.12 (d, J_{C-F} = 7.7 Hz), 137.74, 131.01 (d, J_{C-F} = 8.3 Hz), 129.54, 129.29, 129.27, 123.39 (d, J_{C-F} = 2.8 Hz), 116.79 (d, J_{C-F} = 21.7 Hz), 114.11 (d, J_{C-F} = 23.3 Hz), 68.28, 14.00; ^{19}F NMR (376 MHz, $CDCl_3$) δ -115.48. HRMS (ESI) m/z: [M + Na]$^+$ Calcd for $C_{16}H_{15}FN_2NaO_2$ 309.1010; Found 309.0999.

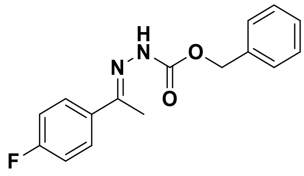

benzyl (*E*)-2-(1-(4-fluorophenyl)ethylidene)hydrazine-1-carboxylate (4): this compound was obtained by filtration as white solid (1.02 g, 71% yield). ^{1}H NMR (500 MHz, Methanol-d_4) δ 7.88 – 7.81 (m, 2H), 7.47 – 7.41 (m, 2H), 7.41 – 7.35 (m, 2H), 7.35 – 7.29 (m, 1H), 7.14 – 7.05 (m, 2H), 5.25 (s, 2H), 2.23 (s, 3H); ^{13}C NMR (126 MHz, Methanol-d_4) δ 164.86 (d, J_{C-F} = 247.5 Hz), 156.96, 151.22, 137.80, 136.00 (d, J_{C-F} = 3.2 Hz), 129.67 (d, J_{C-F} = 8.5 Hz), 129.54, 129.28, 129.26, 116.00 (d, J_{C-F} = 21.9 Hz), 68.23, 14.11; ^{19}F NMR (376 MHz, $CDCl_3$) δ -114.83. HRMS (ESI) m/z: [M + Na]$^+$ Calcd for $C_{16}H_{15}FN_2NaO_2$ 309.1010; Found 309.1002.

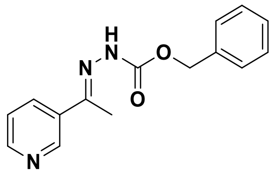

benzyl (*E*)-2-(1-(pyridin-3-yl)ethylidene)hydrazine-1-carboxylate (5): this compound was obtained by filtration as white solid (876 mg, 65% yield). ^{1}H NMR (500 MHz, Methanol-d_4) δ 8.99 –

8.94 (m, 1H), 8.53 – 8.48 (m, 1H), 8.30 – 8.24 (m, 1H), 7.48 – 7.42 (m, 3H), 7.42 – 7.27 (m, 3H), 5.27 (s, 2H), 2.27 (s, 3H); ^{13}C NMR (126 MHz, Methanol-d_4) δ 156.83, 150.05, 148.84, 148.18, 137.68, 135.99, 135.86, 129.55, 129.32, 129.30, 124.92, 68.37, 13.74. HRMS (ESI) m/z: $[M + Na]^+$ Calcd for $C_{15}H_{15}N_3NaO_2$ 292.1056; Found 292.1047.

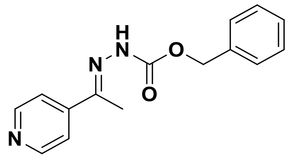

benzyl (*E*)-2-(1-(pyridin-4-yl)ethylidene)hydrazine-1-carboxylate (6): this compound was obtained by filtration as white solid (887 mg, 65% yield). ^{1}H NMR (500 MHz, Methanol-d_4) δ 8.56 – 8.51 (m, 2H), 7.86 – 7.82 (m, 2H), 7.48 – 7.42 (m, 2H), 7.41 – 7.35 (m, 2H), 7.35 – 7.29 (m, 1H), 5.28 (s, 2H), 2.25 (s, 3H); ^{13}C NMR (126 MHz, Methanol-d_4) δ 156.69, 150.25, 148.41, 147.92, 137.61, 129.56, 129.36, 129.33, 122.22, 68.49, 13.25. HRMS (ESI) m/z: $[M + Na]^+$ Calcd for $C_{15}H_{15}N_3NaO_2$ 292.1056; Found 292.1052.

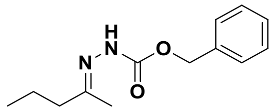

benzyl (*E*)-2-(pentan-2-ylidene)hydrazine-1-carboxylate (7): this compound was obtained by flash chromatography (Petroleum/EtOAc: 4:1→Petroleum/EtOAc: 2/1) on silica gel as white solid (950.1 mg, 81% yield). ^{1}H NMR (400 MHz, Methanol-d_4) δ 7.49 – 7.21 (m, 5H), 5.18 (s, 2H), 2.31 – 2.17 (m, 2H), 1.84 (s, 3H), 1.63 – 1.48 (m, 2H), 0.92 (t, J = 7.4 Hz, 3H); ^{13}C NMR (101 MHz, Methanol-d_4) δ 158.35, 156.67, 137.89, 129.47, 129.17, 67.87, 41.44, 21.05, 15.67, 13.98; HRMS (ESI) m/z: $[M + Na]^+$ Calcd for $C_{13}H_{18}N_2NaO_2$ 257.1260; Found 257.1252.

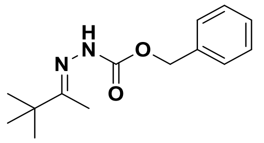

benzyl (*E*)-2-(3,3-dimethylbutan-2-ylidene)hydrazine-1-carboxylate (8): this compound was obtained by filtration as white solid (1.03 g, 83% yield). ^{1}H NMR (500 MHz, $CDCl_3$) δ 7.64 (s, 1H), 7.43 – 7.29 (m, 5H), 5.21 (s, 2H), 1.75 (s, 3H), 1.14 (s, 9H); ^{13}C NMR (126 MHz, $CDCl_3$) δ 159.42, 153.93, 136.20, 128.61, 128.33, 67.23, 38.74, 27.59, 11.22; HRMS (ESI) m/z: $[M + H]^+$ Calcd for $C_{14}H_{21}N_2O_2$ 249.1598; Found 249.1597.

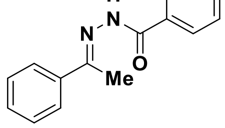

(*E*)-*N'*-(1-phenylethylidene)benzohydrazide (9): this compound was obtained by filtration as white solid (1.04 g, 87% yield). ^{1}H NMR (500 MHz, Methanol-d_4) δ 7.99-7.86 (m, 4H), 7.62-7.56 (m, 1H), 7.54-7.49 (m, 2H), 7.46-7.38 (m, 3H), 2.43 (s, 3H); ^{13}C NMR (126 MHz, Methanol-d_4) δ 167.72, 159.05, 139.32, 134.72, 133.14, 130.94, 129.67, 129.40, 128.86, 128.01, 14.90; HRMS (ESI) m/z: $[M + H]^+$ Calcd for $C_{15}H_{15}N_2O$ 239.1179; Found 239.1178.

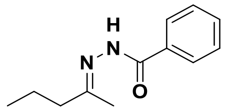

(*E*)-*N'*-(pentan-2-ylidene)benzohydrazide (10): this compound was obtained by flash chromatography (Petroleum/EtOAc: 4:1→Petroleum/EtOAc: 2/1) on silica gel as white solid (860.0 mg, 84% yield). ^{1}H NMR (400 MHz, Methanol-d_4) δ 7.91 – 7.78 (m, 2H), 7.62 – 7.53 (m, 1H), 7.53 – 7.40 (m, 2H), 2.38 (t, J = 7.7 Hz, 2H), 2.03 (s, 3H), 1.73 – 1.55 (m, 2H), 0.98 (t, J = 7.1 Hz, 3H); ^{13}C NMR (101 MHz, Methanol-d_4) δ 167.25, 166.81, 134.75, 132.94, 129.61, 128.67, 41.70, 21.03, 16.51, 14.04; HRMS (ESI) m/z: [M + H]$^+$ Calcd for $C_{12}H_{17}N_2O$ 205.1335; Found 205.1333.

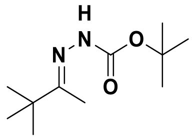

***tert*-butyl (*E*)-2-(3,3-dimethylbutan-2-ylidene)hydrazine-1-carboxylate (11):** this compound was obtained by filtration as white solid (912.0 mg, 85% yield). ^{1}H NMR (400 MHz, Methanol-d_4) δ 1.80 (s, 3H), 1.51 (s, 9H), 1.13 (s, 9H); ^{13}C NMR (101 MHz, Methanol-d_4) δ 161.94, 156.31, 81.40, 39.64, 28.68, 28.12, 12.18; HRMS (ESI) m/z: [M + Na]$^+$ Calcd for $C_{11}H_{22}N_2NaO_2$ 237.1573; Found 237.1563.

General procedure for the preparation of racemic hydrazine products 1a-11a

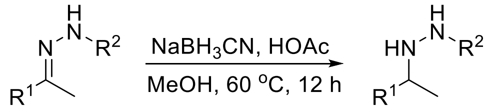

To a solution of hydrazones (1 mmol) in MeOH (10 mL) were added HOAc (17.5 mmol) and $NaBH_3CN$ (4 mmol), and the resulting mixture was stirred at 60 °C for 12 h. After that, the reaction mixture was concentrated in *vacuo* to remove the MeOH, then saturated aqueous $NaHCO_3$ solution (30 mL) was added to the residue obtained. The resulting mixture was extracted with dichloromethane (3 × 10 mL), and the combined organic layers were washed with brine and dried with Na_2SO_4. After filtration and removal of the solvents in vacuo, the residue was purified by flash chromatography on silica gel to give the desired racemic hydrazides.

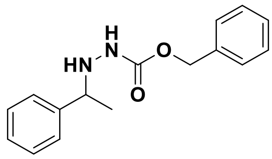

benzyl 2-(1-phenylethyl)hydrazine-1-carboxylate (1a): this compound was obtained by flash chromatography (Petroleum/EtOAc: 4:1→Petroleum/EtOAc: 2/1) on silica gel as colorless oil (258.2 mg, 96% yield). ^{1}H NMR (400 MHz, Methanol-d_4) δ 7.39 – 7.17 (m, 10H), 5.04 (s, 2H), 4.13 (q, J = 6.6 Hz, 1H), 1.29 (d, J = 6.7 Hz, 3H); ^{13}C NMR (101 MHz, Methanol-d_4) δ 159.21, 144.34, 138.04, 129.38, 129.34, 128.92, 128.67, 128.33, 128.29, 67.42, 60.67, 21.28; HRMS (ESI) m/z: [M + Na]$^+$ Calcd for $C_{16}H_{18}N_2NaO_2$ 293.1260; Found 293.1249.

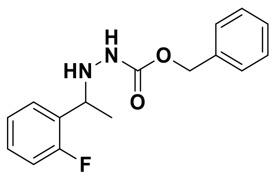

benzyl 2-(1-(2-fluorophenyl)ethyl)hydrazine-1-carboxylate (2a): this compound was obtained by flash chromatography (Petroleum/EtOAc: 4:1→Petroleum/EtOAc: 2/1) on silica gel as colorless oil (262.5 mg, 91% yield). ^{1}H NMR (400 MHz, Methanol-d_4) δ 7.60 – 7.50 (m, 1H), 7.43 – 7.18 (m, 6H), 7.17 – 7.06 (m, 1H), 7.05 – 6.96 (m, 1H), 5.05 (s, 2H), 4.53 (q, J = 6.8 Hz, 1H), 1.30 (d, J = 6.7 Hz, 3H); ^{13}C NMR (101 MHz, Methanol-d_4) δ 162.22 (d, $J_{C\text{-}F}$ = 244.5 Hz), 159.31, 138.04, 131.28 (d, $J_{C\text{-}F}$ = 12.8 Hz), 129.69 (d, $J_{C\text{-}F}$ = 8.4 Hz), 129.38, 129.28 (d, $J_{C\text{-}F}$ = 4.6 Hz), 128.93, 128.68, 125.35 (d, $J_{C\text{-}F}$ = 3.5 Hz), 116.05 (d, $J_{C\text{-}F}$ = 22.5 Hz), 67.46, 53.41, 20.22; HRMS (ESI) m/z: $[M + Na]^+$ Calcd for $C_{16}H_{17}FN_2NaO_2$ 311.1166; Found 311.1155.

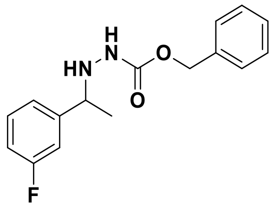

benzyl 2-(1-(3-fluorophenyl)ethyl)hydrazine-1-carboxylate (3a): this compound was obtained by flash chromatography (Petroleum/EtOAc: 4:1→Petroleum/EtOAc: 2/1) on silica gel as colorless oil (279.1 mg, 97% yield). ^{1}H NMR (400 MHz, Methanol-d_4) δ 7.38 – 7.18 (m, 6H), 7.17 – 7.06 (m, 2H), 6.99 – 6.83 (m, 1H), 5.03 (s, 2H), 4.16 (q, J = 6.6 Hz, 1H), 1.26 (d, J = 6.7 Hz, 3H); ^{13}C NMR (101 MHz, Methanol-d_4) δ 164.20 (d, $J_{C\text{-}F}$ = 244.0 Hz), 159.19, 147.53 (d, $J_{C\text{-}F}$ = 6.9 Hz), 137.93, 130.94 (d, $J_{C\text{-}F}$ = 8.2 Hz), 129.35, 128.91, 128.64, 124.17 (d, $J_{C\text{-}F}$ = 2.8 Hz), 114.91 (d, $J_{C\text{-}F}$ = 21.4 Hz), 114.79 (d, $J_{C\text{-}F}$ = 21.8 Hz), 67.43, 60.17, 21.39; HRMS (ESI) m/z: $[M + Na]^+$ Calcd for $C_{16}H_{17}FN_2NaO_2$ 311.1166; Found 311.1155.

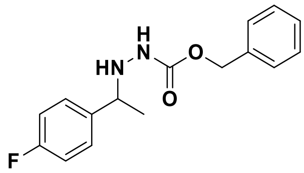

benzyl 2-(1-(4-fluorophenyl)ethyl)hydrazine-1-carboxylate (4a): this compound was obtained by flash chromatography (Petroleum/EtOAc: 4:1→Petroleum/EtOAc: 2/1) on silica gel as white solid (260.1 mg, 90% yield). ^{1}H NMR (400 MHz, $CDCl_3$) δ 7.42 – 7.25 (m, 7H), 7.05 – 6.93 (m, 2H), 6.18 (s, 1H), 5.11 (s, 2H), 4.32 – 4.00 (m, 2H), 1.31 (d, J = 6.6 Hz, 3H); ^{13}C NMR (101 MHz, $CDCl_3$) δ 162.28 (d, $J_{C\text{-}F}$ = 245.3 Hz), 157.29, 138.85, 136.12, 128.85 (d, $J_{C\text{-}F}$ = 8.1 Hz), 128.70, 128.45, 128.26, 115.44 (d, $J_{C\text{-}F}$ = 21.2 Hz), 67.26, 58.90, 21.31; ^{19}F NMR (376 MHz, $CDCl_3$) δ -115.27; HRMS (ESI) m/z: $[M + Na]^+$ Calcd for $C_{16}H_{17}FN_2NaO_2$ 311.1166; Found 311.1158.

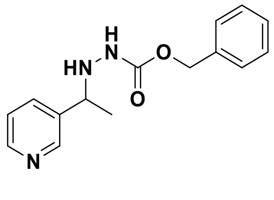

benzyl 2-(1-(pyridin-3-yl)ethyl)hydrazine-1-carboxylate (5a): this compound was obtained by flash chromatography (DCM/MeOH: 60:1→DCM/MeOH: 40/1) on silica gel as yellow oil (231.1 mg, 85% yield). ^{1}H NMR (400 MHz, Methanol-d_4) δ 8.50 (s, 1H), 8.41 (d, J = 4.1 Hz, 1H), 7.87 (d, J = 7.9 Hz, 1H), 7.47 – 7.15 (m, 6H), 5.14 – 4.94 (m, 2H), 4.21 (q, J = 6.6 Hz, 1H), 1.33 (d, J = 6.6 Hz, 3H); ^{13}C NMR (101 MHz, Methanol-d_4) δ 159.35, 149.52, 148.91, 141.09, 138.11, 137.27, 129.43, 128.98, 128.68, 125.17, 67.45, 58.38, 21.19; HRMS (ESI) m/z: [M + Na]$^+$ Calcd for $C_{15}H_{17}N_3NaO_2$ 294.1213; Found 294.1203.

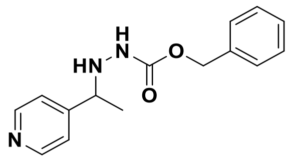

benzyl 2-(1-(pyridin-4-yl)ethyl)hydrazine-1-carboxylate (6a): this compound was obtained by flash chromatography (DCM/MeOH: 60:1→DCM/MeOH: 40/1) on silica gel as pale yellow solid (204.8 mg, 75% yield). ^{1}H NMR (400 MHz, Methanol-d_4) δ 8.42 (d, J = 5.1 Hz, 2H), 7.43 (d, J = 4.0 Hz, 2H), 7.37 – 7.11 (m, 5H), 5.13 – 4.96 (m, 2H), 4.20 (q, J = 6.7 Hz, 1H), 1.29 (d, J = 6.7 Hz, 3H); ^{13}C NMR (101 MHz, Methanol-d_4) δ 159.33, 155.64, 149.94, 138.07, 129.42, 128.99, 128.71, 124.19, 67.48, 59.86, 21.20; HRMS (ESI) m/z: [M + Na]$^+$ Calcd for $C_{15}H_{17}N_3NaO_2$ 294.1213; Found 294.1201.

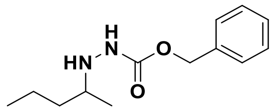

benzyl 2-(pentan-2-yl)hydrazine-1-carboxylate (7a): this compound was obtained by flash chromatography (Petroleum/EtOAc: 4:1→Petroleum/EtOAc: 2/1) on silica gel as white solid (224.3 mg, 95% yield). ^{1}H NMR (400 MHz, Methanol-d_4) δ 7.44 – 7.24 (m, 5H), 5.11 (s, 2H), 3.00 – 2.82 (m, 1H), 1.51 – 1.26 (m, 3H), 1.26 – 1.17 (m, 1H), 1.00 (d, J = 6.3 Hz, 3H), 0.92 (t, J = 7.1 Hz, 3H); ^{13}C NMR (101 MHz, Methanol-d_4) δ 159.38, 138.18, 129.45, 129.04, 128.84, 67.57, 56.33, 37.90, 20.10, 18.43, 14.61; HRMS (ESI) m/z: [M + Na]$^+$ Calcd for $C_{13}H_{20}N_2NaO_2$ 259.1417; Found 259.1409.

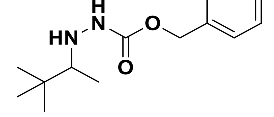

benzyl 2-(3,3-dimethylbutan-2-yl)hydrazine-1-carboxylate (8a): this compound was obtained by flash chromatography (Petroleum/EtOAc: 4:1→Petroleum/EtOAc: 2/1) on silica gel as white solid (230.5 mg, 92% yield). ^{1}H NMR (400 MHz, Methanol-d_4) δ 7.44 – 7.25 (m, 5H), 5.10 (s, 2H), 2.64 (q, J = 6.5 Hz, 1H), 0.97 (d, J = 6.5 Hz, 3H), 0.93 (s, 9H); ^{13}C NMR (101 MHz, Methanol-d_4) δ 159.38, 138.16, 129.45, 129.03, 128.82, 67.56, 65.40, 34.20, 26.87, 14.02; HRMS (ESI) m/z: [M + H]$^+$ Calcd for $C_{14}H_{23}N_2O_2$ 251.1754; Found 251.1748.

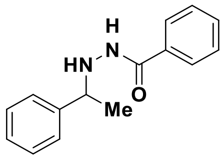

***N'*-(1-phenylethyl)benzohydrazide (9a)**: this compound was obtained by flash chromatography (Petroleum/EtOAc: 4:1→Petroleum/EtOAc: 2/1) on silica gel as white solid (219.3 mg, 91% yield). ^{1}H NMR (400 MHz, Methanol-*d*$_4$) δ 7.69-7.61 (m, 2H), 7.52-7.46 (m, 1H), 7.45-7.36 (m, 4H), 7.36-7.29 (m, 2H), 7.29-7.22 (m, 1H), 4.21 (q, *J* = 6.6 Hz, 1H), 1.42 (d, *J* = 6.6 Hz, 3H); ^{13}C NMR (101 MHz, Methanol-*d*$_4$) δ 169.48, 144.30, 134.35, 132.73, 129.50, 129.45, 128.53, 128.34, 128.21, 61.10, 21.27; HRMS (ESI) m/z: [M + Na]$^+$ Calcd for $C_{15}H_{16}N_2NaO$ 263.1155; Found 263.1153.

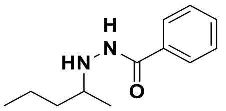

***N'*-(pentan-2-yl)benzohydrazide (10a)**: this compound was obtained by flash chromatography (Petroleum/EtOAc: 4:1→Petroleum/EtOAc: 2/1) on silica gel as white solid (191.5 mg, 93% yield). ^{1}H NMR (400 MHz, Methanol-*d*$_4$) δ 7.84 – 7.75 (m, 2H), 7.57 – 7.50 (m, 1H), 7.50 – 7.41 (m, 2H), 3.11 – 2.91 (m, 1H), 1.63 – 1.51 (m, 1H), 1.51 – 1.27 (m, 3H), 1.10 (d, *J* = 6.3 Hz, 3H), 0.95 (t, *J* = 7.1 Hz, 3H); ^{13}C NMR (101 MHz, Methanol-*d*$_4$) δ 169.20, 134.31, 132.78, 129.59, 128.23, 56.83, 38.25, 20.21, 18.80, 14.60; HRMS (ESI) m/z: [M + H]$^+$ Calcd for $C_{12}H_{19}N_2O$ 207.1492; Found 207.1491.

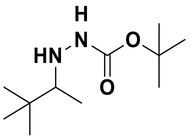

***tert*-butyl 2-(3,3-dimethylbutan-2-yl)hydrazine-1-carboxylate (11a):** this compound was obtained by flash chromatography (Petroleum/EtOAc: 4:1→Petroleum/EtOAc: 2/1) on silica gel as white solid (203.5 mg, 94% yield). ^{1}H NMR (400 MHz, Methanol-*d*$_4$) δ 2.60 (q, *J* = 6.5 Hz, 1H), 1.45 (s, 9H), 0.97 (d, *J* = 6.5 Hz, 3H), 0.93 (s, 9H); ^{13}C NMR (101 MHz, Methanol-*d*$_4$) δ 158.83, 80.67, 65.40, 34.18, 28.77, 26.90, 13.96; HRMS (ESI) m/z: [M + Na]$^+$ Calcd for $C_{11}H_{24}N_2NaO_2$ 239.1730; Found 239.1723.

Expression and Purification of HRED1.1 for X-ray Crystallography

The sequence encoding HRED1.1 was cloned into the pETYSBLIC-3C plasmid using established protocols and the primers listed in Table S4.[3] The recombinant plasmid was then transformed *E. coli* BL21 (DE3) competent cells for gene expression. Pre-cultures were grown in LB medium (10 mL) containing 30 μg/mL kanamycin for 18 h at 37 °C with shaking at 180 r.p.m. 500 mL TB cultures were inoculated with the pre-culture (10 mL) and incubated at 37 °C, with shaking at 230 r.p.m. until an OD_{600} of 0.7 was reached. Expression of HRED1.1 was induced by addition of IPTG (0.5 mM) and shaking was continued overnight at 16 °C at 230 r.p.m. Cells were then harvested by centrifugation at 5000*g* for 20 min and resuspended in 0.1 M Tris-HCl buffer pH 8.0, containing

300 mM NaCl and 30 mM imidazole (His Buffer A). Cells were disrupted, and the suspension was centrifuged at 20,000*g* for 1 h at 4 °C to yield a clear lysate. The N-terminal His6-tagged protein was purified using immobilised-metal affinity chromatography (IMAC) using Ni-NTA column, followed by size exclusion chromatography (SEC). The lysate was loaded onto a pre-equilibrated Histrap™ FF crude 5 mL column, followed by washing with His Buffer A. The bound protein was eluted with a step profile with 500 mM imidazole. Fractions were analysed by 12% acrylamide SDS PAGE and those containing HRED1.1 were pooled, HRV3C protease (1:50 ratio) added, in order to cleave the His-tag, and the protein dialyzed overnight against His buffer A. The cleaved HRED1.1 was then loaded onto a Histrap™ FF crude 5 mL column, with only the flow-through being collected. His-tag free HRED1.1 was centrifugally concentrated (10 kDa MWCO Vivaspin) and loaded onto a HiLoad 16/600 Superdex 75 gel filtration column pre-equilibrated with buffer containing 50 mM HEPES, pH 7.0 and 300 mM NaCl. Fractions were analysed by 12% acrylamide SDS PAGE and those containing the enzyme were pooled and concentrated to 20 mg/mL for crystallization screening.

Crystallisation, Data Collection and Structure Solution and Refinement

Initial screening of crystallization conditions was performed using commercially available INDEX (Hampton Research), PACT premier and CSSI/II (Molecular Dimensions) screens in 96-well sitting drop trays using a MosquitoTM Robot (SPT Labtech) and drops containing 150 nL each of protein and precipitant solutions. Optimization was carried out in a 24-well hanging-drop format in Linbro dishes and drops containing 1 µL each of protein and precipitant solutions to obtain crystals for X-ray diffraction studies.

Crystals of HRED1.1 were grown from protein concentrated to 20 mg/mL in 50 mM Tris-HCl buffer at pH 7.1 containing 2 mM $NADP^+$, 300 mM NaCl and 10% glycerol. The best crystals were obtained in conditions containing 2 M ammonium sulfate in bis-Tris buffer at pH 6.5. Crystals for diffraction testing were harvested directly into liquid nitrogen with nylon CryoLoopsTM (Hampton Research), using the mother liquor with 10% ethylene glycol as cryoprotectant.

Data collection, structure solution and refinement

The dataset described in this report was collected at the Diamond Light Source, Didcot, Oxfordshire, U.K. on beamline I03. Data were processed and integrated using XDS[4] and scaled using SCALA[5] included in the Xia2[6] processing system. Data collection statistics are provided in Table S3. All crystals were obtained in space group I213, with two molecules in the asymmetric unit (asu), forming one dimer. The Matthews coefficient of 4.24 for the crystals suggested 4 molecules in the asu, but the water content of the crystals was high, at 71.01%. The structure of HRED1.1 was solved by molecular replacement using MOLREP[7] and wild-type IR361 (PDB 7OSN)[8] as the search model. The structure was built and refined using iterative cycles in Coot[9] and REFMAC.[10] Residues and side-chains were well resolved in subunit A, but some residues and many side chains were missing especially in the N-terminal domain of subunit B. The average B-factors in the final structure are consequently high (>100). However, following building and refinement of the protein and water molecules, residual density was clearly observed in the omit maps at the dimer interface that was successfully modelled as $NADP^+$. In addition, omit density was also observed at the sites of most structurally consequential mutations of M125W and I127F (Figure S11). The final structures of HRED1.1 exhibited % Rcryst /Rfree values of 22.4/30.1.

Refinement statistics for the structures are presented in Table S3. The structure of HRED1.1 has been deposited in the Protein Databank (PDB) with the accession code 9H4F.

Modelling

Automated docking was performed using AUTODOCK VINA.[11] Coordinates for the ligand were prepared using the ligand builder in ACEDRG.[12] The appropriate pdbqt files for the dimeric model of HRED1.1 and the ligand **1** were prepared in AUTODOCK Tools. The active site of HRED1.1 was contained in a grid size of 12 Å × 15 Å × 12 Å (corresponding to x, y, z) with 1 Å spacing, centred around the catalytic centre at positions -6.82 Å × 31.91 Å × -34.21 Å (corresponding to x, y, z), which was generated using AutoGrid in the AUTODOCK Tools interface. The dockings were performed by VINA, therefore the posed dockings were below 2 Å r.m.s.d. The results generated by VINA were visualised in AUTODOCK Tools 1.5.6 where the ligand conformations were assessed based upon the lowest VINA energies.

Supplementary Figures

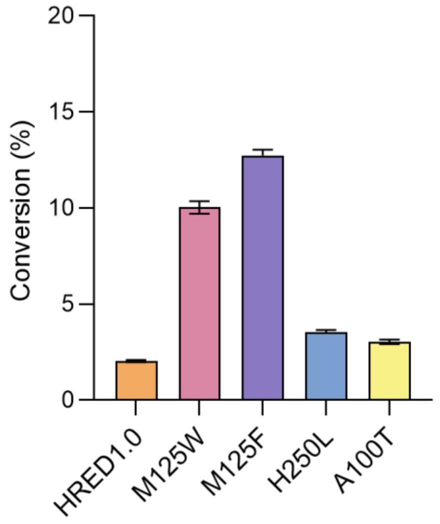

Figure S1: Conversions of HRED1.0 and the four mutants identified during evolution. Biotransformations were performed using **1** (5 mM) and enzyme (25 µM) in 100 mM KPi pH 7.0 with $NADP^+$ (500 µM), GDH (1 mg/mL), D-glucose (50 mM) and 5% (v/v) DMSO as a cosolvent. Reactions were analyzed following 25 h incubation at 30 °C. Error bars represent the standard deviation of measurements made in triplicate.

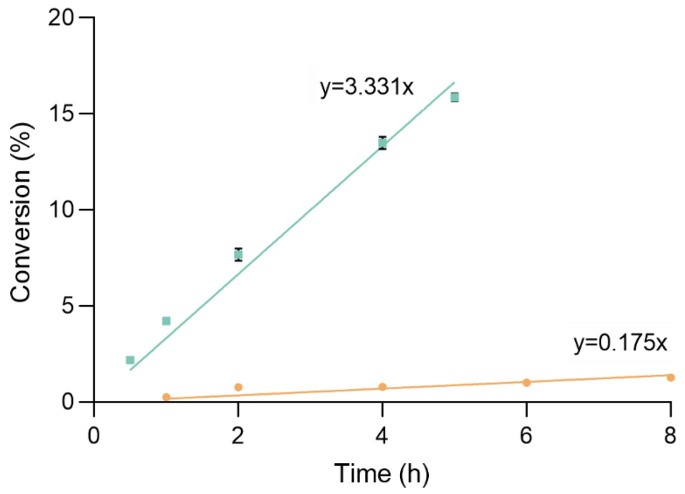

Figure S2: Rate improvement of HRED1.1 as compared to HRED1.0. Biotransformations were performed using **1** (5 mM) with either HRED1.0 or HRED1.1 (orange and green, respectively, 25 µM) in 100 mM KPi pH 7.0 with $NADP^+$ (500 µM), GDH (1 mg/mL), D-glucose (50 mM) and 5% (v/v) DMSO as a cosolvent. Reactions were analyzed following incubation at 30 °C. Error bars represent the standard deviation of measurements made in triplicate.

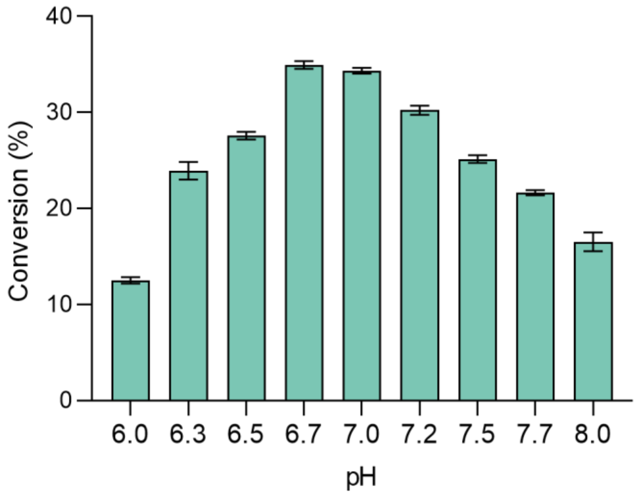

Figure S3: pH profile of HRED1.1. Conversions of HRED1.1 across a range of pHs between 6.0 and 8.0. Biotransformations were performed using **1** (5 mM) and HRED1.1 (25 μM) in 100 mM KPi at the stated pH with $NADP^+$ (500 μM), GDH (1 mg/mL), D-glucose (50 mM) and 5% (v/v) DMSO as a cosolvent. Reactions were analyzed following 25 h incubation at 30 °C. Error bars represent the standard deviation of measurements made in triplicate.

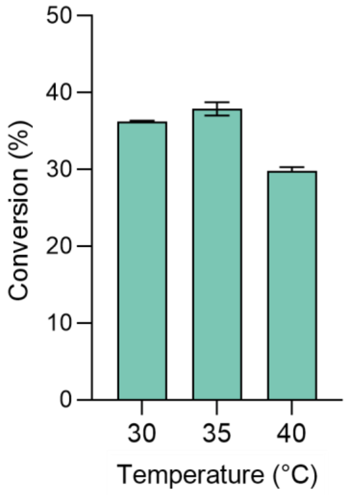

Figure S4: Temperature profile of HRED1.1. Biotransformations were performed using **1** (5 mM) and HRED1.1 (25 μM) in 100 mM KPi pH 7.0 with $NADP^+$ (500 μM), GDH (1 mg/mL), D-glucose (50 mM) and 5% (v/v) DMSO as a cosolvent. Reactions were analyzed following 25 h incubation at either 30, 35 or 40 °C. Error bars represent the standard deviation of measurements made in triplicate.

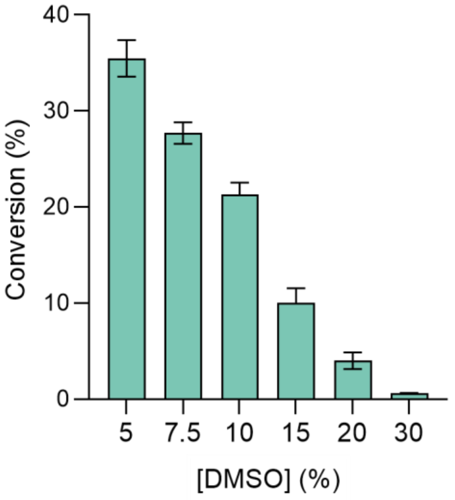

Figure S5: Effect of cosolvent on HRED1.1 activity. Biotransformations were performed using **1** (5 mM) and HRED1.1 (25 µM) in 100 mM KPi pH 7.0 with $NADP^+$ (500 µM), GDH (1 mg/mL), D-glucose (50 mM) and 5-30% (v/v) DMSO as a cosolvent. Reactions were analyzed following 25 h incubation at 30 °C. Error bars represent the standard deviation of measurements made in triplicate.

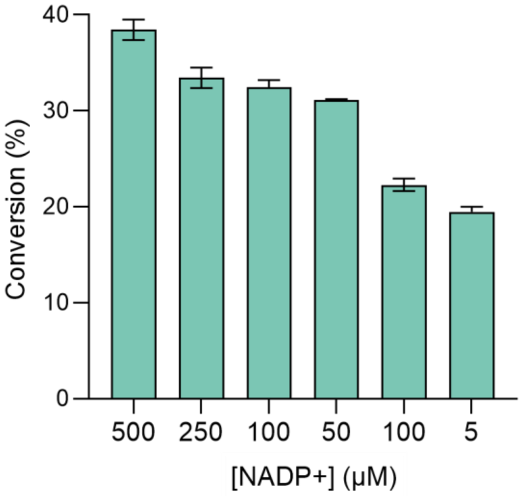

Figure S6: Effect of NADP+ concentration on HRED 1.1. Biotransformations were performed using **1** (5 mM) and HRED1.1 (25 µM) in 100 mM KPi pH 7.0 with $NADP^+$ (500-5 µM), GDH (1 mg/mL), D-glucose (50 mM) and 5% (v/v) DMSO as a cosolvent. Reactions were analyzed following 25 h incubation at 30 °C. Error bars represent the standard deviation of measurements made in triplicate.

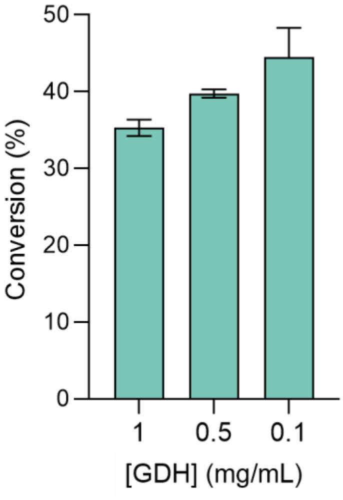

Figure S7: Effect of GDH concentration on HRED1.1. Biotransformations were performed using **1** (5 mM) and HRED1.1 (25 µM) in 100 mM KPi pH 7.0 with NADP+ (500 µM), GDH (1-0.1 mg/mL), D-glucose (50 mM) and 5% (v/v) DMSO as a cosolvent. Reactions were analyzed following 25 h incubation at 30 °C. Error bars represent the standard deviation of measurements made in triplicate.

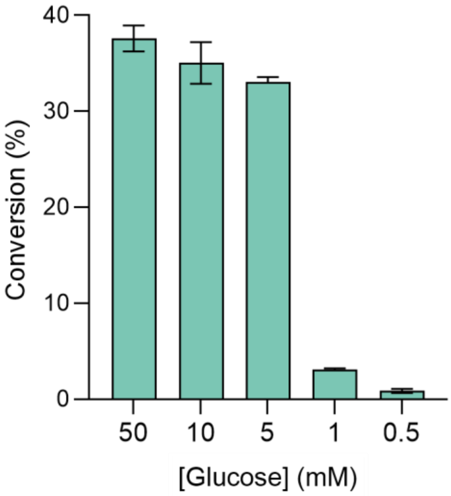

Figure S8: Effect of glucose concentration on HRED1.1. Biotransformations were performed using **1** (5 mM) and HRED1.1 (25 µM) in 100 mM KPi pH 7.0 with NADP+ (500 µM), GDH (1 mg/mL), D-glucose (50-0.5 mM) and 5% (v/v) DMSO as a cosolvent. Reactions were analyzed following 25 h incubation at 30 °C. Error bars represent the standard deviation of measurements made in triplicate.

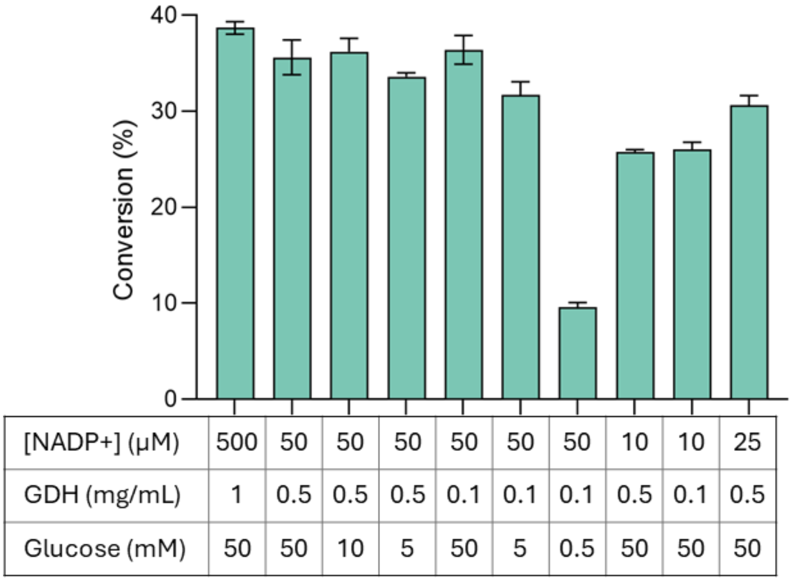

[NADP+] (μM)	500	50	50	50	50	50	50	10	10	25
GDH (mg/mL)	1	0.5	0.5	0.5	0.1	0.1	0.1	0.5	0.1	0.5
Glucose (mM)	50	50	10	5	50	5	0.5	50	50	50

Figure S9: Optimization of NADPH cofactor recycling system. Biotransformations were performed using **1** (5 mM) and HRED1.1 (25 μM) in 100 mM KPi pH 7.0 with the stated cofactor recycling system concentrations and 5% (v/v) DMSO as a cosolvent. Reactions were analyzed following 25 h incubation at 30 °C. Error bars represent the standard deviation of measurements made in triplicate.

A

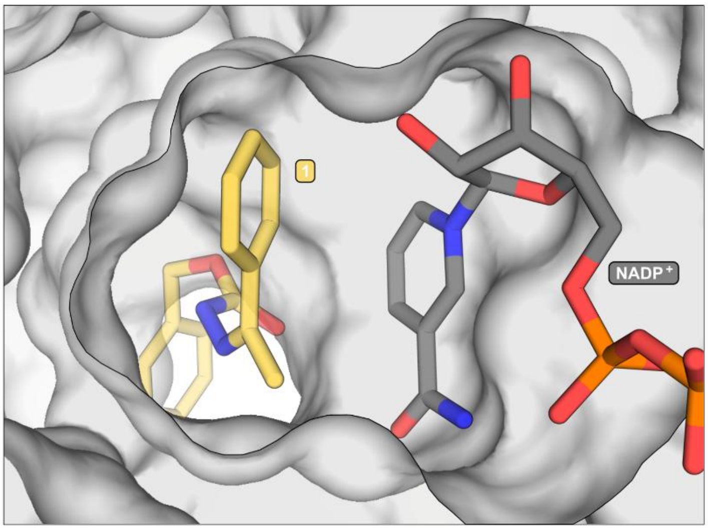

B

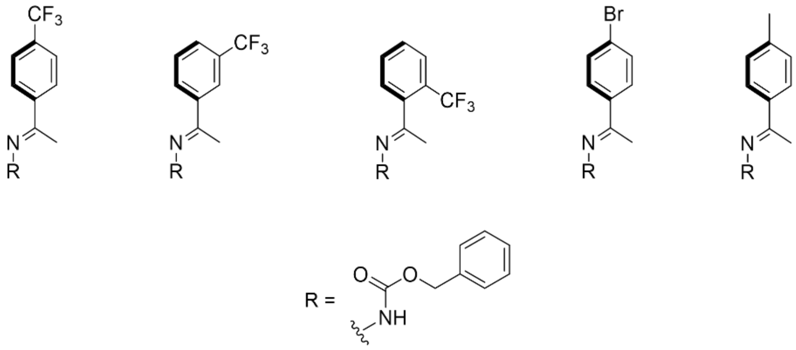

Figure S10: Steric constraints within the active site pocket of HRED1.1. A. Crystal structure of HRED1.1 (shown as grey surface) with hydrazone **1** (atom-coloured sticks, yellow) modelled into the active site. **B.** Hydrazone substrates with larger aryl-substituents that are not accepted by HRED1.1.

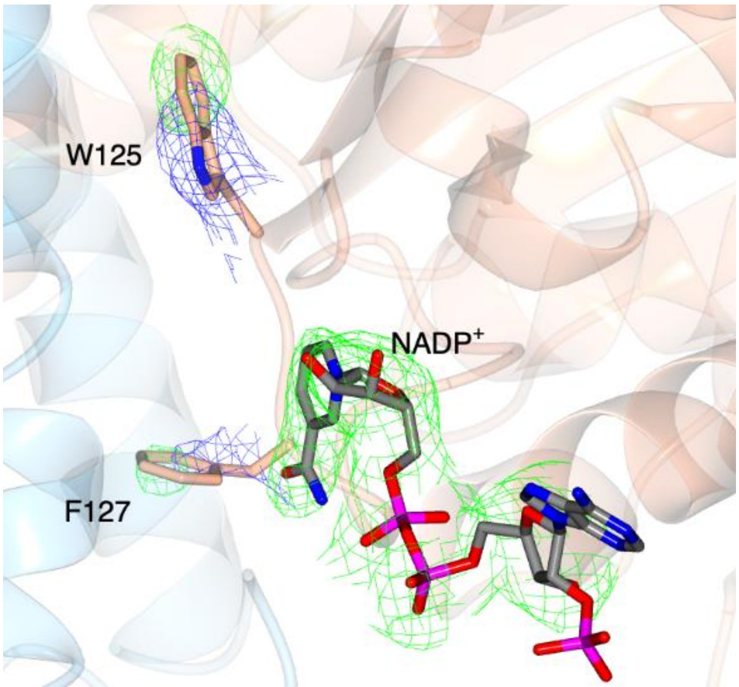

Figure S11: Omit maps prior to refinement of mutated residues and $NADP^+$. The electron density maps in blue and green correspond to the $2F_o$-F_c and F_o-F_c maps at levels of 1.0 σ and 2.5 σ respectively.

Supplementary Tables

Table S1: Conversions of HRED1.0, HRED1.1 and variants. Biotransformations were performed using **1** (5 mM) and enzyme (100-25 µM) in 100 mM KPi pH 7.0 with $NADP^+$ (500 µM), GDH (1 mg/mL), D-glucose (50 mM) and 5% (v/v) DMSO as a cosolvent. Reactions were analyzed following 18 or 25 h incubation at 30 °C. * = preparative scale biotransformation after 18 h incubation at 30 °C using **1** (5 mM) and enzyme (100 µM) in 100 mM KPi pH 7.0 with $NADP^+$ (500 µM), GDH (1 mg/mL), D-glucose (50 mM) and 5% (v/v) DMSO as a cosolvent. N.D. = not determined, reaction only ran as a single measurement. *† = preparative scale biotransformation after 18 h incubation at 30 °C using **1** (5 mM) and enzyme (100 µM) in 100 mM KPi pH 7.0 with $NADP^+$ (50 µM), GDH (0.1 mg/mL), D-glucose (50 mM) and 5% (v/v) DMSO as a cosolvent. N.D. = not determined, reaction only ran as a single measurement.

Variant	Enzyme Loading (mol %)	Time (h)	Conversion (%)	S.D.	TON
HRED1.0	0.5	25	2.0	0.1	4
HRED1.0	2	18	4.7	0.3	2
HRED1.0_M125W	0.5	25	10.0	0.3	20
HRED1.0_M125F	0.5	25	12.7	0.2	25
HRED1.0_H250L	0.5	25	3.5	0.1	7
HRED1.0_A100T	0.5	25	3.0	0.1	6
HRED1.1	0.5	25	39.6	0.5	80
HRED1.1	2	18	99.3	0.1	50
HRED1.1*	2	18	98.1 (92% isolated yield)	N.D.	49
HRED1.1*†	2	18	79.9	N.D	40

Table S2: Substrate scope of HRED1.1. Biotransformations were performed using the specified substrate (5 mM) and HRED1.1 in 100 mM KPi pH 7.0 with $NADP^+$ (500 µM), GDH (1 mg/mL), D-glucose (50 mM) and 5% (v/v) DMSO as a cosolvent. Reactions were analyzed following 25 h incubation at 30 °C.

Substrate	Product	Enzyme Loading (mol%)	Conversion (%)	S.D.	*e.e.* (%)	Substrate Extinction Coefficient[a] (mM^{-1} cm^{-1})	Product Extinction Coefficient[a] (mM^{-1} cm^{-1})
**1**	**1a**	2	99.3	0.1	>99	1187	399
**2**	**2a**	2	96.9	0.7	>99	731	239
**3**	**3a**	5	98.7	0.1	>99	770	234
**4**	**4a**	5	13.8	0.1	>99	753	247
**5**	**5a**	5	28.9	2.0	>99	744	219
**6**	**6a**	5	41.5	0.6	>99	634	234
**7**	**7a**	5	97.9	0.1	>99	726	137
**8**	**8a**	2	98.6	0.2	>99	743	253
**9**	**9a**	5	10.8	0.4	>99	742	686
**10**	**10a**	5	78.6	0.1	>99	329	531
**11**	**11a**	2	91.0	0.1	>99	646	13
**One-pot**[b]	**1a**	2	44.1	2.3	>99	Acetophenone 155<br>**1**, 1187	399

[a] Extinction coefficients all measured at 220 nm.

[b] one-pot reductive coupling between acetophenone (5 mM) and (benzyloxycarbonyl)hydrazine (25 mM).

Table S3: Data collection and refinement statistics for HRED1.1 (IR361-M125W/I127F/L179V/H250L). Numbers in brackets refer to data for highest resolution shells.

	**IR361-M125W/I127F/L179V/H250L 19-09-24 - 9478**
Beamline	I03
Wavelength (Å)	0.97626
Resolution (Å)	130.30-2.77 (3.34-2.77)
Space Group	$I2_13$
Unit cell (Å)	a = b = c = 184.27; α = β = γ = 90.00°
No. of molecules in the asymmetric unit	2
Unique reflections	15775 (789)
Completeness (%)	93.7 (47.0)
R_{merge} (%)	0.10 (2.64)
$R_{p.i.m.}$	0.02 (0.42)
Multiplicity	40.4 (42.0)
$<I/\sigma(I)>$	37.6 (1.7)
Overall B from Wilson plot ($Å_2$)	115
$CC_{1/2}$	1.00 (0.78)
R_{cryst}/ R_{free} (%)	22.0/30.0
r.m.s.d 1-2 bonds (Å)	0.007
r.m.s.d 1-3 angles (°)	1.71
Avge main chain B ($Å^2$)	137
Avge side chain B ($Å^2$)	132
Avge waters B ($Å^2$)	-
Avge ligand B ($Å^2$)	113

Table S4: Table of primers

Evolution Primers	Sequence
IRED361_GG_F	atactacggtctcaaggaATGAGTGACCCTAATGCGG
IRED361_GG_R	gcattacggtctcgggaaccCGCGGTGGGCTTA
M19_F	GTTGGCTTGGGTCTGNNKGGTCAGGCGCTGG
M19_R	CAGACCCAAGCCAAC
T99_F	CTGGTGAATTTGACGNNKGCCACCAGCACCCAA
T99_R	CGTCAAATTCACCAGGACG
A100_F	GTGAATTTGACGACGNNKACCAGCACCCAAGCG
A100_R	CGTCGTCAAATTCACCAGG
M125_F	TTAGACGGCGCAATCNNKGCTTTTCCACCAGTTATTGGTA
M125_R	GATTGCGCCGTCTAAATACG
A126_F	GACGGCGCAATCATGNNKTTTCCACCAGTTATTGGTACC
A126_R	CATGATTGCGCCGTC
F127_F	GGCGCAATCATGGCTNNKCCACCAGTTATTGGTACCG
F127_R	AGCCATGATTGCGCC
P128_F	GCAATCATGGCTTTTNNKCCAGTTATTGGTACCGATG
P128_R	AAAAGCCATGATTGCGCC
P129_F	ATCATGGCTTTTCCANNKGTTATTGGTACCGATGGCG
P129_R	TGGAAAAGCCATGATTGCG
D176_F	TTATCATCATTGTACNNKATGGCCGTTCTGGGAAT
D176_R	GTACAATGATGATAACCCATGATC
V179_F	TTGTACGACATGGCCNNKCTGGGAATCATGTGGGGT
V179_R	GGCCATGTCGTACAATGA
L180_F	TACGACATGGCCGTTNNKGGAATCATGTGGGGTATTCTG
L180_R	AACGGCCATGTCGTACAAT
M183_F	GCCGTTCTGGGAATCNNKTGGGGTATTCTGAATGGC
M183_R	GATTCCCAGAACGGCC
W184_F	GTTCTGGGAATCATGNNKGGTATTCTGAATGGCTTTCTG
W184_R	CATGATTCCCAGAACGGC
M214_F	CCGTTAGCAAACACCNNKATTTCGGCGATCACAGAA
M214_R	GGTGTTTGCTAACGGCG
Y221_F	TCGGCGATCACAGAANNKGTGACTGCGTACGCC
Y221_R	TTCTGTGATCGCCGAAAT
Y225_F	GAATACGTGACTGCGNNKGCCCCACAAGTTGATGAA
Y225_R	CGCAGTCACGTATTCTGT
D238_F	CGCTACGAAGCAACANNKGCCACCATGACCGTACAT
D238_R	TGTTGCTTCGTAGCGTCC
A239_F	TACGAAGCAACAGATNNKACCATGACCGTACATCAG
A239_R	ATCTGTTGCTTCGTAGCG
V243_F	GATGCCACCATGACCNNKCATCAGGCCGCGATG
V243_R	GGTCATGGTGGCATCTG
H244_F	GCCACCATGACCGTANNKCAGGCCGCGATGGAG
H244_R	TACGGTCATGGTGGCATC

A247_F	ACCGTACATCAGGCCNNKATGGAGCACTTGGCTGAA
A247_R	GGCCTGATGTACGGTCAT
H250_F	CAGGCCGCGATGGAGNNKTTGGCTGAAGAAAGCGAACAT
H250_R	CTCCATCGCGGCCTG
**Shuffling Primers**	
A100T_F	GTGAATTTGACGACGACAACCAGCACCCAAGCG
M125F_F	TTAGACGGCGCAATCTTTGCTTTTCCACCAGTTATTGGTA
M125W_F	TTAGACGGCGCAATCTGGGCTTTTCCACCAGTTATTGGTA
H250L_F	CAGGCCGCGATGGAGCTCTTGGCTGAAGAAAGCGAACAT
**Crystallography Primers**	
CCGAAAGGAAGCTGAGTTGG (LIC3C F)	TTCCAGGGACCAGCAATGAGTGACCCTAATGCGGATC
TGCTGGTCCCTGGAACAG (LIC3CR)	TCAGCTTCCTTTCGGTTACGCGGTGGGCTTACGG

Table S5: Experimental and calculated masses of HRED1.0 and HRED1.1.

Variant	Observed Mass	Expected Mass
HRED1.0	32489.7 (-Met)	32619.93
HRED1.1	32520.7 (-Met)	32650.97

DNA and Protein Sequences

HRED1.1 DNA sequence

ATGAGTGACCCTAATGCGGATCGCCCACCAGTGACAGTTGTTGGCTTGGGTCTGATGGGTCAGGCG
CTGGCAGCCGCATTCCTTAAGGGCGGTCACCCGACCACAGTCTGGAACCGTTCCCCTGAGAAAGC
CGAACGTTTAGTGGCCGATGGAGCGGTTTTGGCCGATACTTTAGAATCCGCCGTAACGGCCAGCCC
ACTGGTCATTGTGTGCGTGTCTGATTACGATGCCGTGCACGAATTGATTCGTCCGGTTGAATCAGCGTT
AGCGGGCCGCGTCCTGGTGAATTTGACGACGGCCACCAGCACCCAAGCGCGCGAAACTGCTGAA
TGGGCCGCGCAGCGTAACATCCCGTATTTAGACGGCGCAATCTGGGCTTTTCCACCAGTTATTGGTA
CCGATGGCGCAGTTTTGCTGTACAGTGGCCACAAATCTGCCTTTGAGGCACATGAGTCTACCTTAAAA
GCTATCGCCCCGGCCGCGACCACCTATCTGGAAGAAGATCATGGGTTATCATCATTGTACGACATGG
CCGTTCTGGGAATCATGTGGGGTATTCTGAATGGCTTTCTGCATGGCGCTGCGCTGCTGGGCACTGC
TAAAGTTAAAGCGGAGACGTTCGCGCCGTTAGCAAACACCATGATTTCGGCGATCACAGAATACGTG
ACTGCGTACGCCCCACAAGTTGATGAAGGACGCTACGAAGCAACAGATGCCACCATGACCGTACAT
CAGGCCGCGATGGAGCTCTTGGCTGAAGAAAGCGAACATTTGGGCATTCATTCAGAGCTTCCTCGCT
TCTTCAAGACCCTCGCCGACCGCGCTGTTGCAGATGGACACGCAGAAAACTCCTACGCGGCGATG
ATTGAGTTGTTCCGTAAGCCCACCGCGCTCGAGCACCACCACCACCACCAC

HRED1.1 Protein Sequence

MSDPNADRPPVTVVGLGLMGQALAAAFLKGGHPTTVWNRSPEKAERLVADGAVLADTLESAVTASPLVI
VCVSDYDAVHELIRPVESALAGRVLVNLTTATSTQARETAEWAAQRNIPYLDGAI**W**AFPPVIGTDGAVLLYS
GHKSAFEAHESTLKAIAPAATTYLEEDHGLSSLYDMAVLGIMWGILNGFLHGAALLGTAKVKAETFAPLAN
TMISAITEYVTAYAPQVDEGRYEATDATMTVHQAAME**L**LAEESEHLGIHSELPRFFKTLADRAVADGHAEN
SYAAMIELFRKPTALEHHHHHH

NMR Spectra

benzyl (*E*)-2-(1-phenylethylidene)hydrazine-1-carboxylate (1)

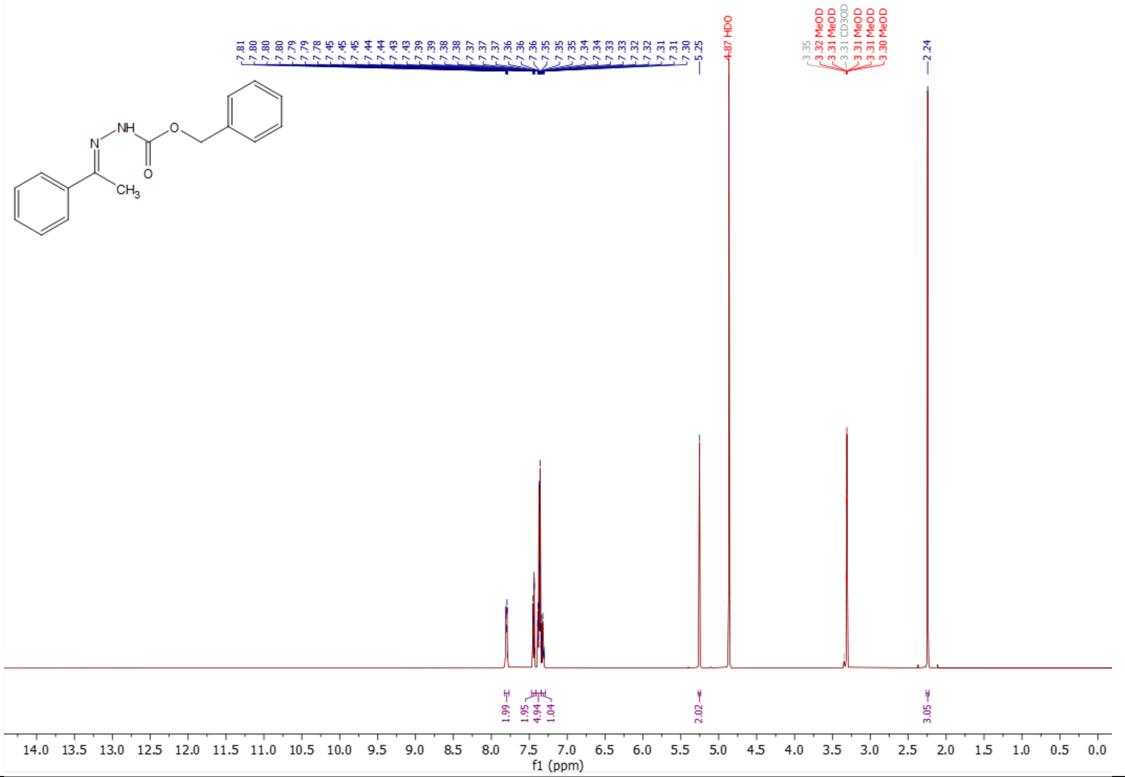

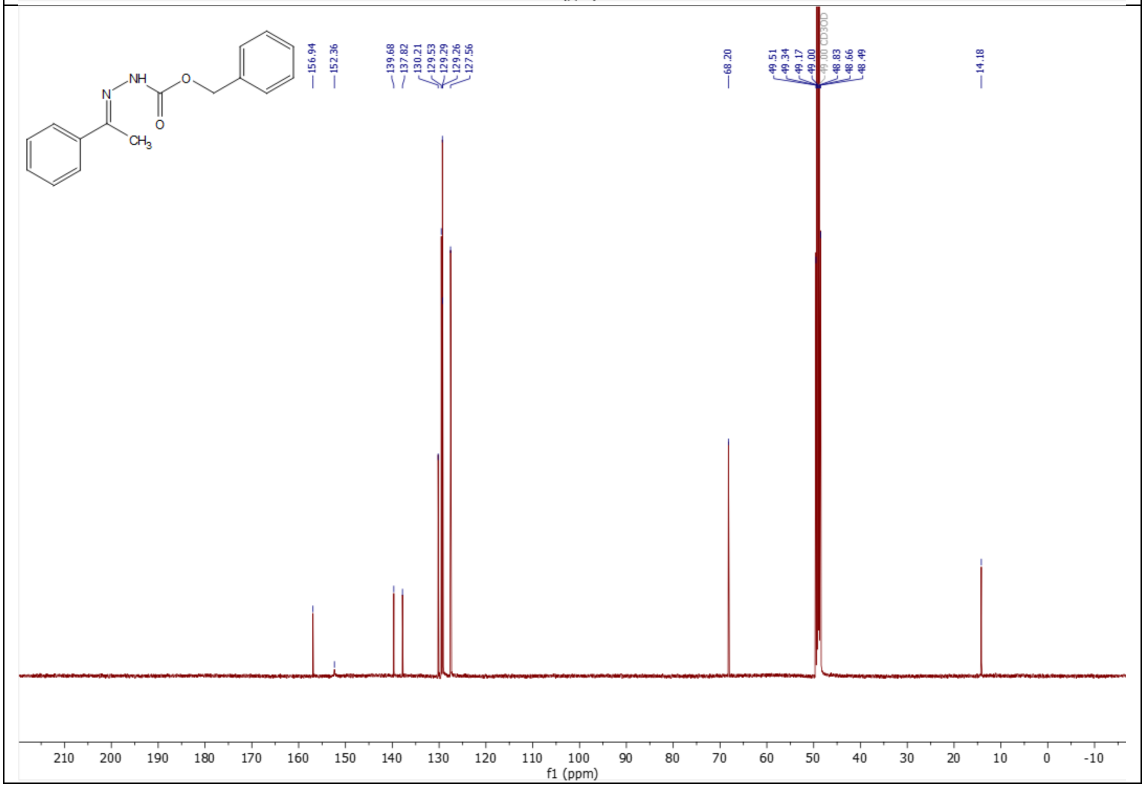

benzyl (*E*)-2-(1-(2-fluorophenyl)ethylidene)hydrazine-1-carboxylate (2)

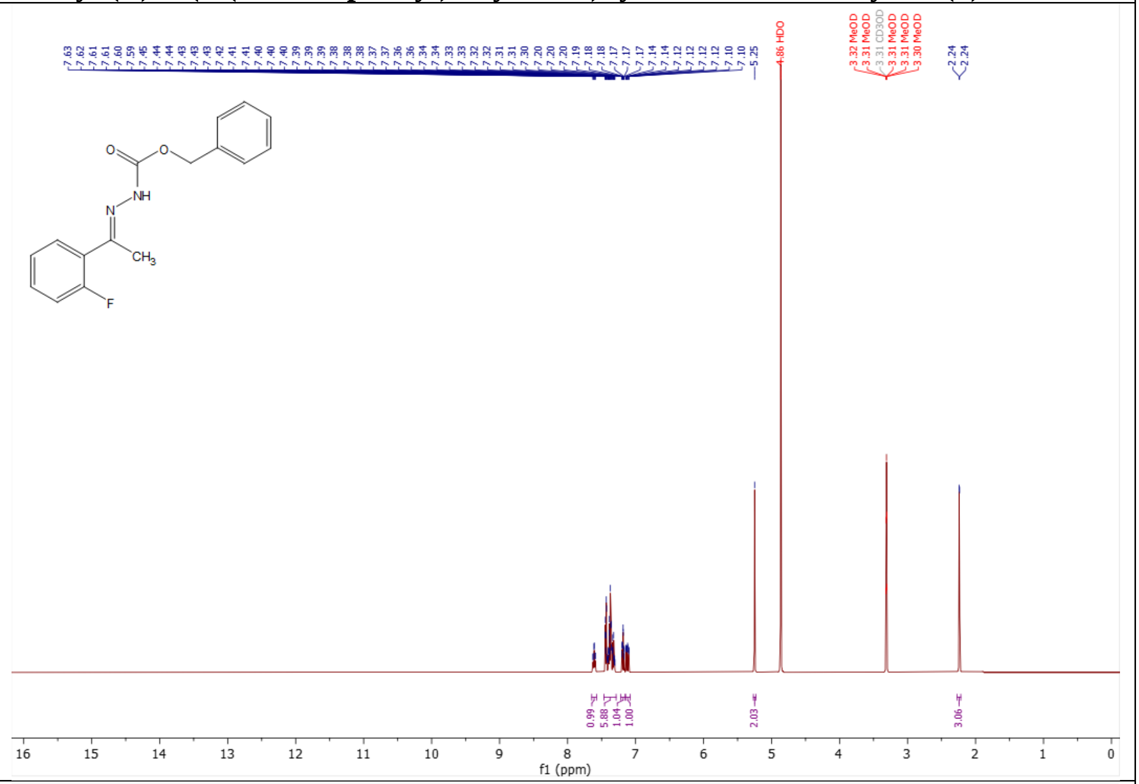

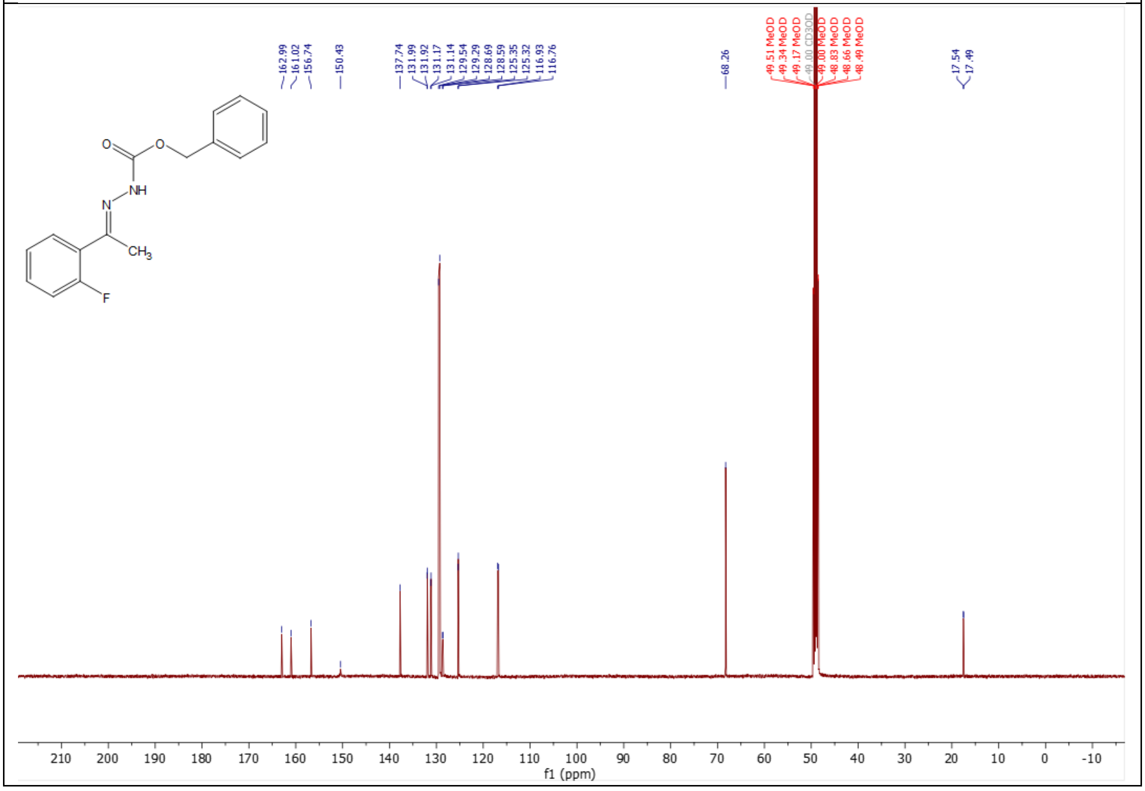

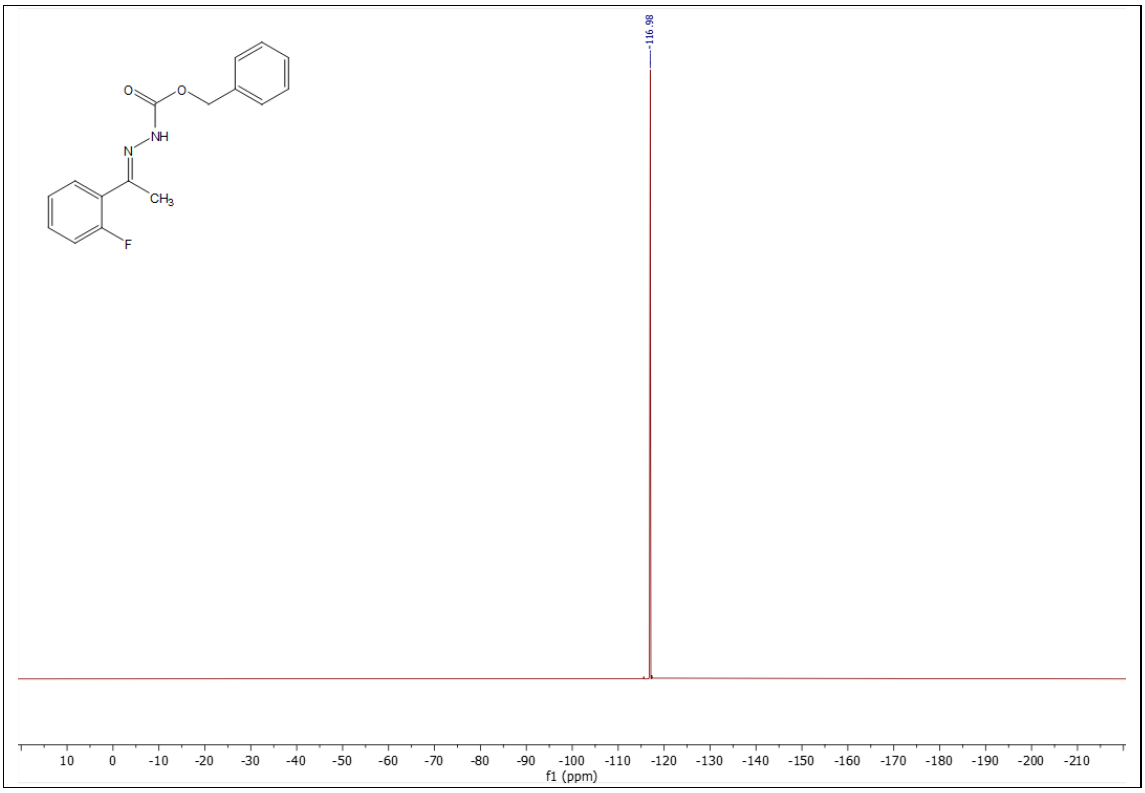
-116.98
O
O
NH
N
CH3
F
10 0 -10 -20 -30 -40 -50 -60 -70 -80 -90 -100 -110 -120 -130 -140 -150 -160 -170 -180 -190 -200 -210
f1 (ppm)

benzyl (*E*)-2-(1-(3-fluorophenyl)ethylidene)hydrazine-1-carboxylate (3)

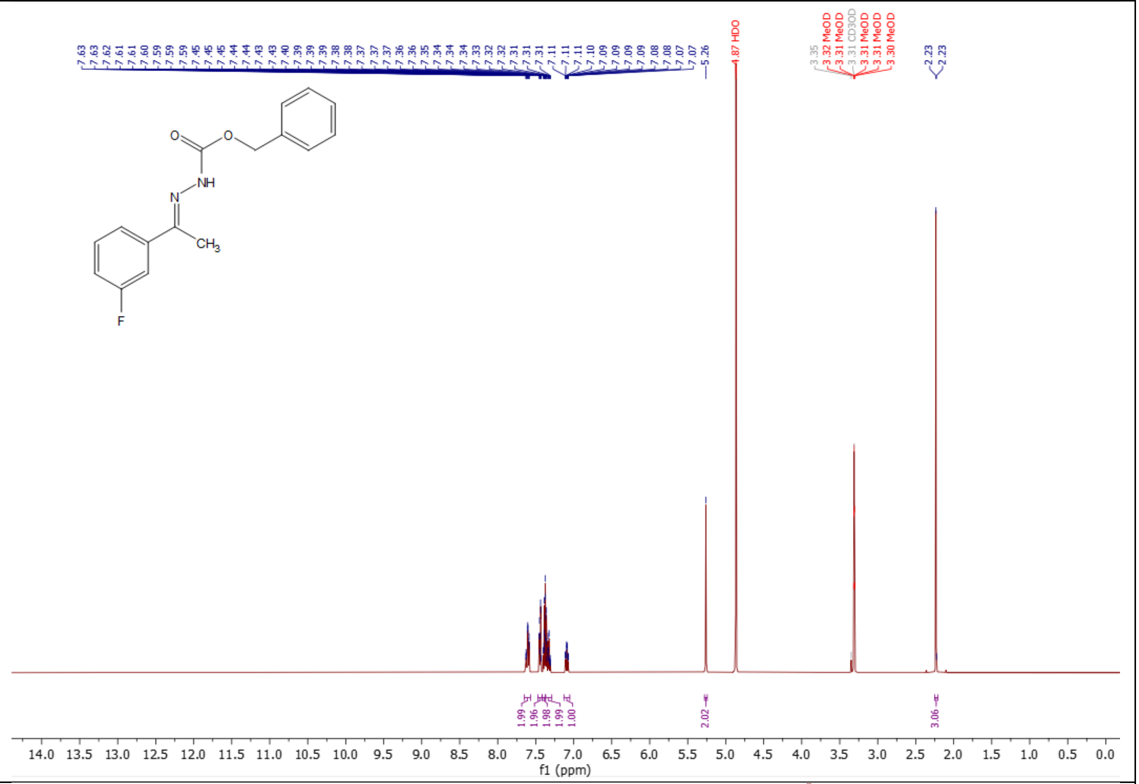

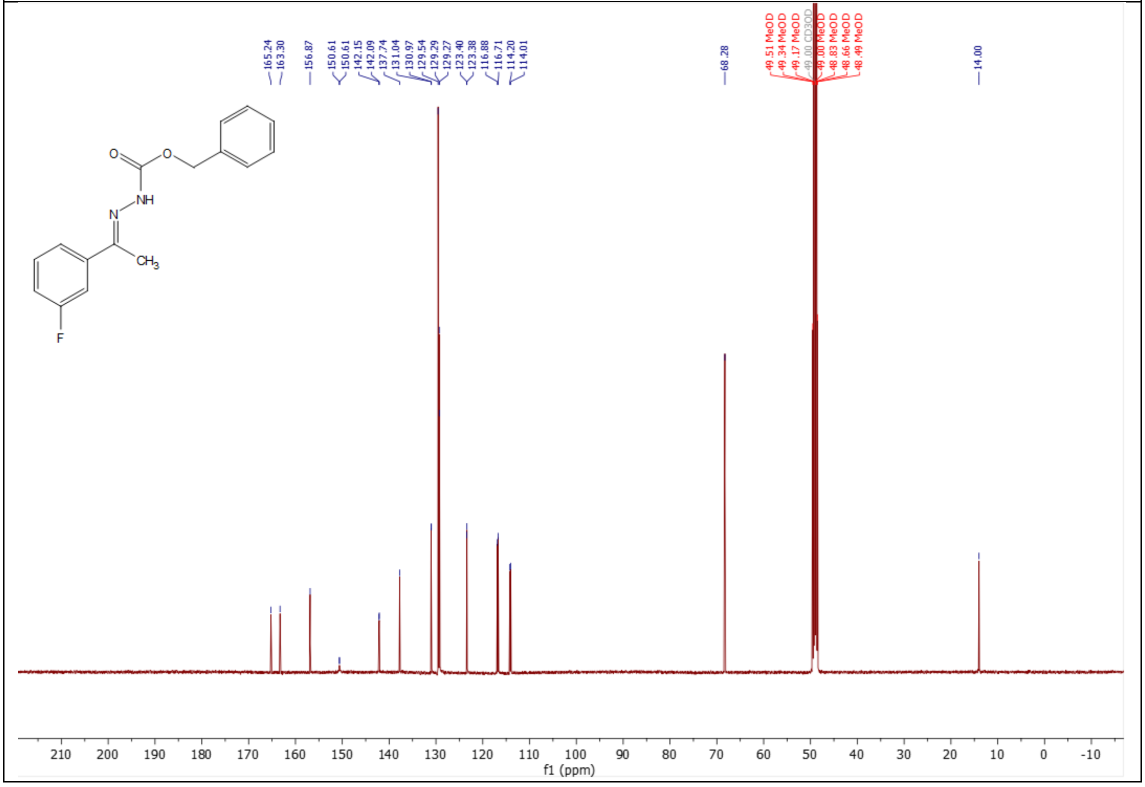

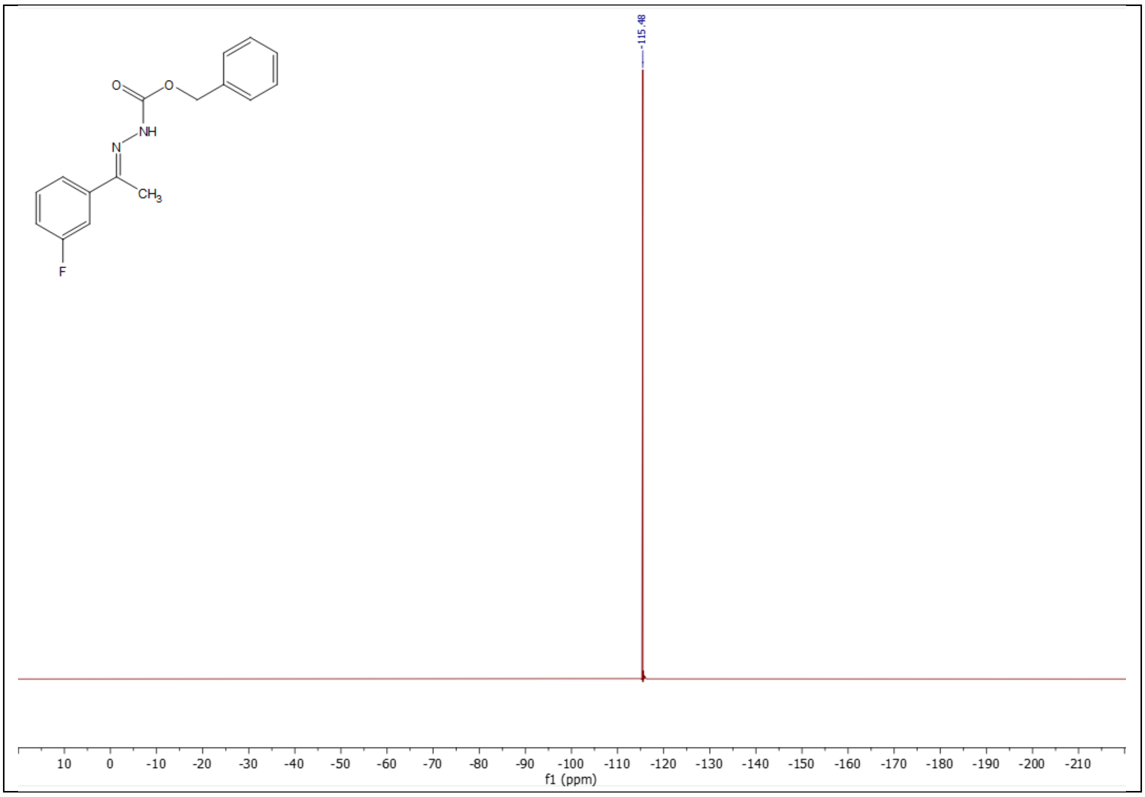

-115.48
O
O
NH
N
CH3
F
10
0
-10
-20
-30
-40
-50
-60
-70
-80
-90
-100
-110
-120
-130
-140
-150
-160
-170
-180
-190
-200
-210
f1 (ppm)

benzyl (*E*)-2-(1-(4-fluorophenyl)ethylidene)hydrazine-1-carboxylate (4)

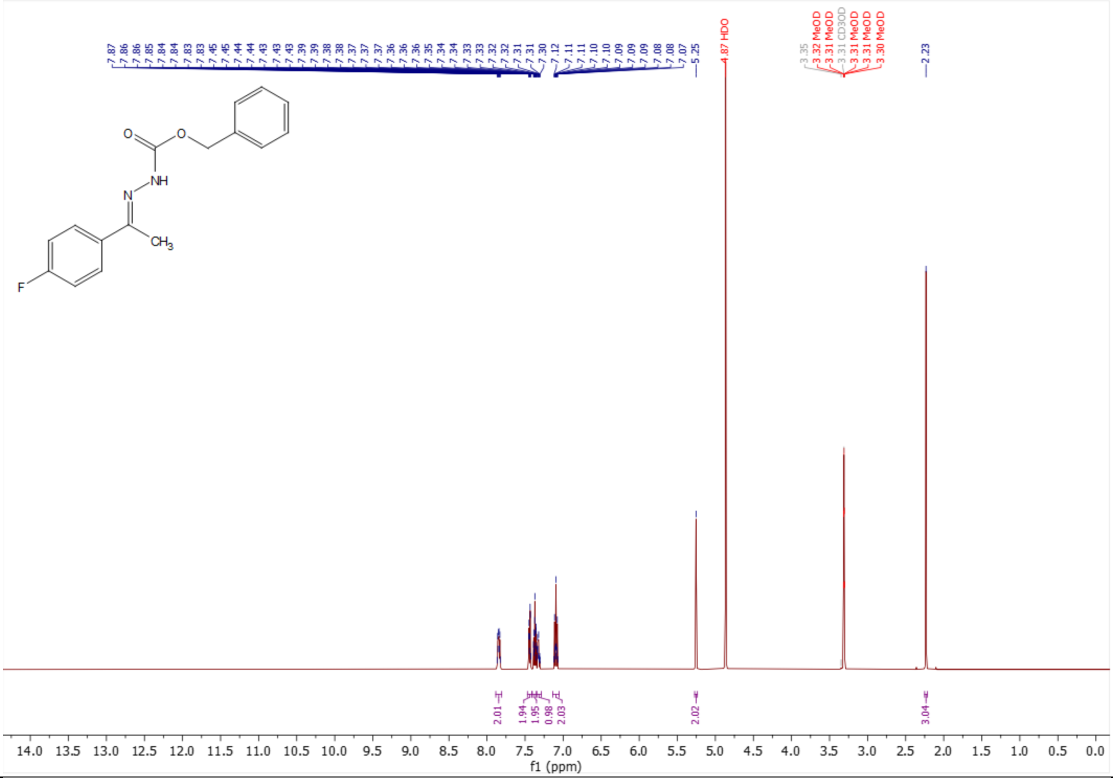

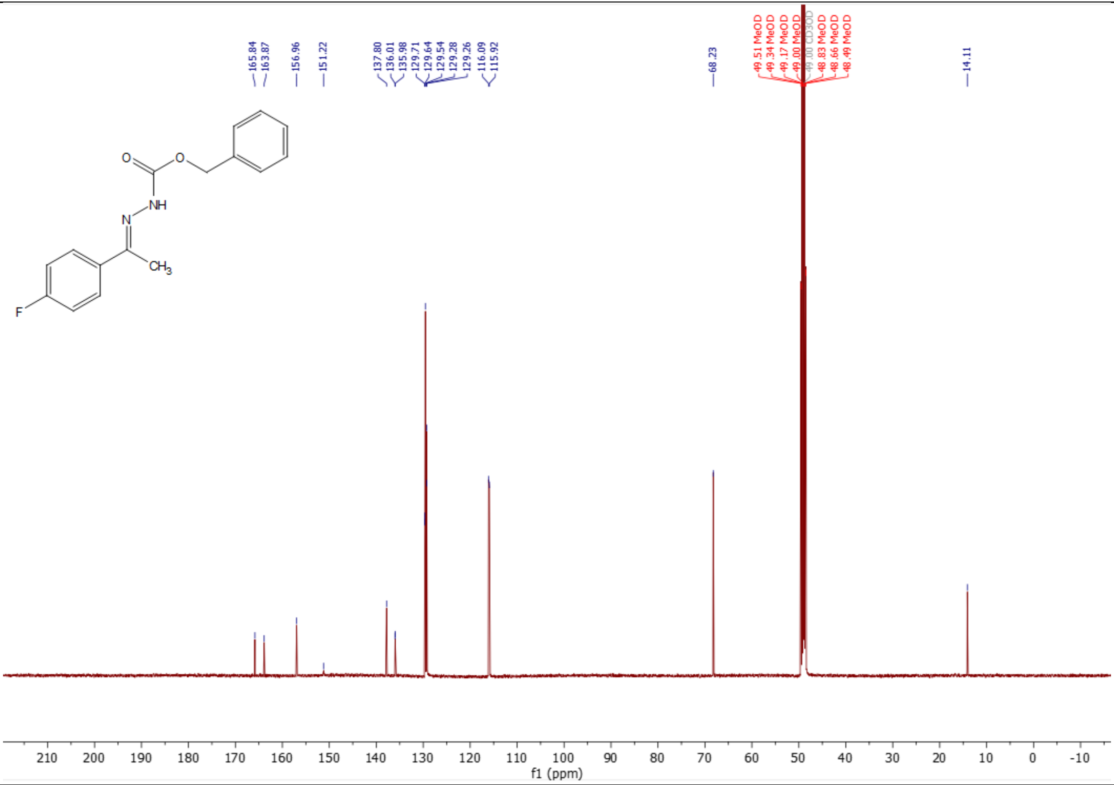

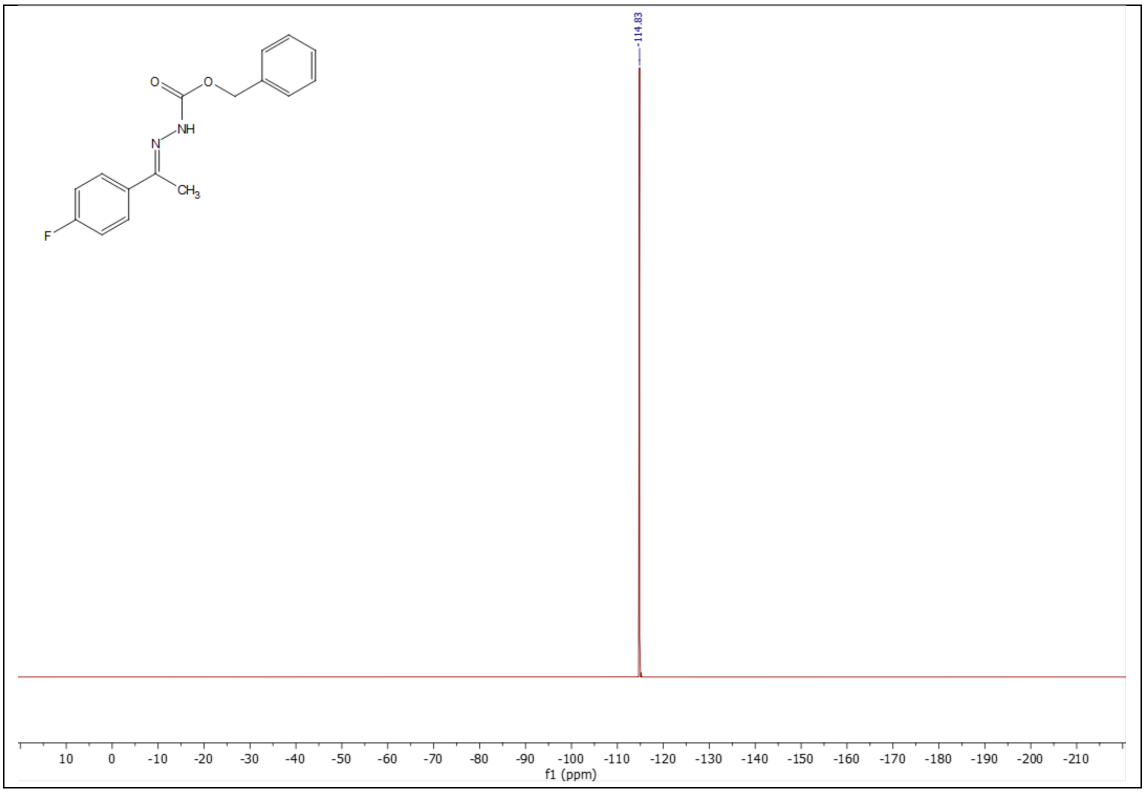

O
O
NH
N
CH3
F
-114.83
10
0
-10
-20
-30
-40
-50
-60
-70
-80
-90
-100
-110
-120
-130
-140
-150
-160
-170
-180
-190
-200
-210
f1 (ppm)

benzyl (*E*)-2-(1-(pyridin-3-yl)ethylidene)hydrazine-1-carboxylate (5)

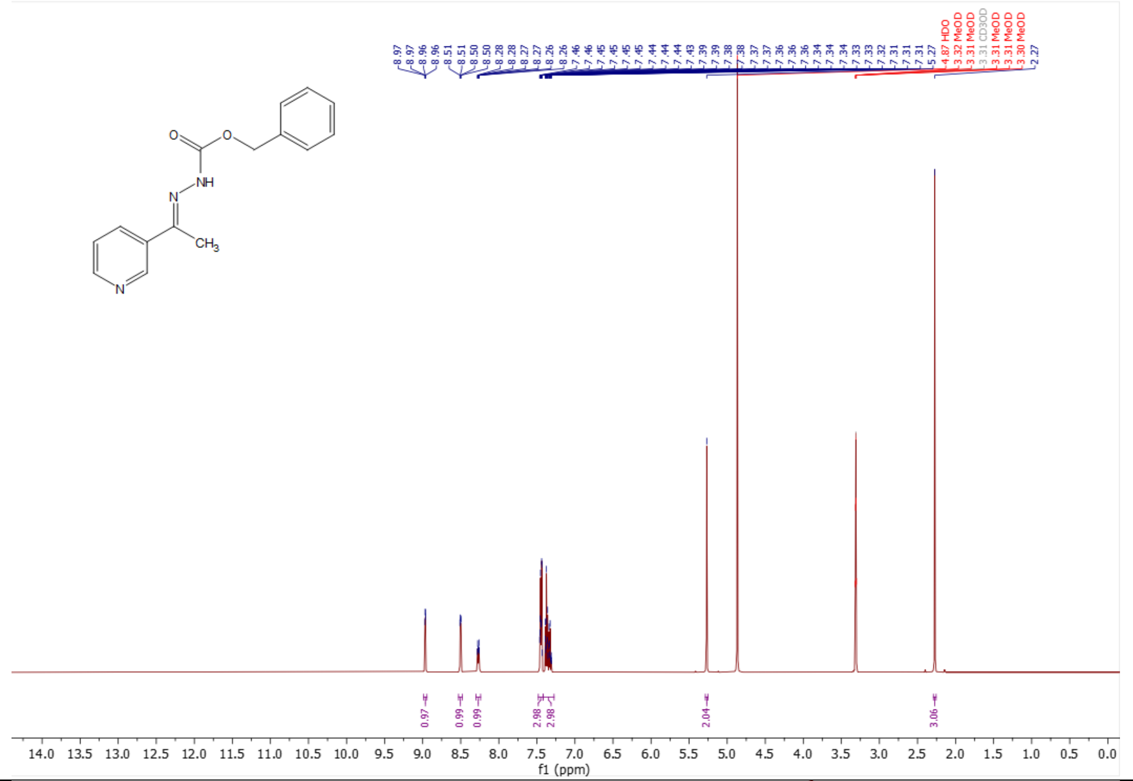

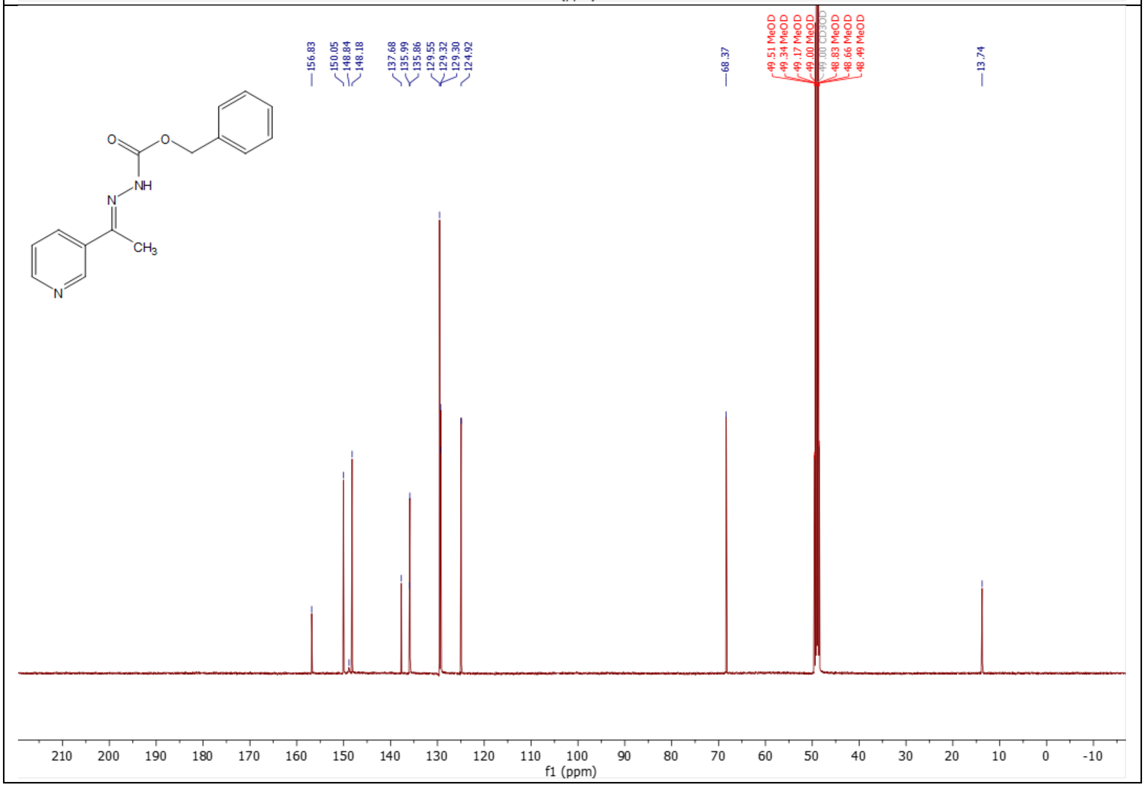

benzyl (*E*)-2-(1-(pyridin-4-yl)ethylidene)hydrazine-1-carboxylate (6)

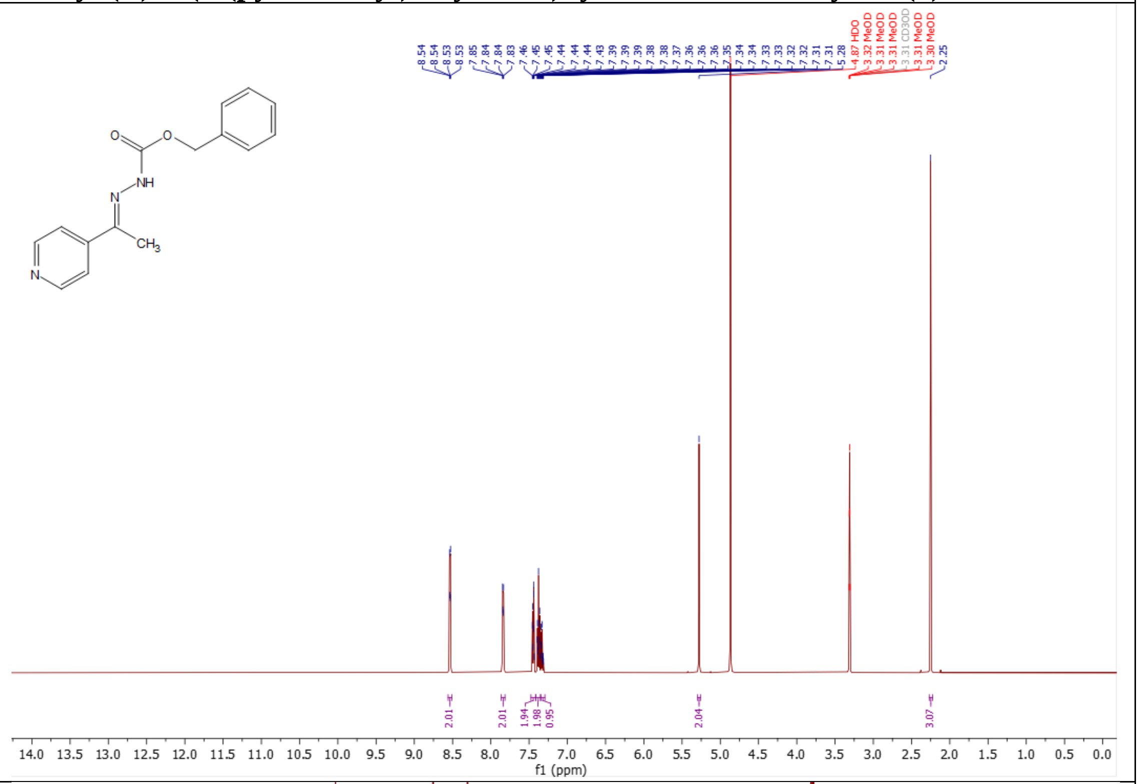

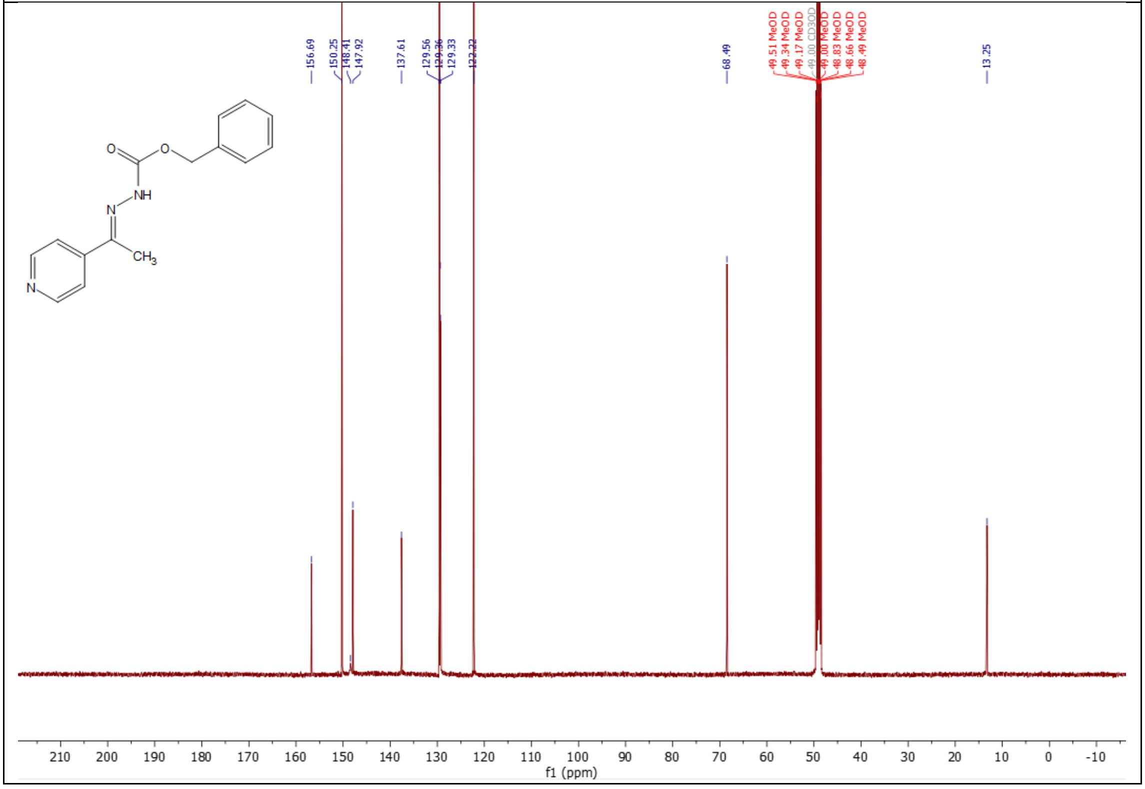

benzyl (*E*)-2-(pentan-2-ylidene)hydrazine-1-carboxylate (7)

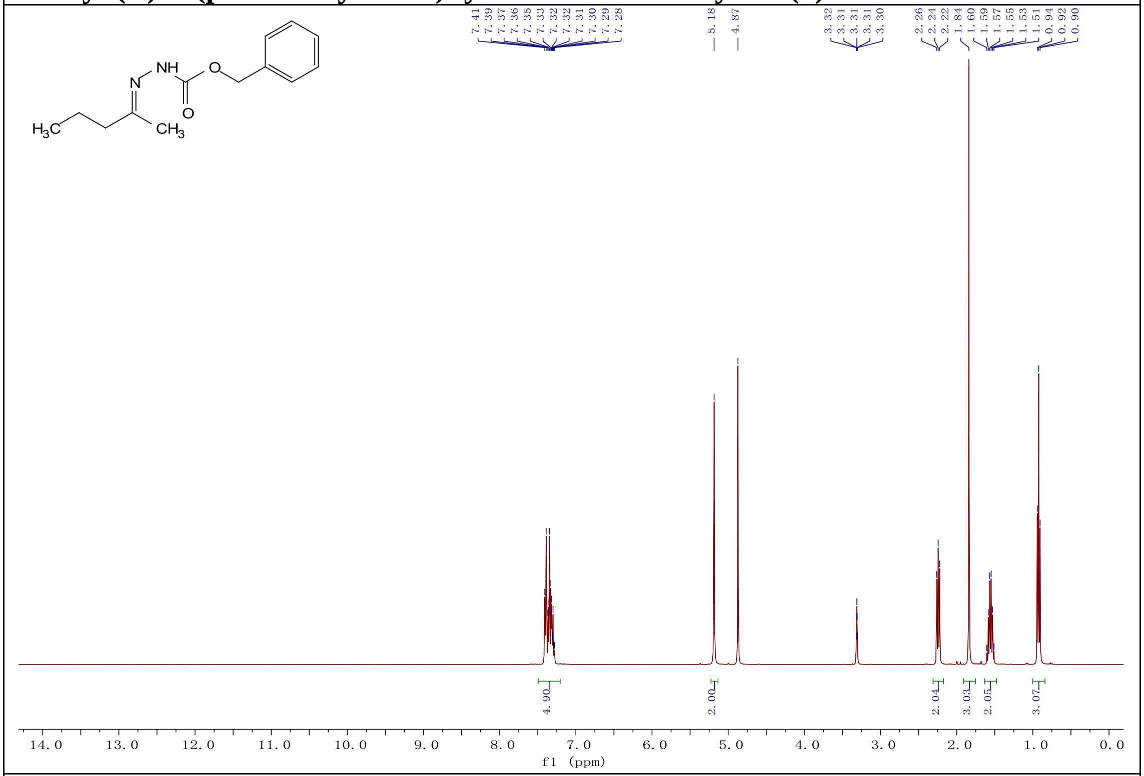

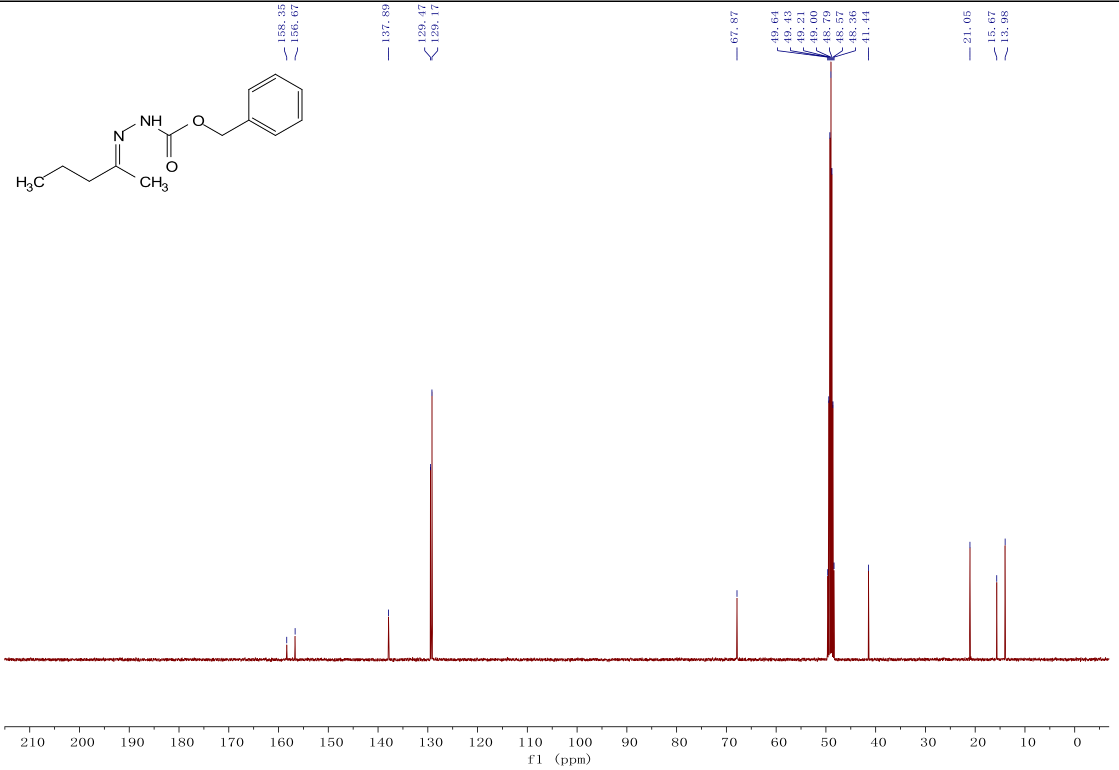

benzyl (*E*)-2-(3,3-dimethylbutan-2-ylidene)hydrazine-1-carboxylate (8)

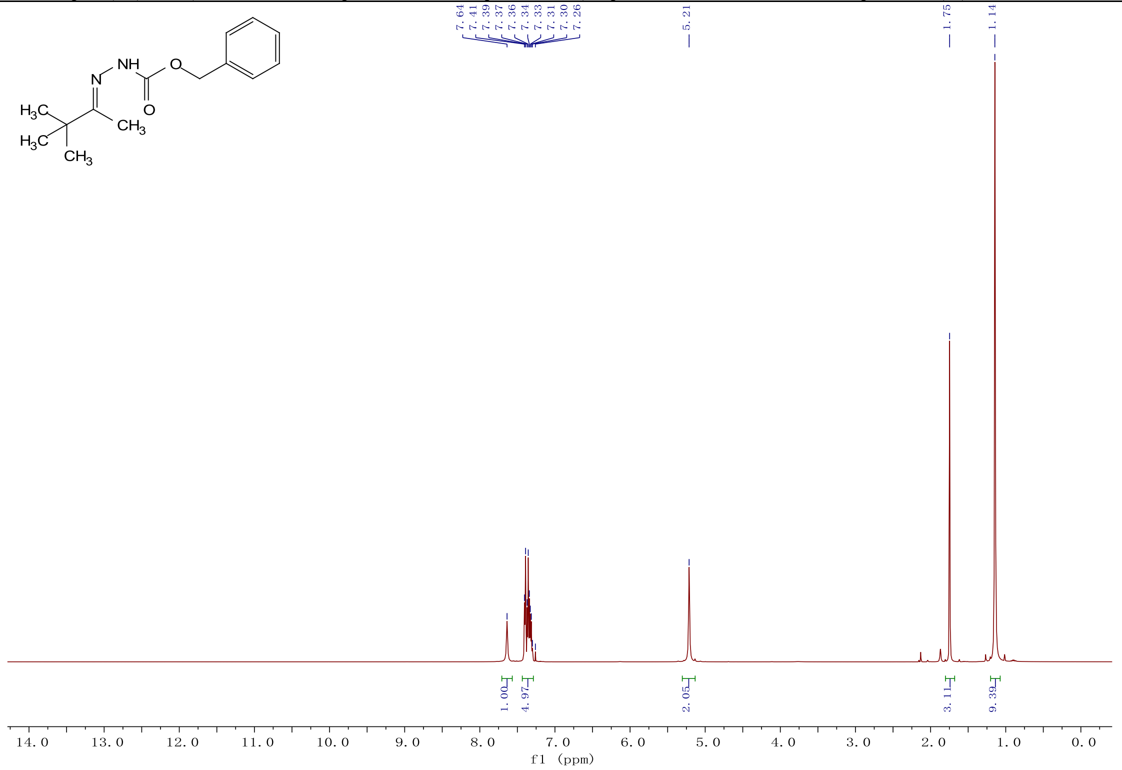

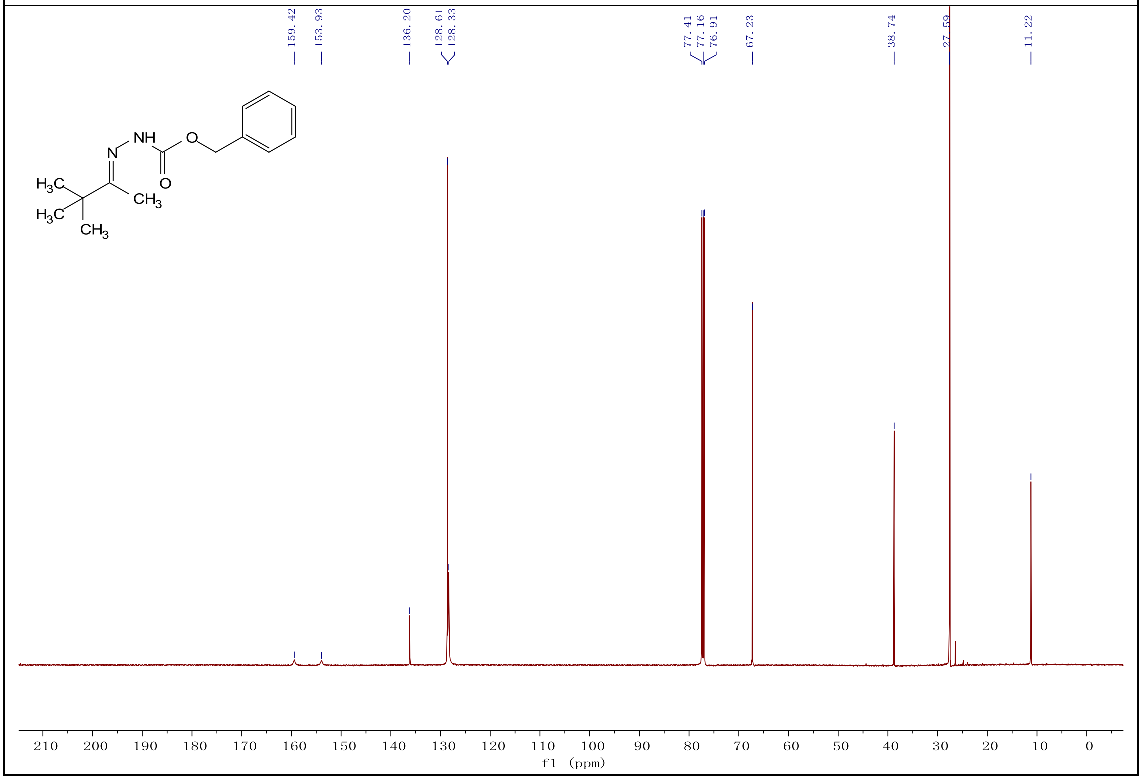

(*E*)-*N'*-(1-phenylethylidene)benzohydrazide (9)

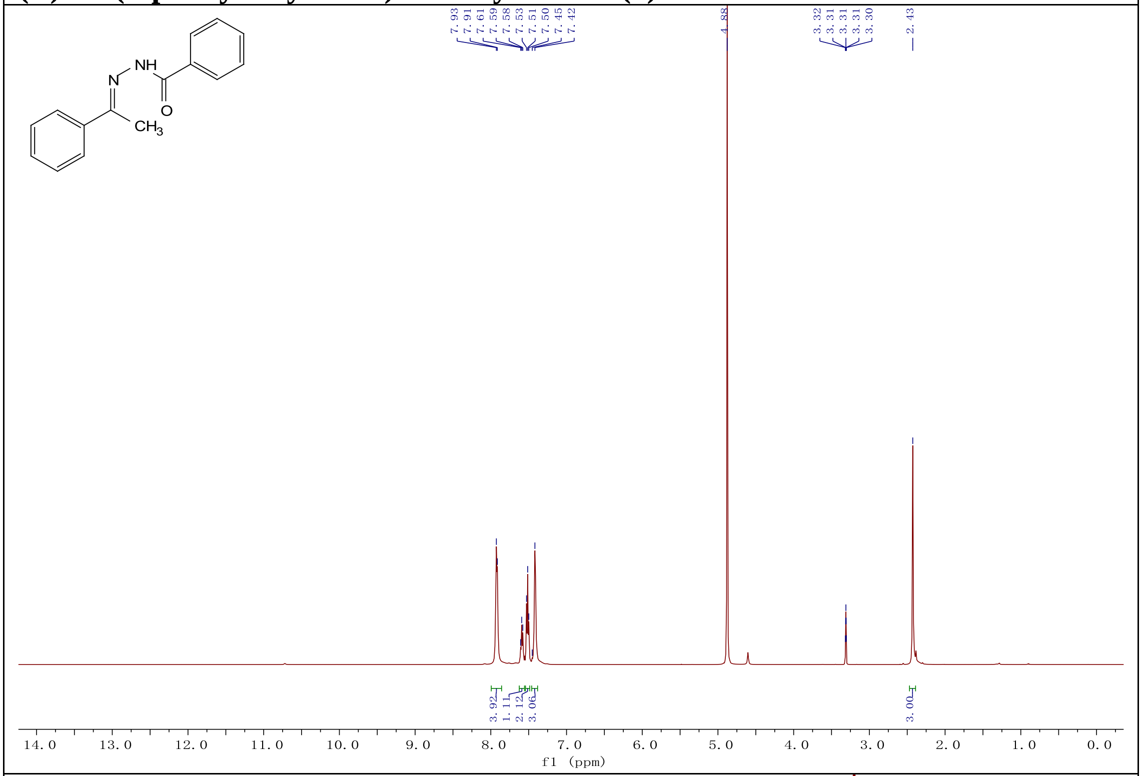

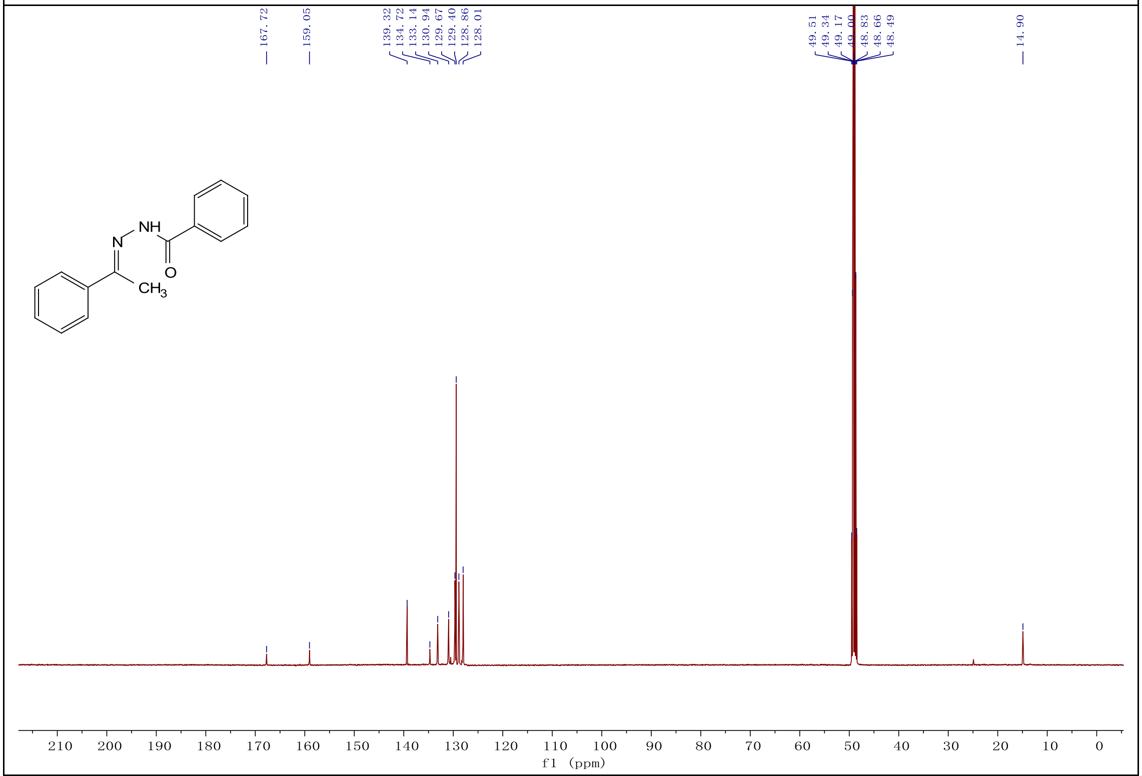

(*E*)-*N'*-(pentan-2-ylidene)benzohydrazide (10)

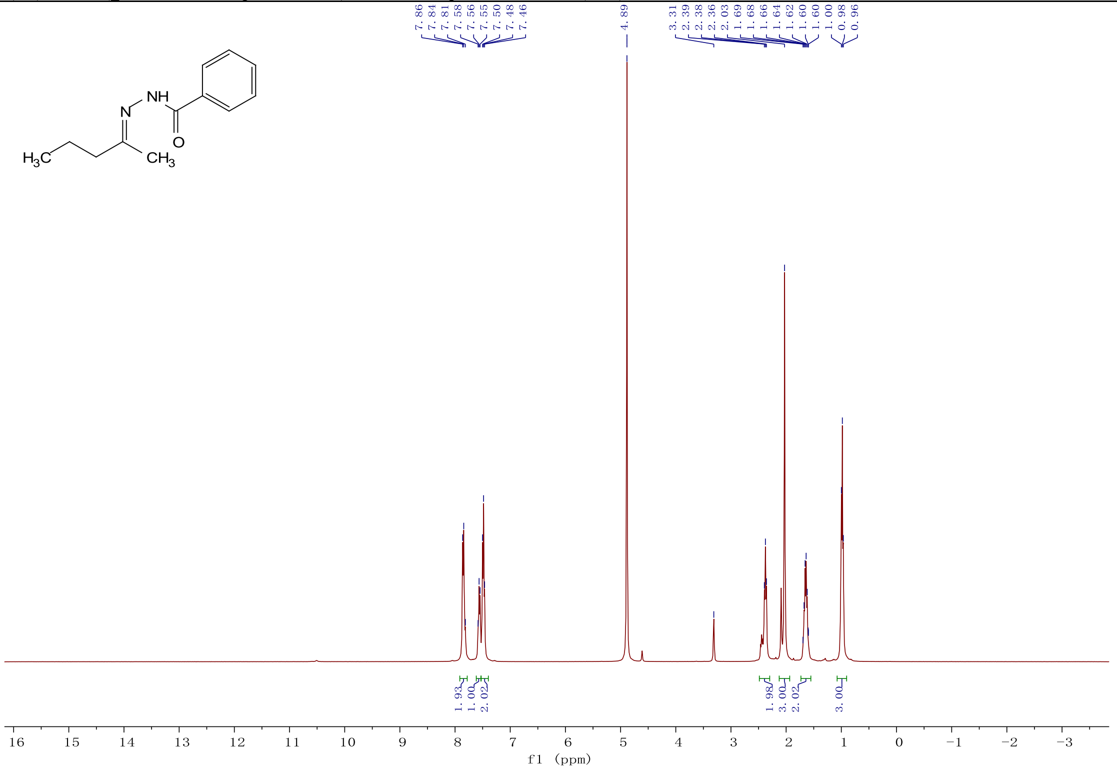

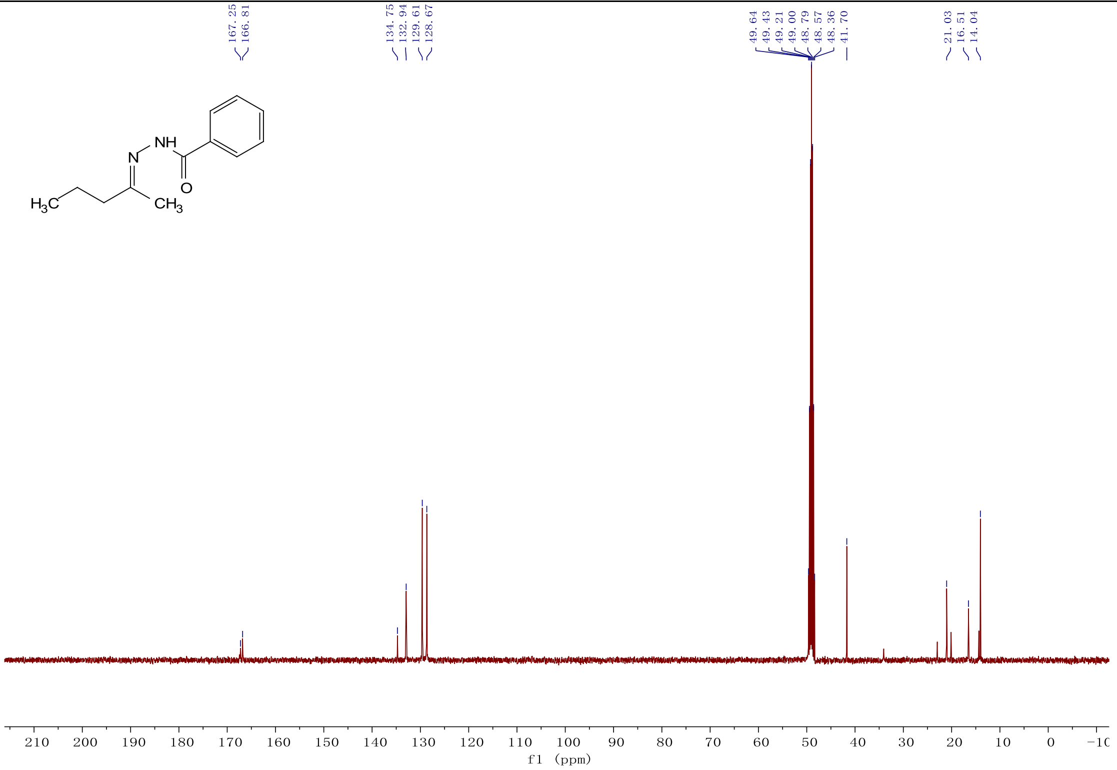

tert-butyl (_E_)-2-(3,3-dimethylbutan-2-ylidene)hydrazine-1-carboxylate (11)

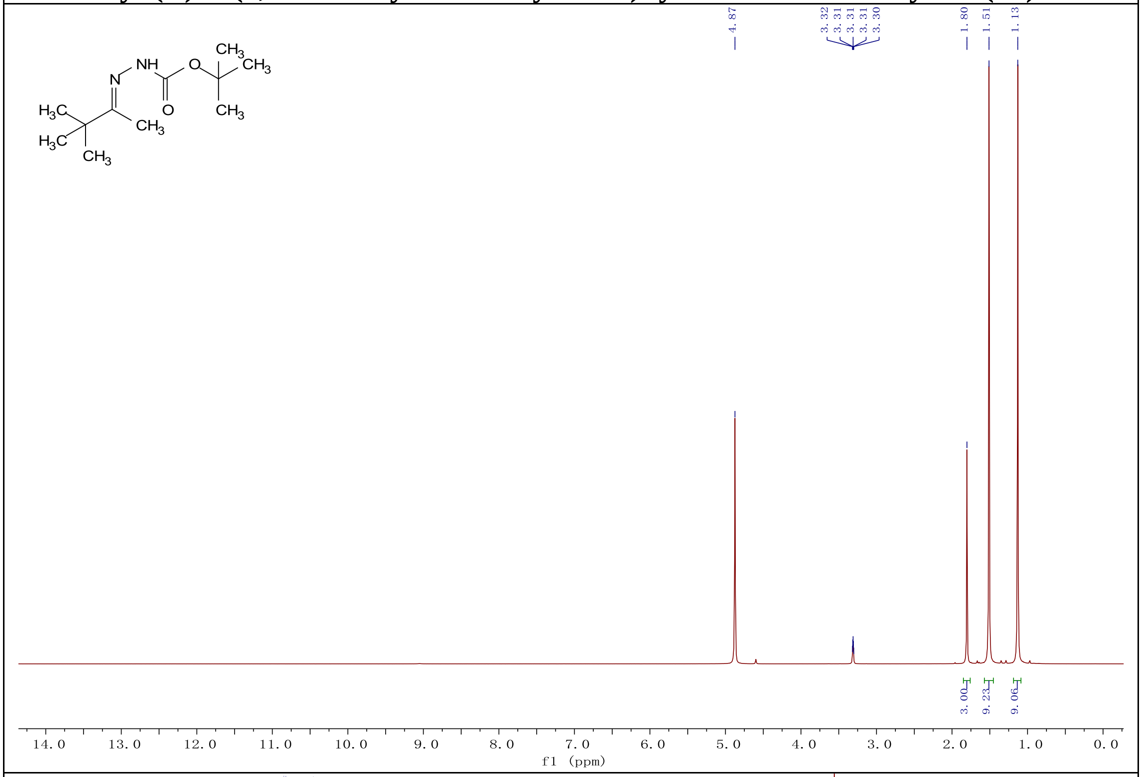

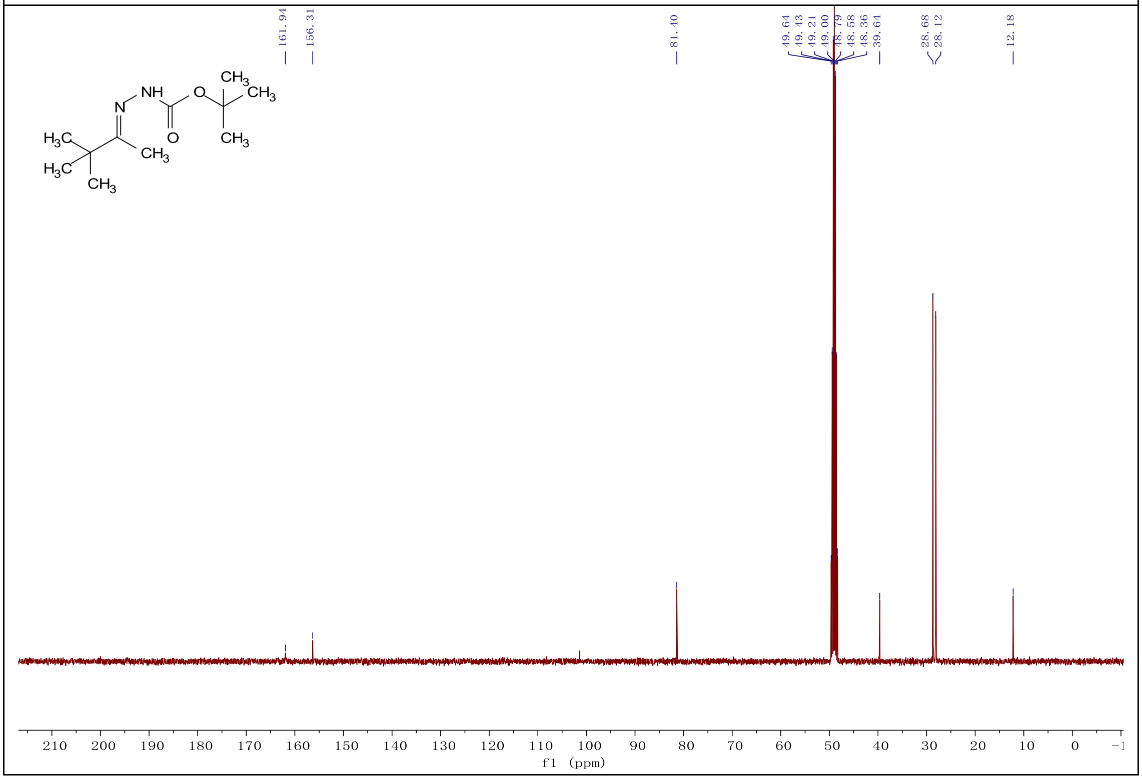

benzyl 2-(1-phenylethyl)hydrazine-1-carboxylate (1a)

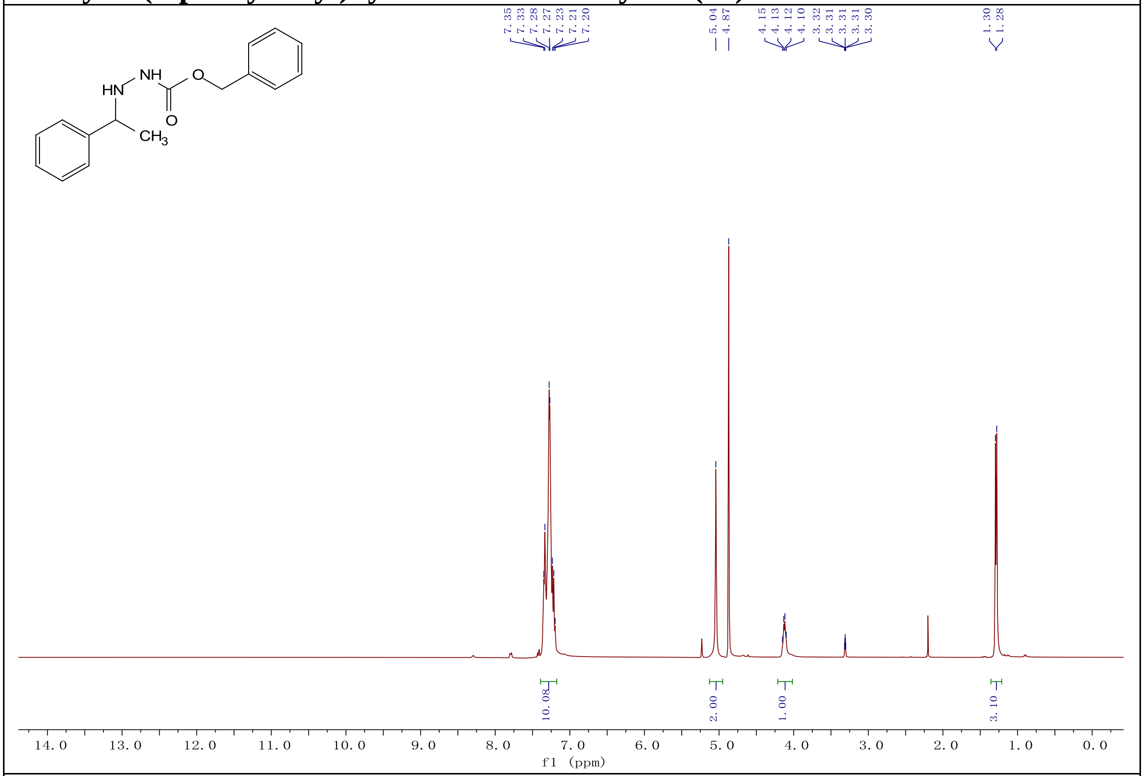

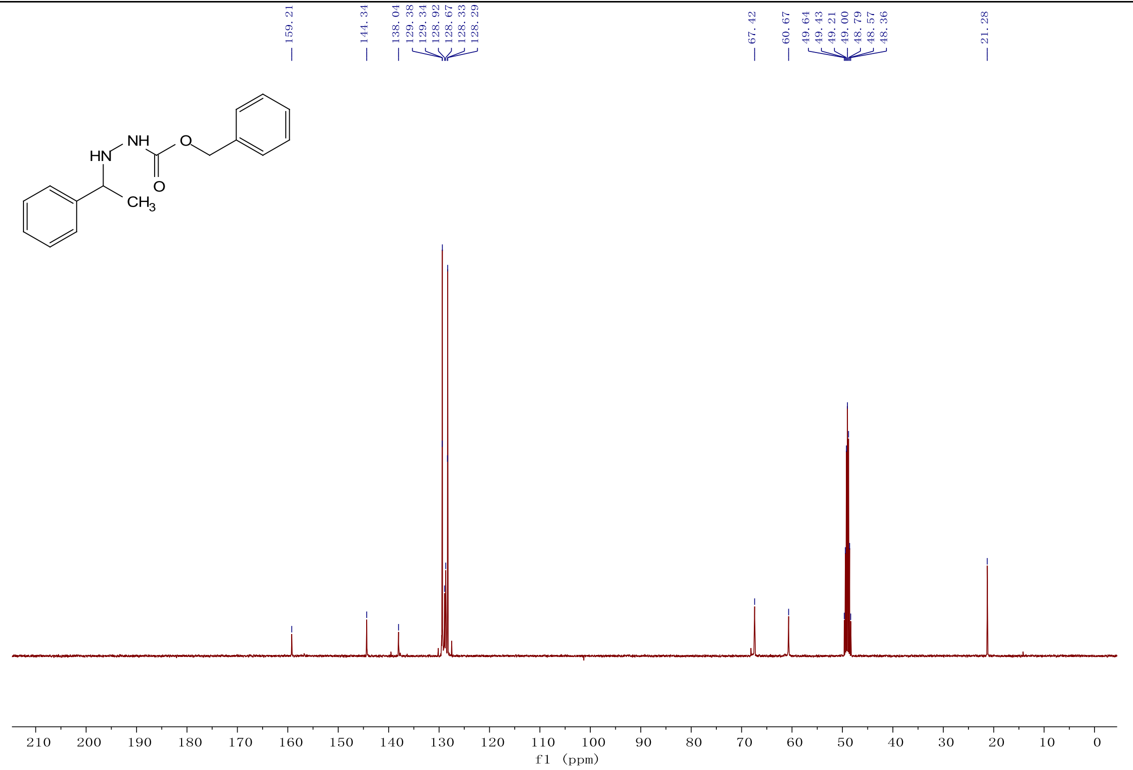

benzyl 2-(1-(2-fluorophenyl)ethyl)hydrazine-1-carboxylate (2a)

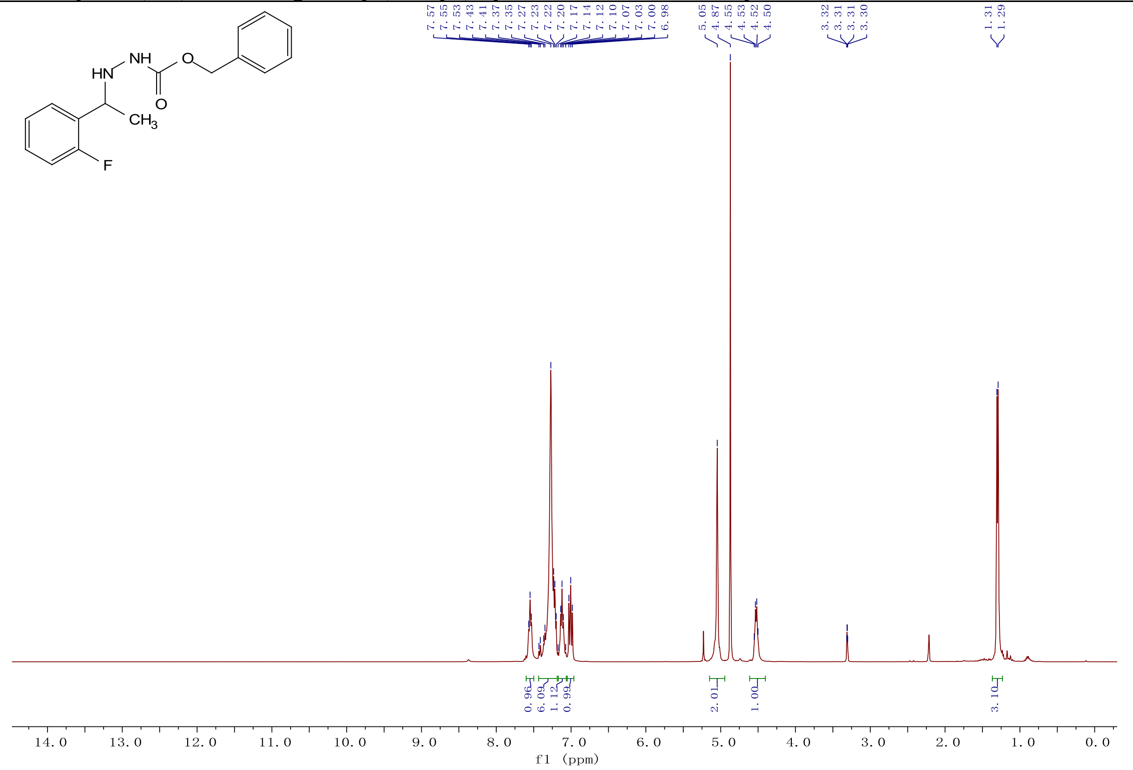

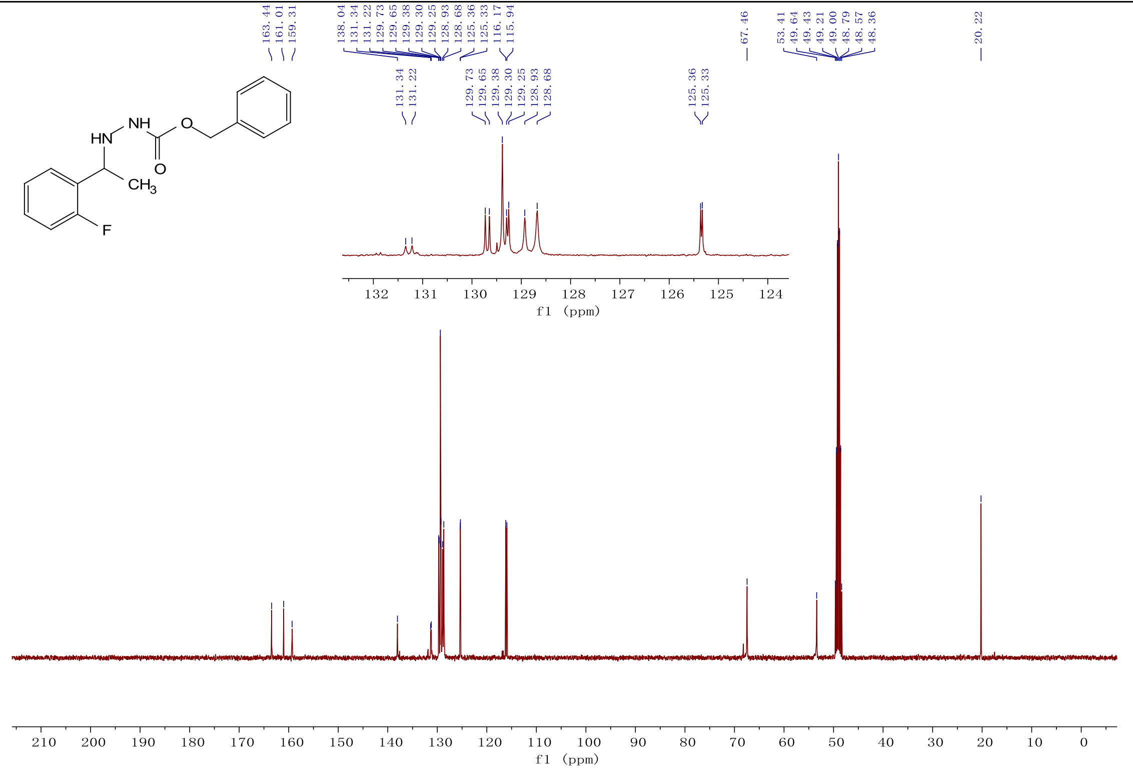

benzyl 2-(1-(3-fluorophenyl)ethyl)hydrazine-1-carboxylate (3a)

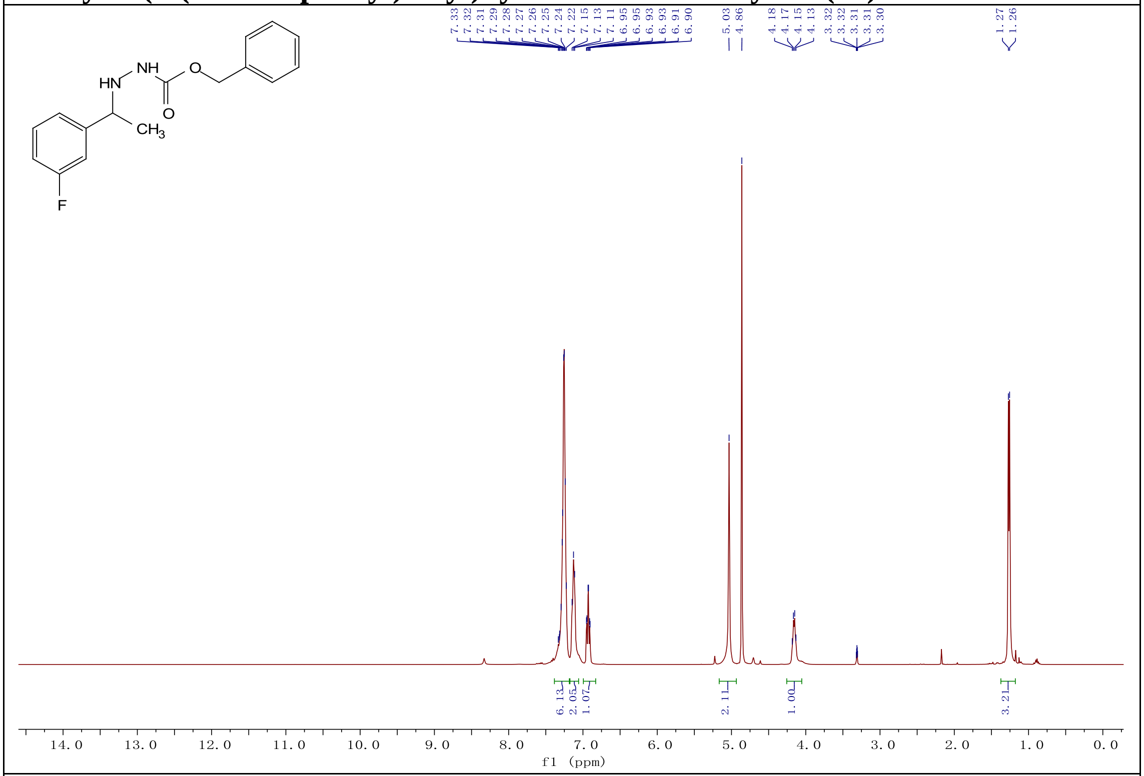

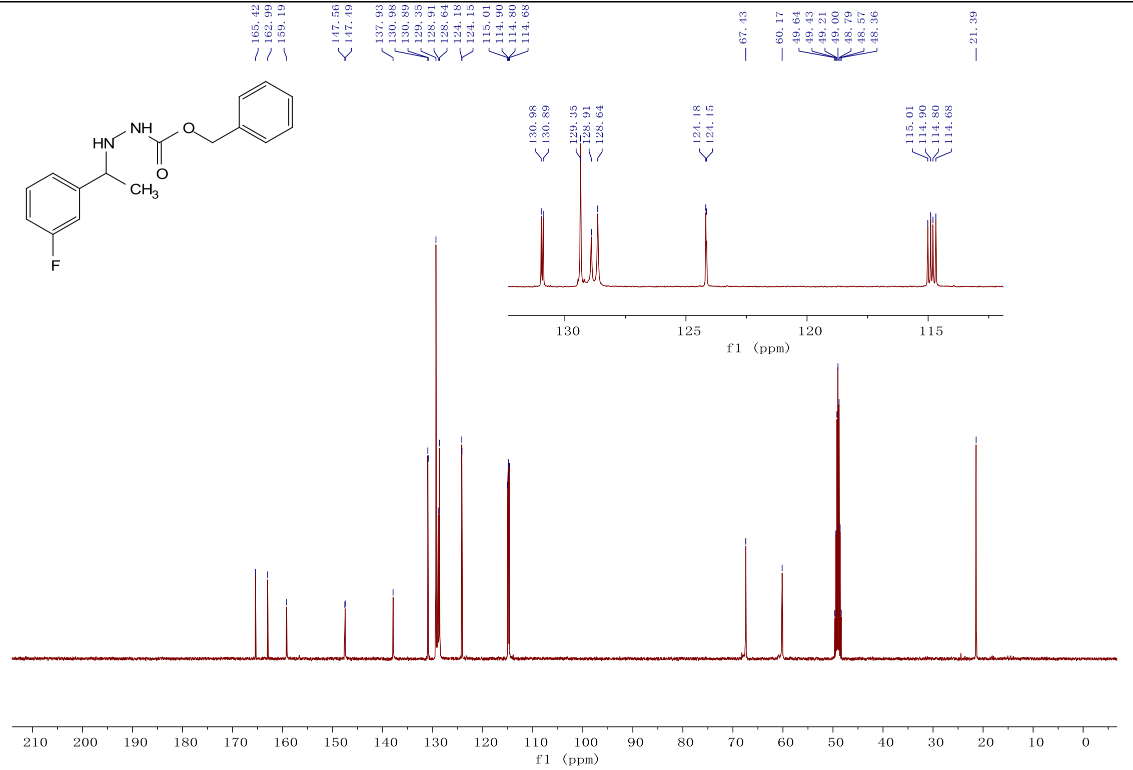

benzyl 2-(1-(4-fluorophenyl)ethyl)hydrazine-1-carboxylate (4a)

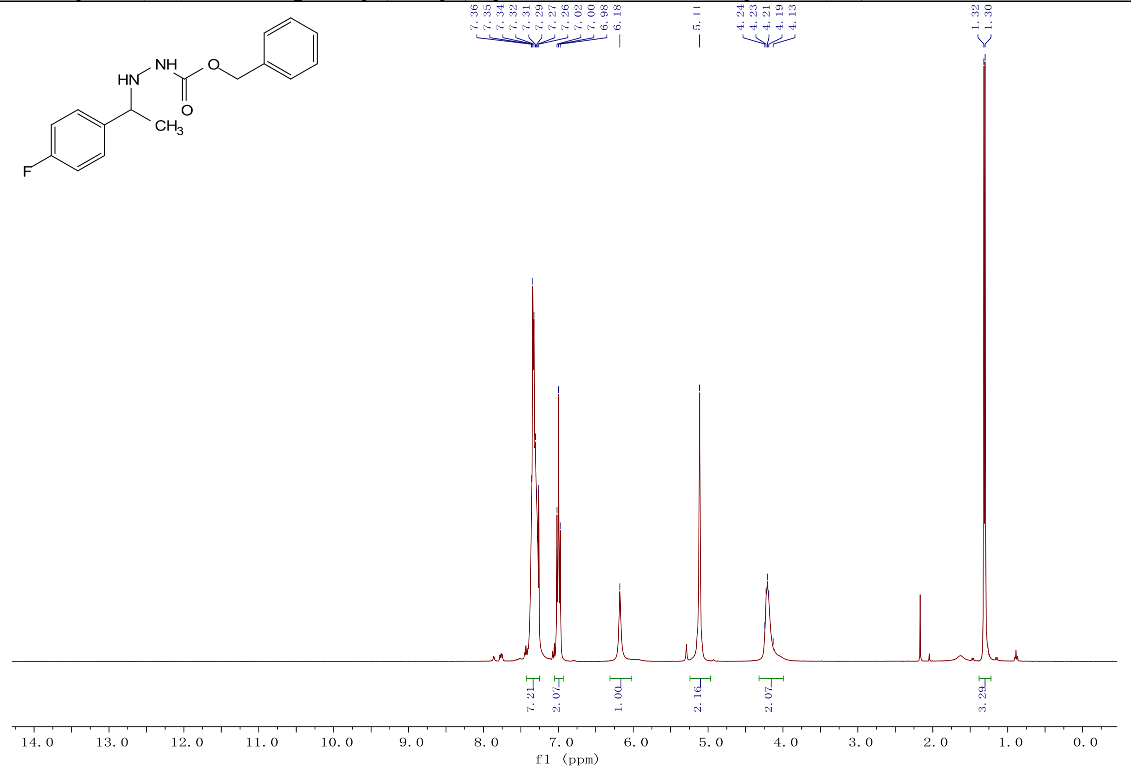

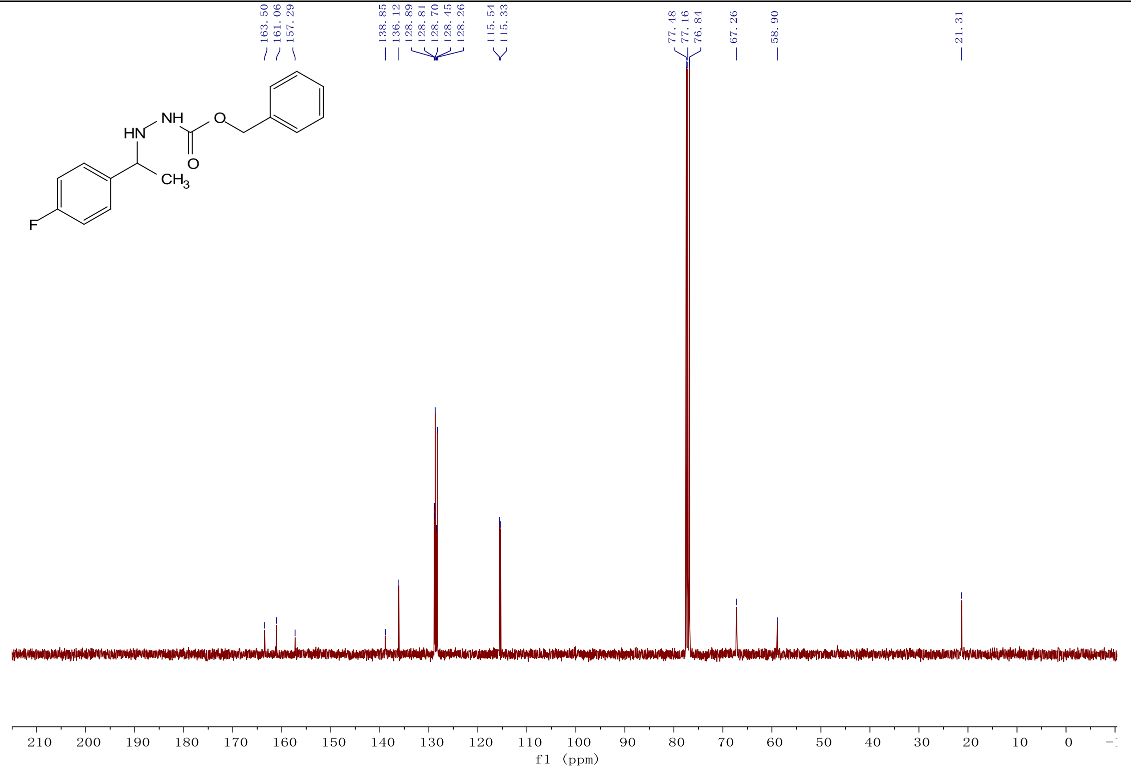

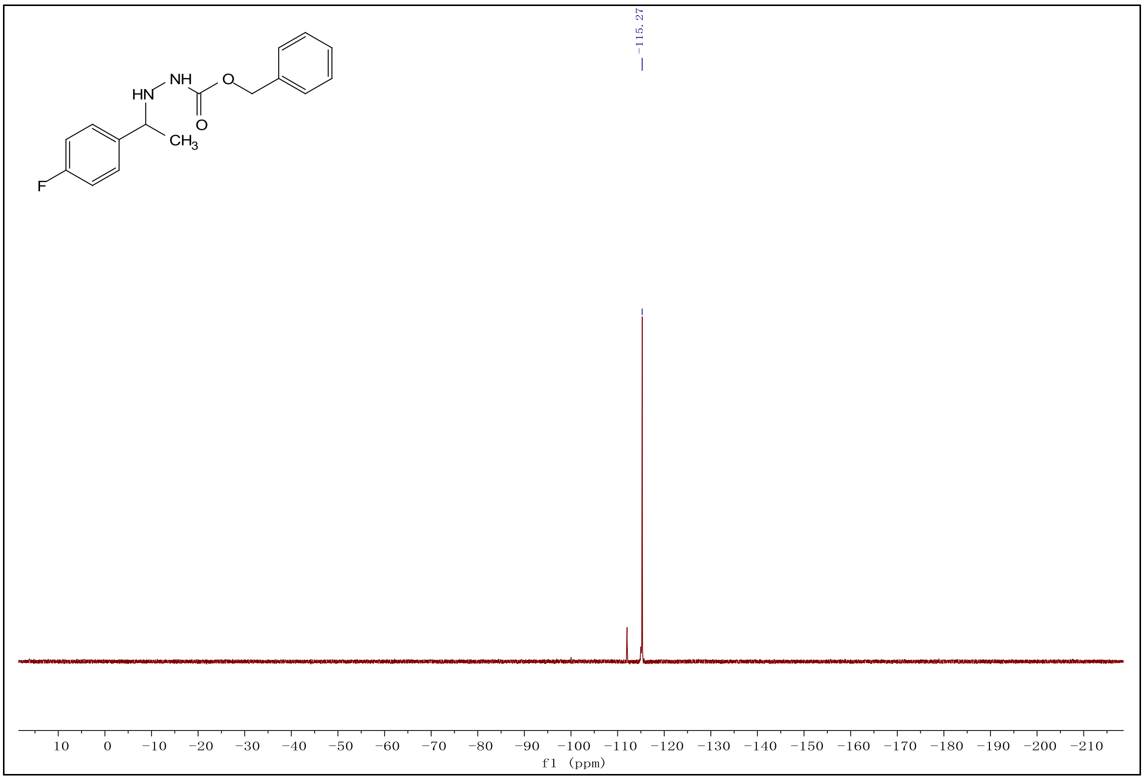

-115.27
HN
NH
O
O
CH3
F
10 0 -10 -20 -30 -40 -50 -60 -70 -80 -90 -100 -110 -120 -130 -140 -150 -160 -170 -180 -190 -200 -210
f1 (ppm)

benzyl 2-(1-(pyridin-3-yl)ethyl)hydrazine-1-carboxylate (5a)

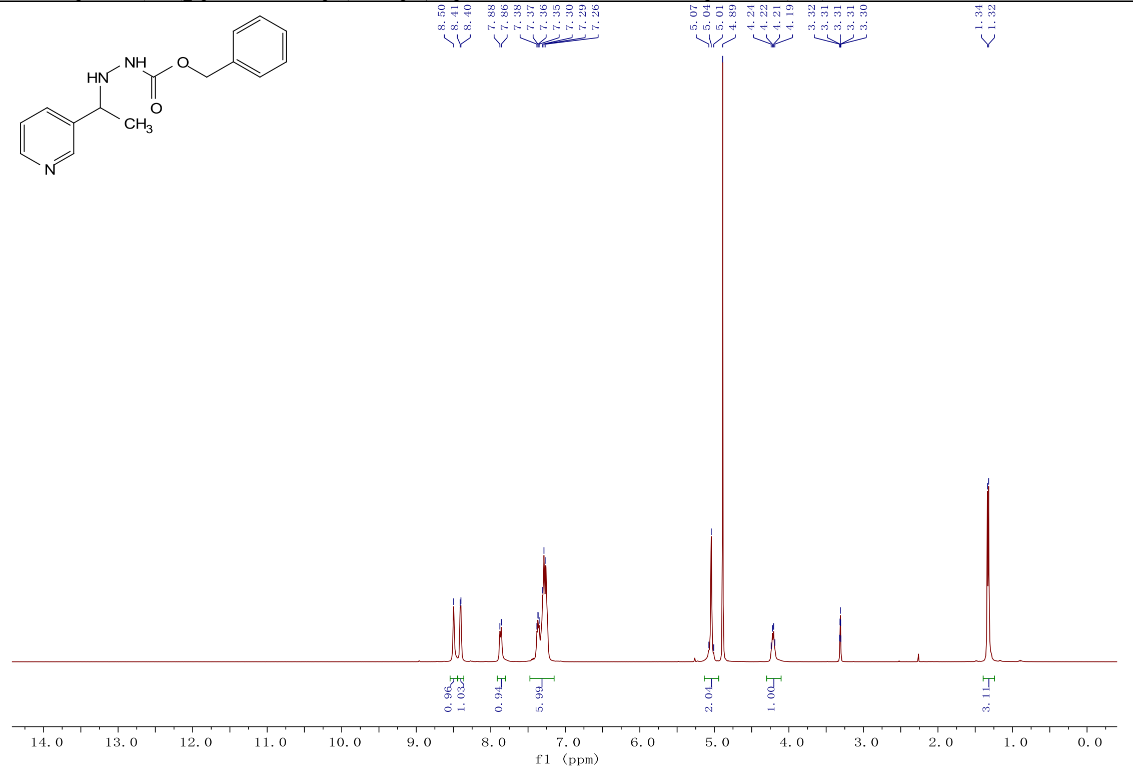

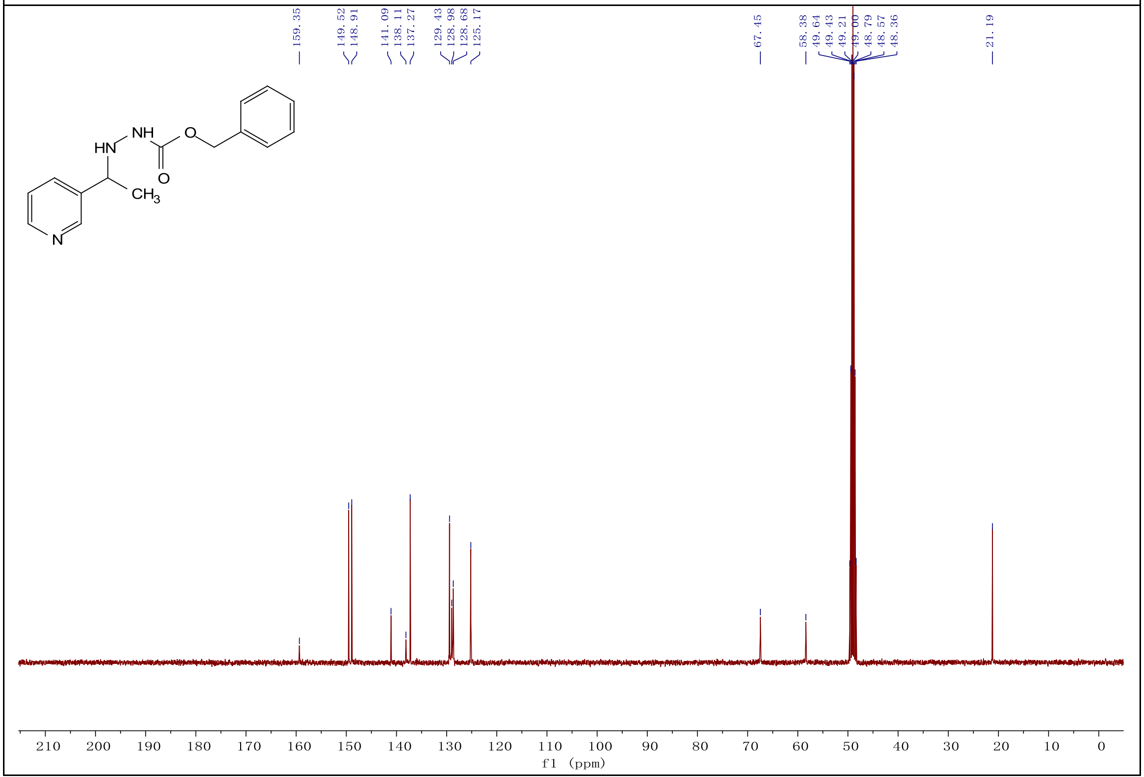

benzyl 2-(1-(pyridin-4-yl)ethyl)hydrazine-1-carboxylate (6a)

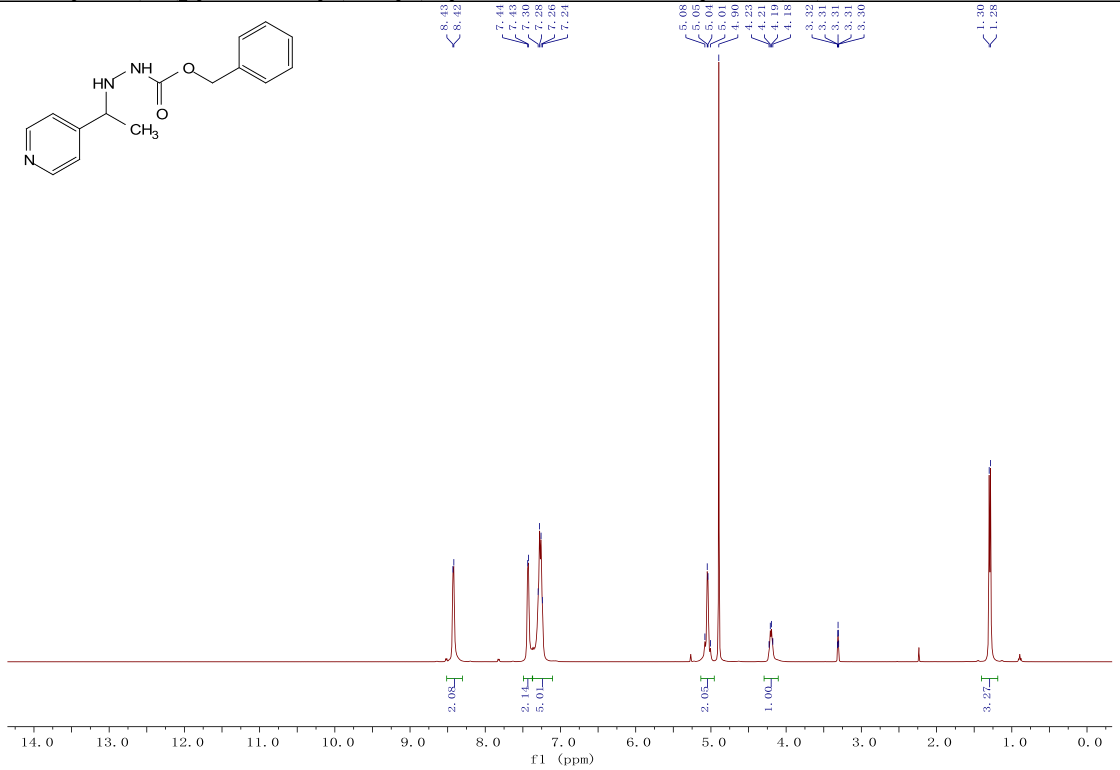

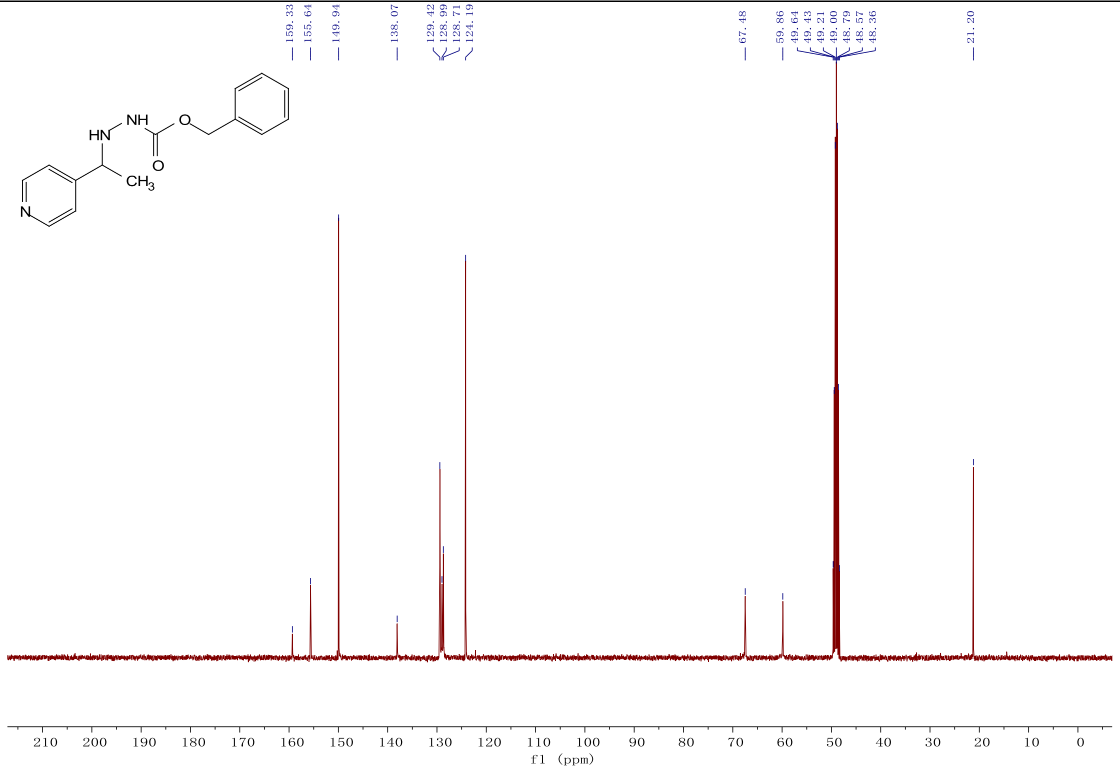

benzyl 2-(pentan-2-yl)hydrazine-1-carboxylate (7a)

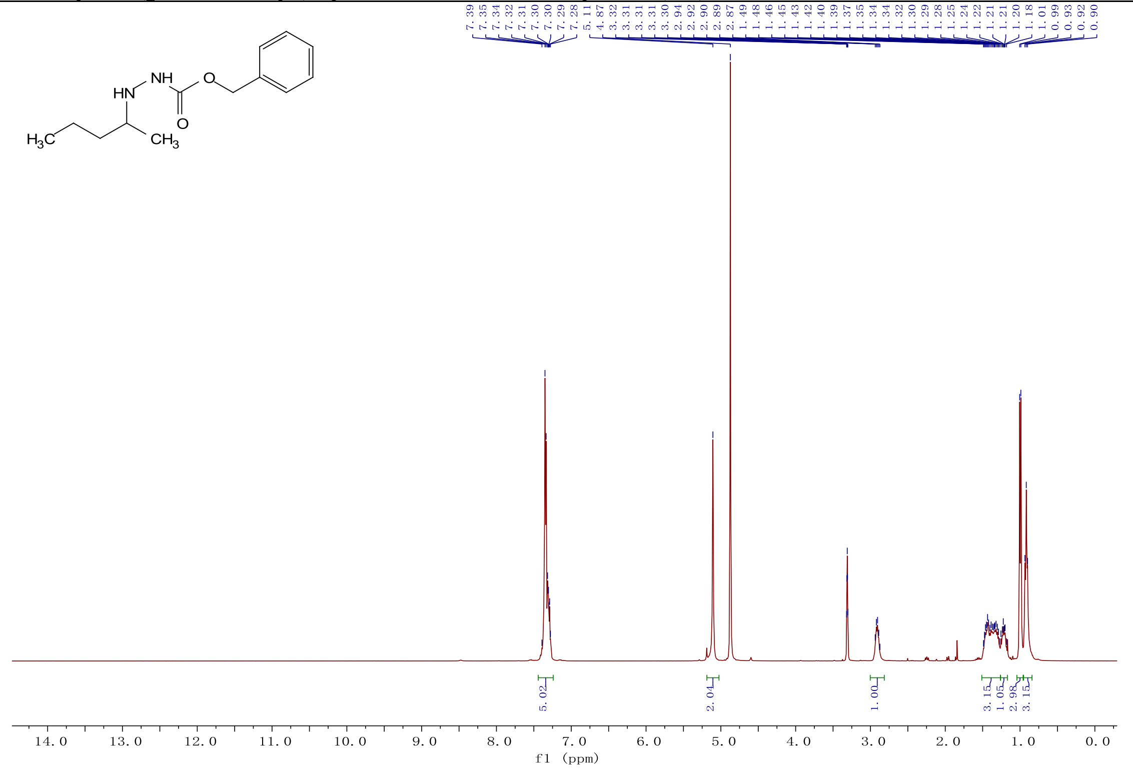

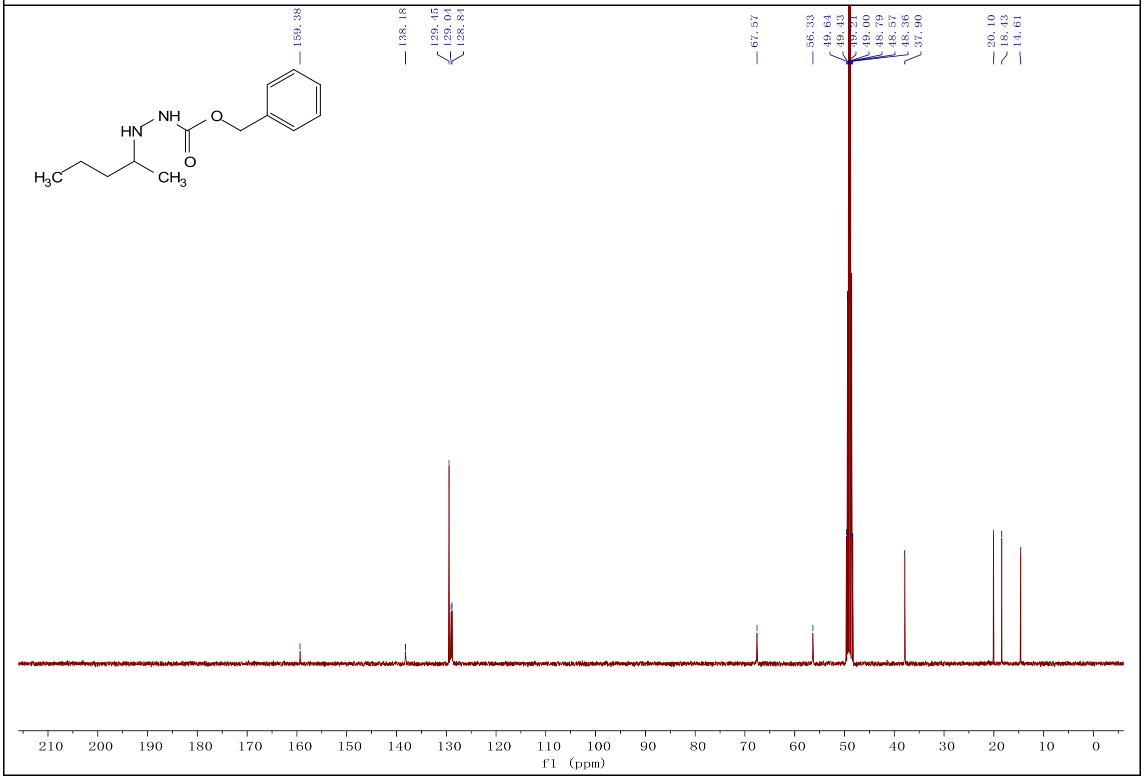

benzyl 2-(3,3-dimethylbutan-2-yl)hydrazine-1-carboxylate (8a)

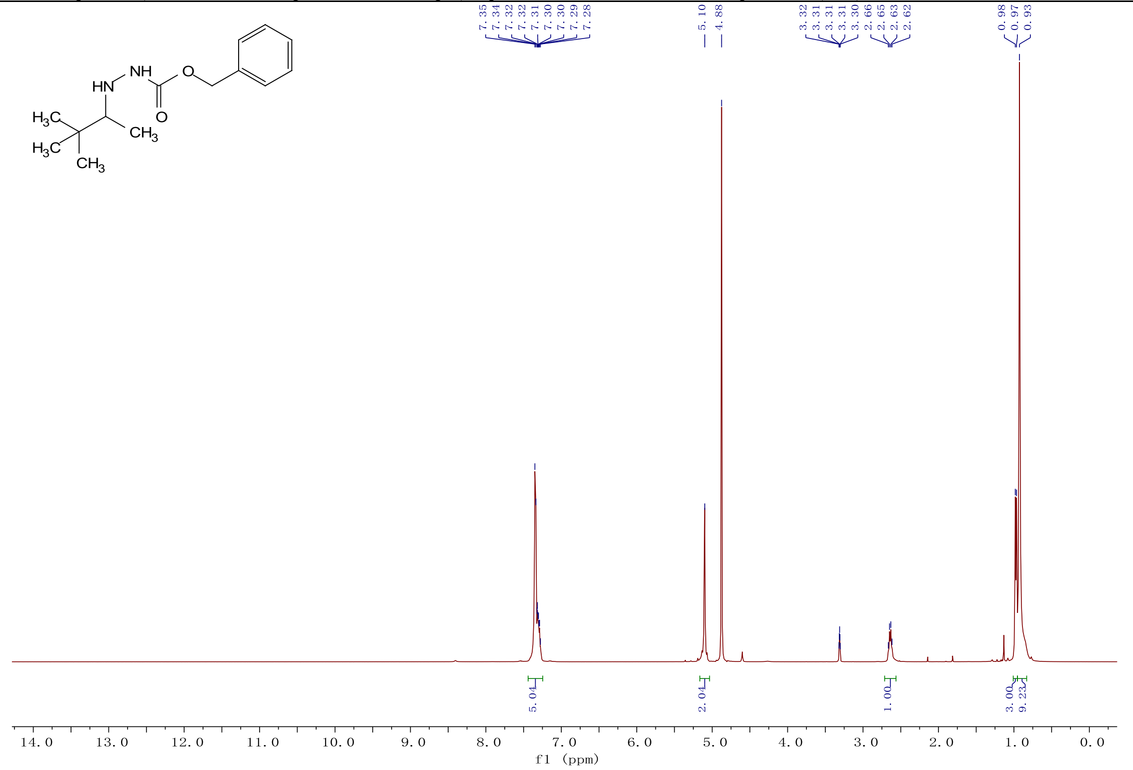

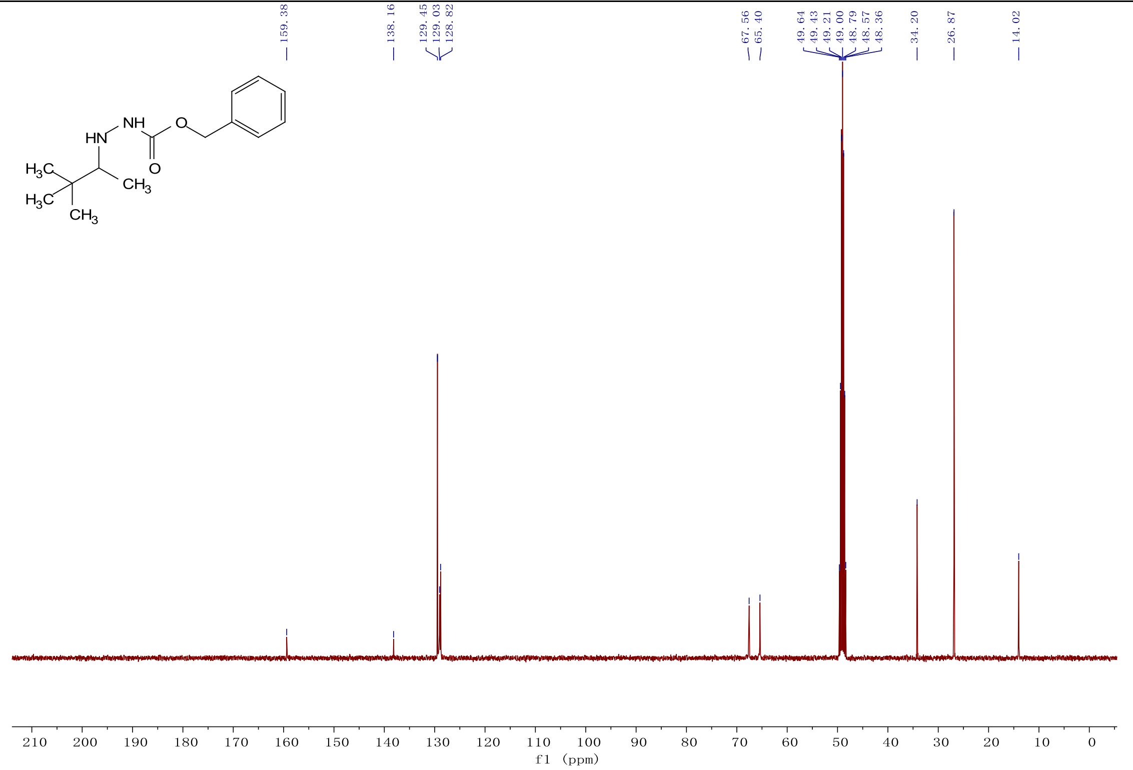

N'-(1-phenylethyl)benzohydrazide (9a)

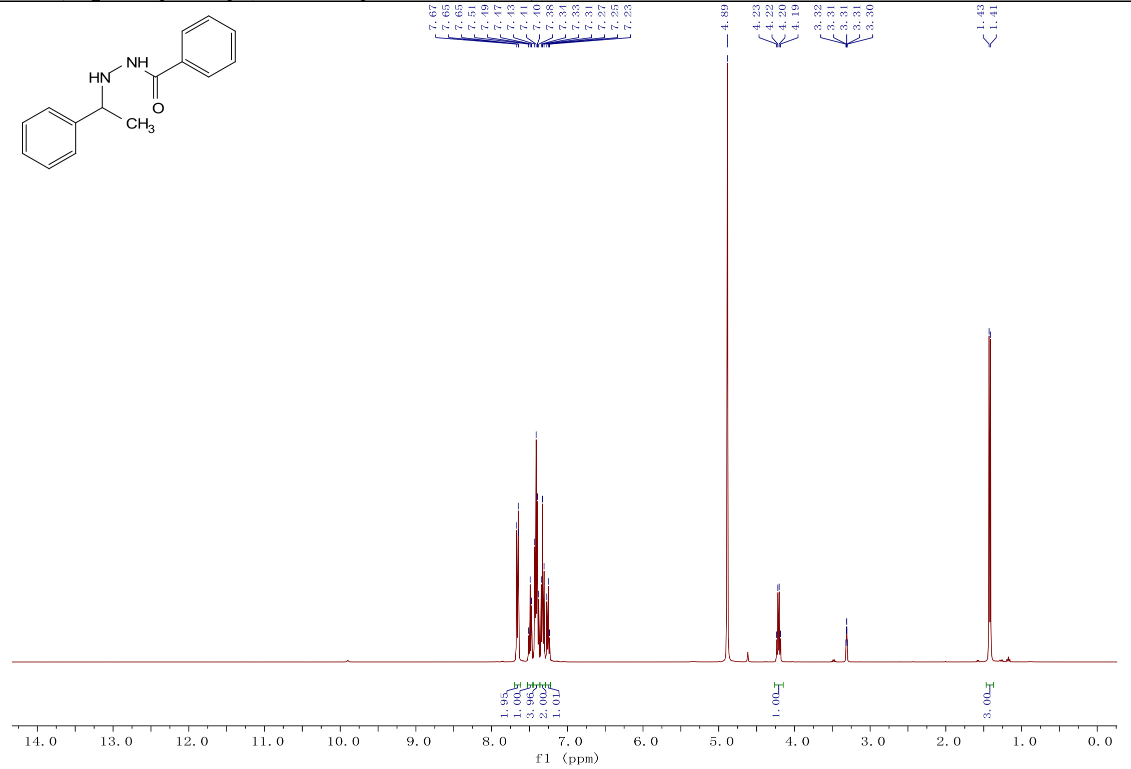

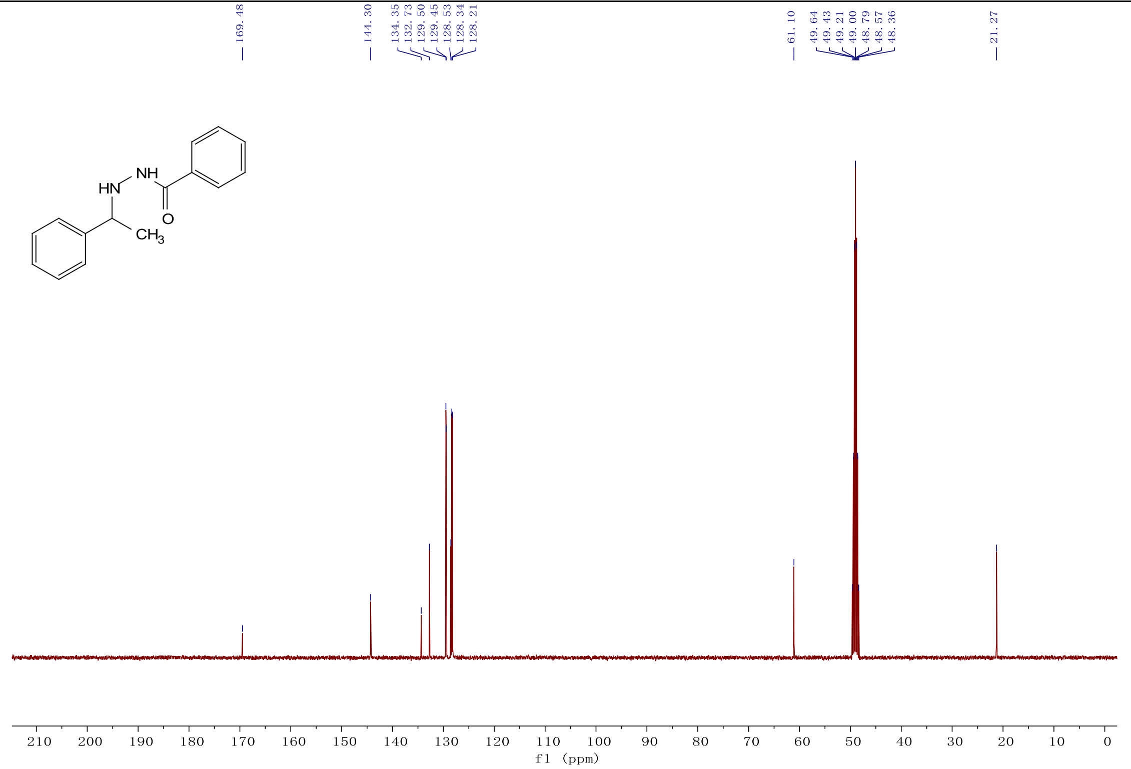

***N'*-(pentan-2-yl)benzohydrazide (10a)**

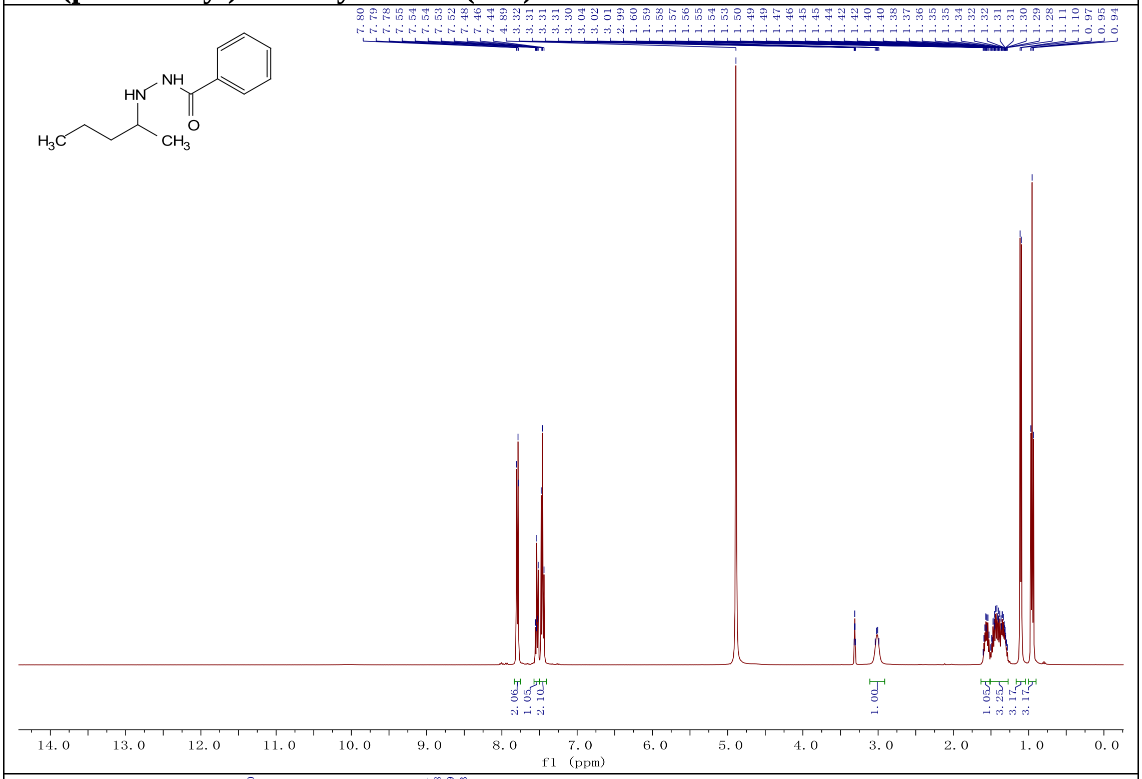

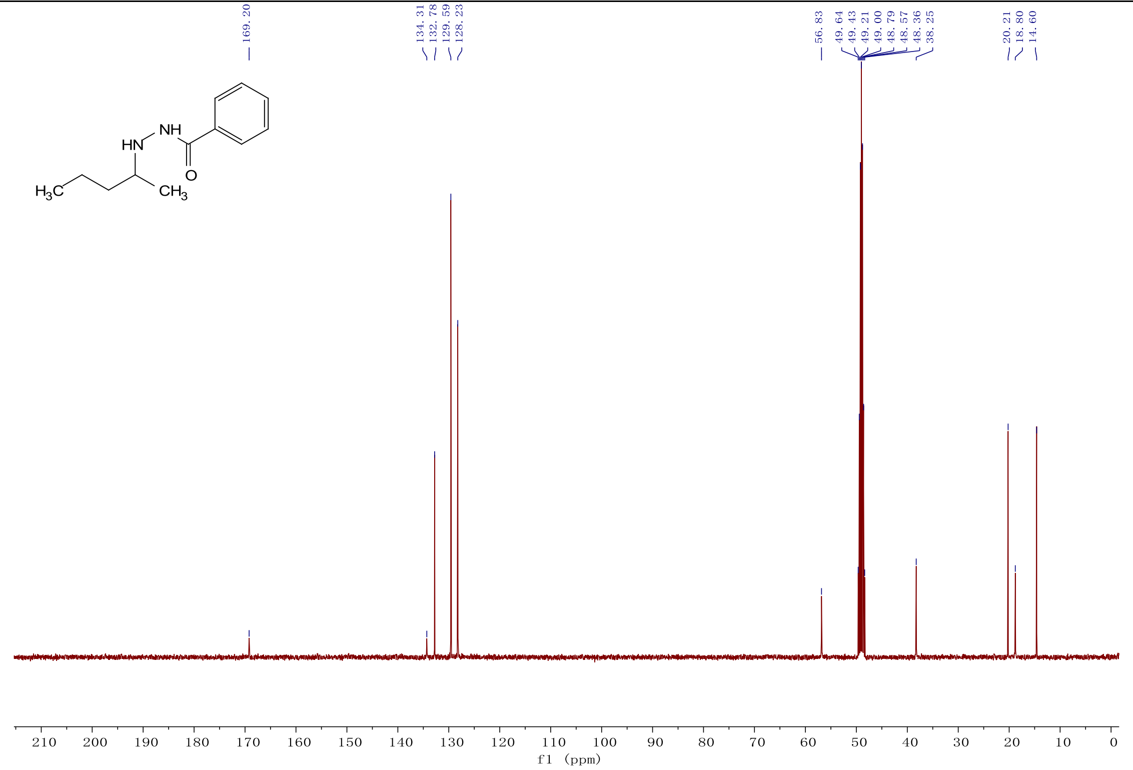

***tert*-butyl 2-(3,3-dimethylbutan-2-yl)hydrazine-1-carboxylate (11a)**

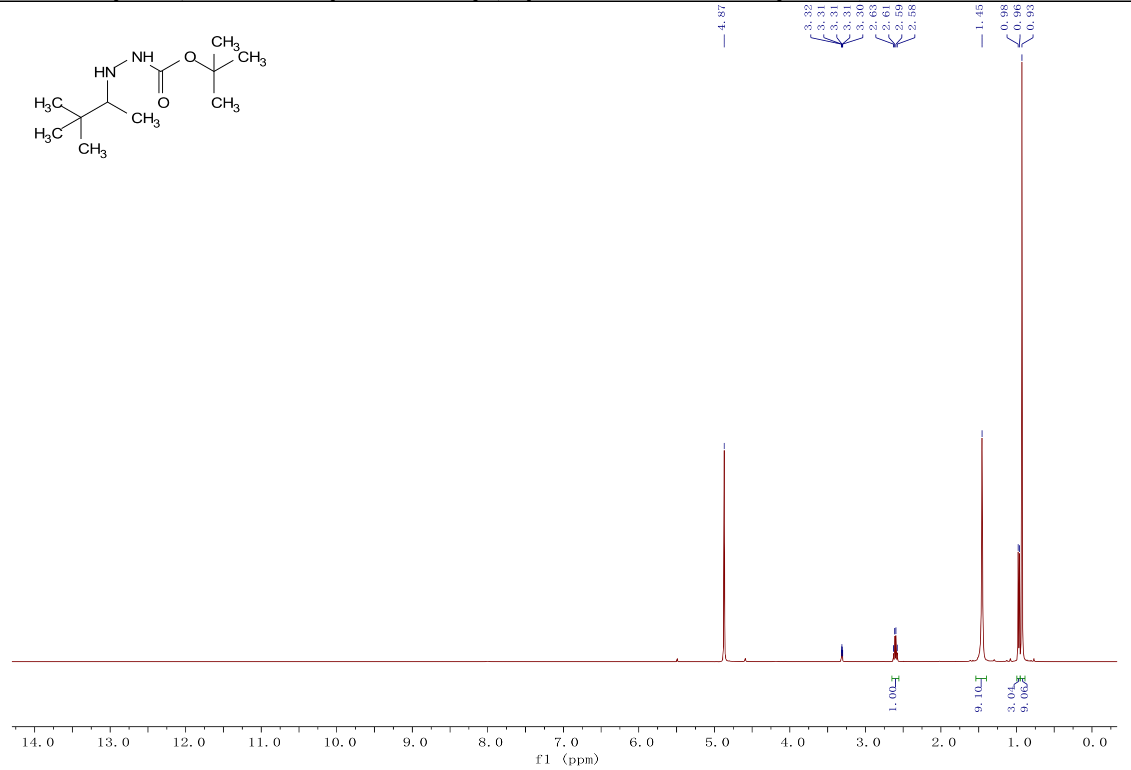

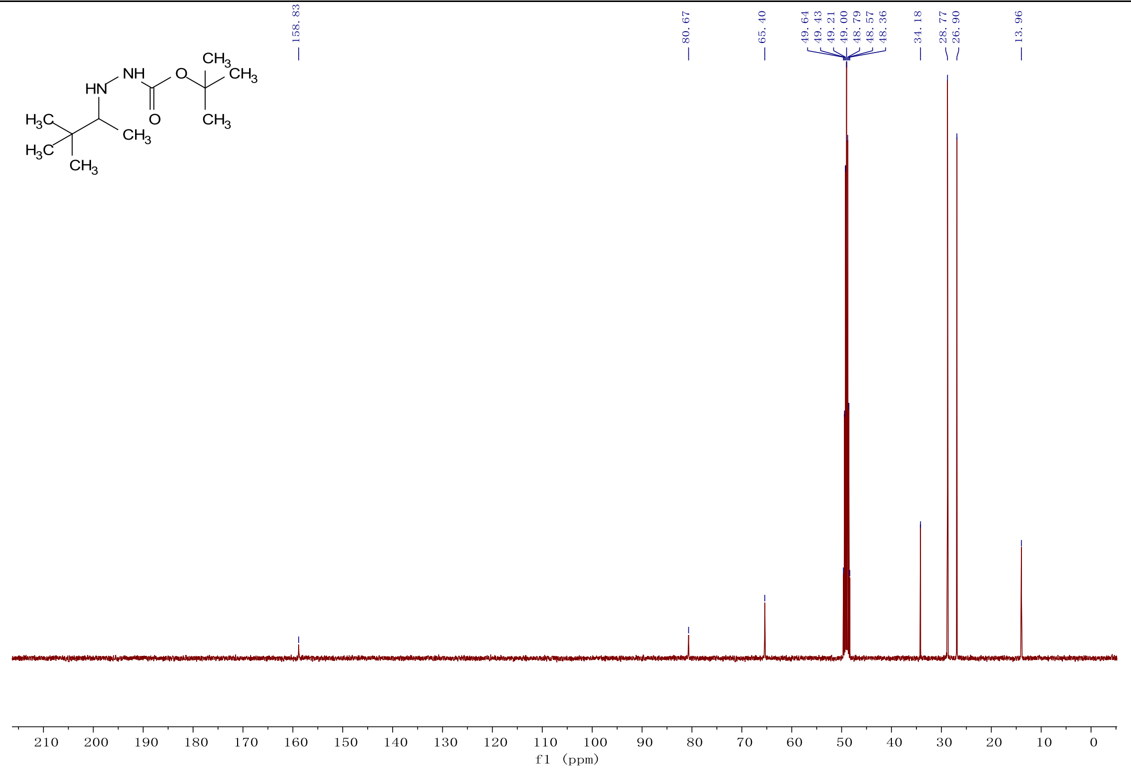

Chiral Chromatograms

Product **1a**

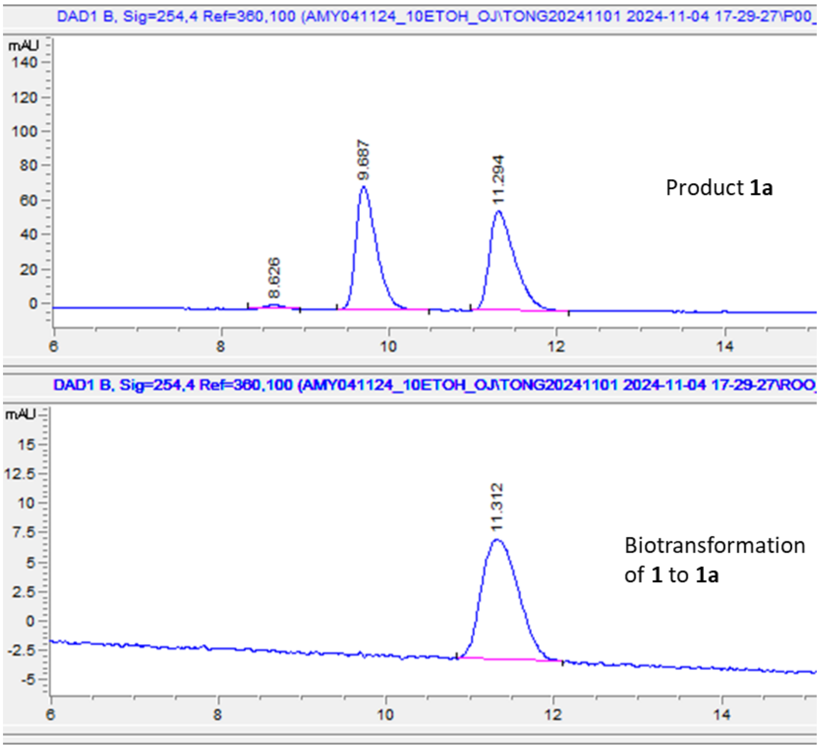

1a: Diacel CHIRALCEL OJ-H column (particle size 5 μm, dimensions: 4.6 x 250 mm) over 20 minutes with an isocratic method (20:80 EtOH: Hexane 0.1% DEA) at 1 mL/min. Enantiomers eluted at 9.687 and 11.294 minutes.

Product **2a**

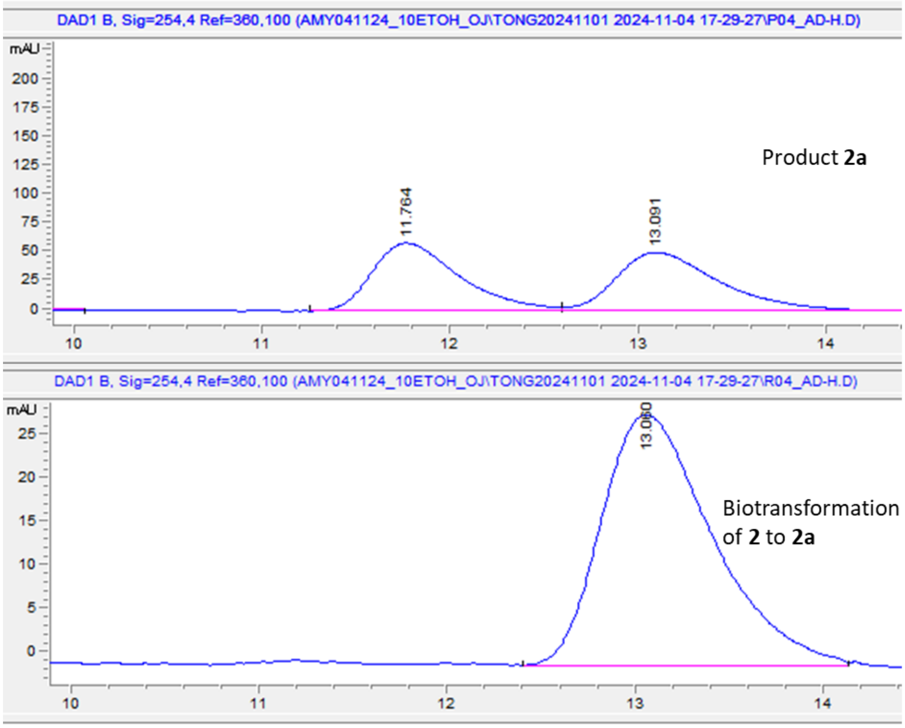

2a: Diacel CHIRALPAK® AD-H column (particle size 5 μm, dimensions: 4.6 x 250 mm) over 30 minutes with an isocratic method (20:80 EtOH: Hexane 0.1% DEA) at 1 mL/min. Enantiomers eluted at 11.764 and 13.091 minutes.

Product **3a**

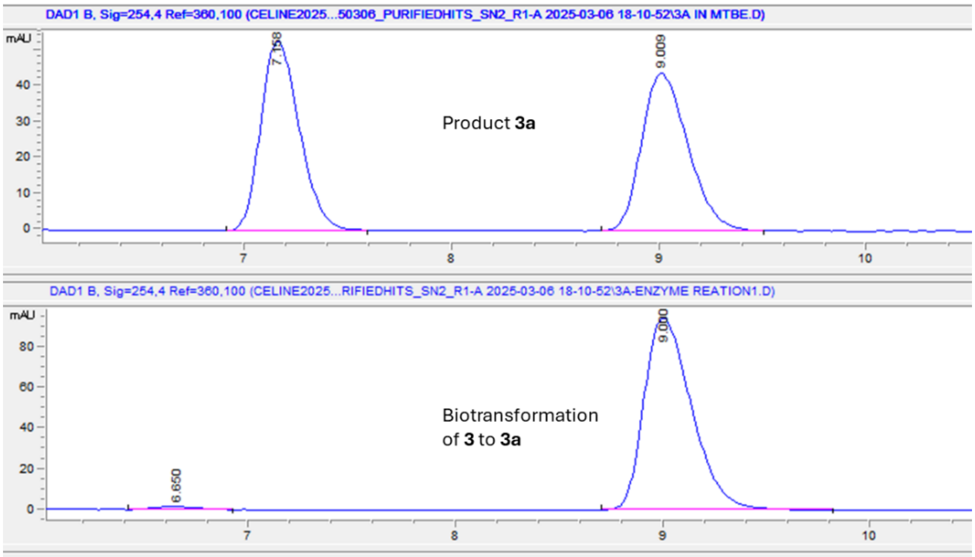

3a: Diacel CHIRALPAK® IE column (particle size 5 µm, dimensions: 4.6 x 250 mm) over 30 minutes with an isocratic method (20:80 EtOH: Hexane) at 1 mL/min. Enantiomers eluted at 7.158 and 9.009 minutes.

Product **4a**

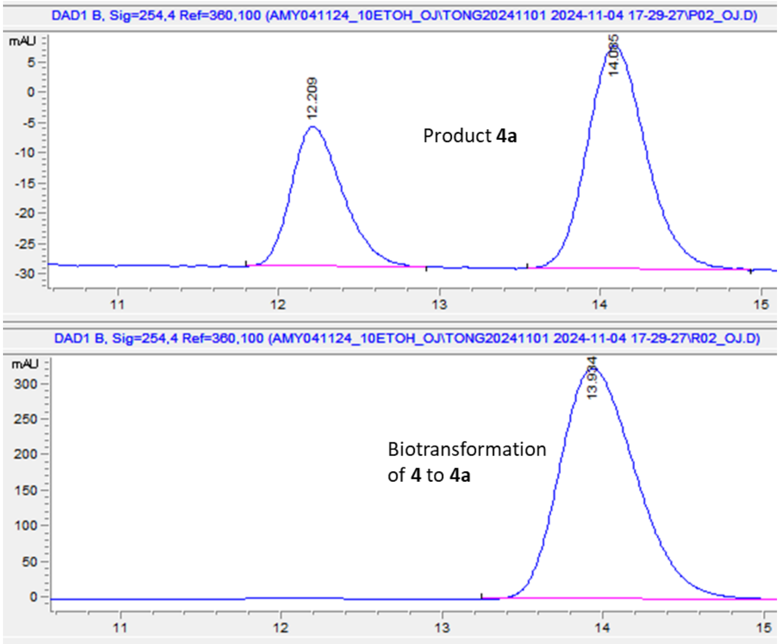

4a: Diacel CHIRALCEL OJ-H column (particle size 5 μm, dimensions: 4.6 x 250 mm) over 20 minutes with an isocratic method (20:80 EtOH: Hexane 0.1% DEA) at 1 mL/min. Enantiomers eluted at 12.209 and 14.035 minutes.

Product **5a**

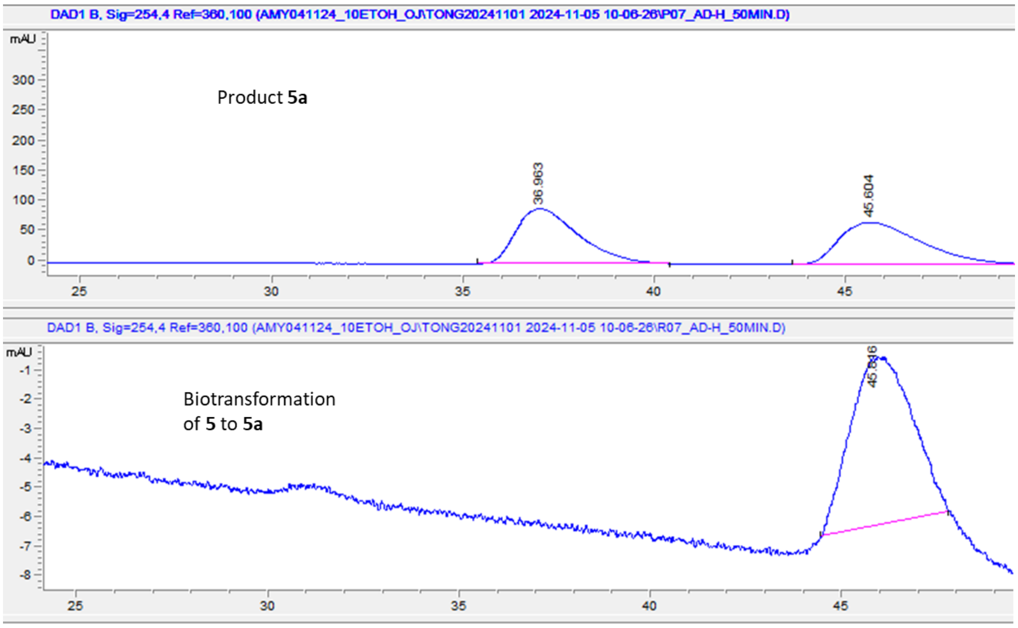

5a: Diacel CHIRALPAK® AD-H column (particle size 5 µm, dimensions: 4.6 x 250 mm) over 50 minutes with an isocratic method (20:80 EtOH: Hexane 0.1% DEA) at 1 mL/min. Enantiomers eluted at 36.963 and 45.604 minutes.

Product **6a**

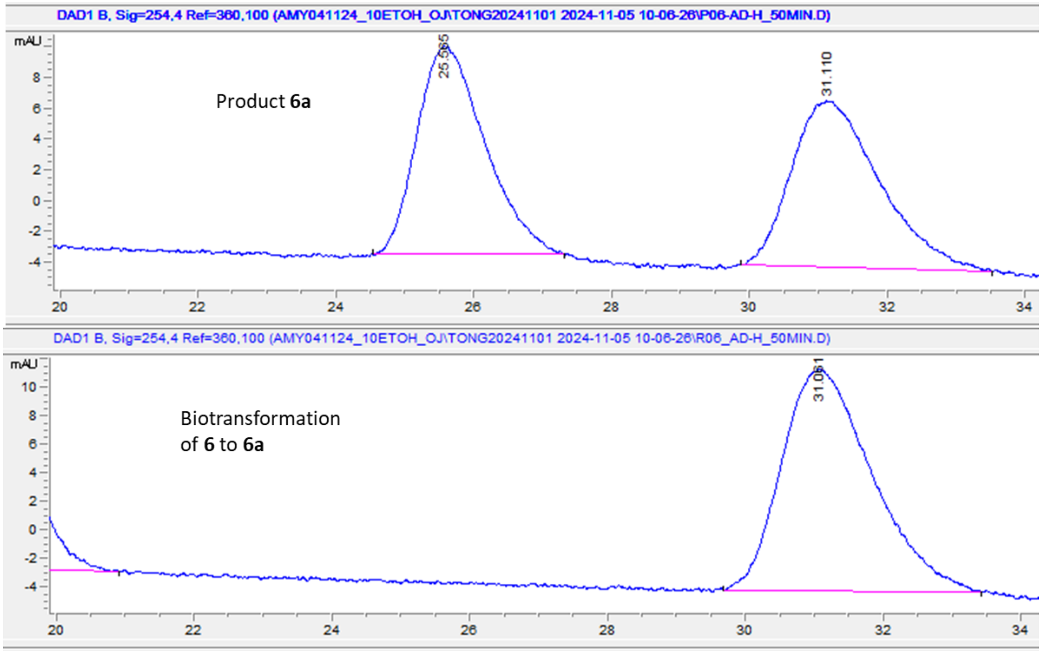

6a: Diacel CHIRALPAK® AD-H column (particle size 5 µm, dimensions: 4.6 x 250 mm) over 50 minutes with an isocratic method (20:80 EtOH: Hexane 0.1% DEA) at 1 mL/min. Enantiomers eluted at 25.585 and 31.110 minutes.

Product **7a**

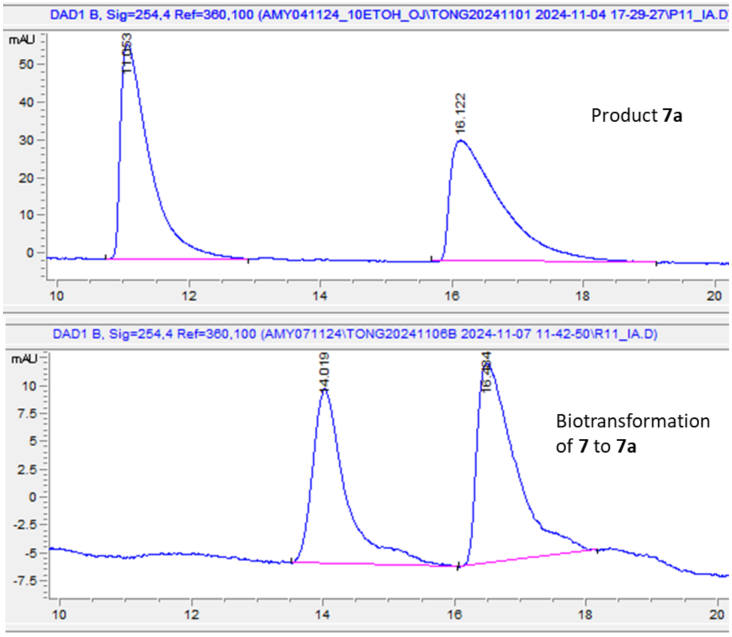

7a: Diacel CHIRALPAK® IA column (particle size 5 µm, dimensions: 4.6 x 250 mm) over 30 minutes with an isocratic method (20:80 EtOH: Hexane 0.1% DEA) at 1 mL/min. Enantiomers eluted at 11.053 and 16.122 minutes.

Product **8a**

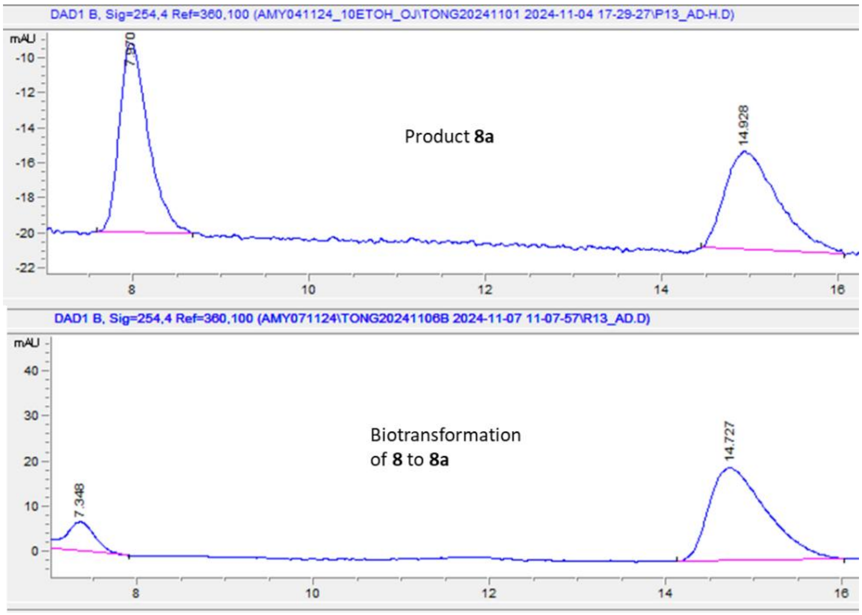

8a: Diacel CHIRALPAK® AD-H column (particle size 5 μm, dimensions: 4.6 x 250 mm) over 30 minutes with an isocratic method (20:80 EtOH: Hexane 0.1% DEA) at 1 mL/min. Enantiomers eluted at 7.970 and 14.928 minutes.

Product **9a**

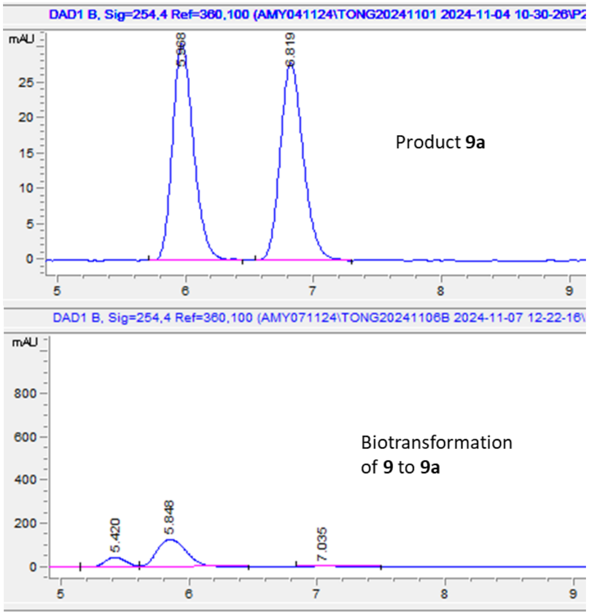

9a: Diacel CHIRALCEL OJ-H column (particle size 5 µm, dimensions: 4.6 x 250 mm) over 20 minutes with an isocratic method (20:80 EtOH: Hexane 0.1% DEA) at 1 mL/min. Enantiomers eluted at 5.968 and 6.819 minutes.

Product **10a**

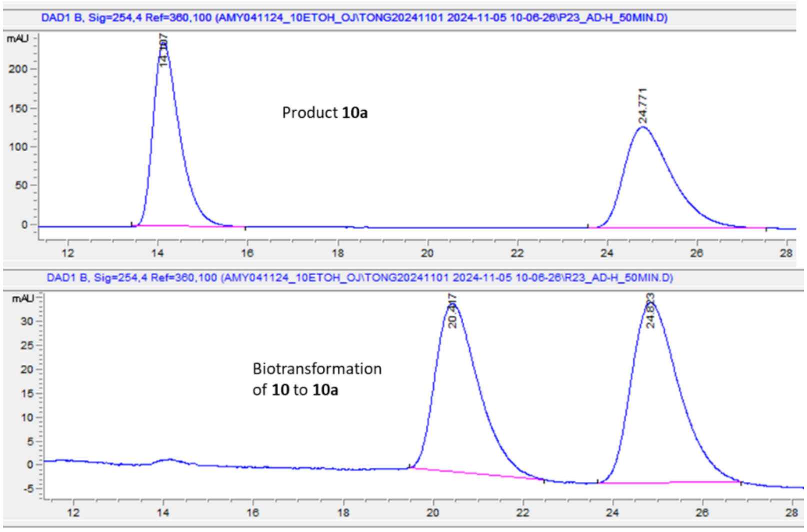

10a: Diacel CHIRALPAK® AD-H column (particle size 5 µm, dimensions: 4.6 x 250 mm) over 50 minutes with an isocratic method (20:80 EtOH: Hexane 0.1% DEA) at 1 mL/min. Enantiomers eluted at 14.107 and24.771 minutes.

Product **11a**

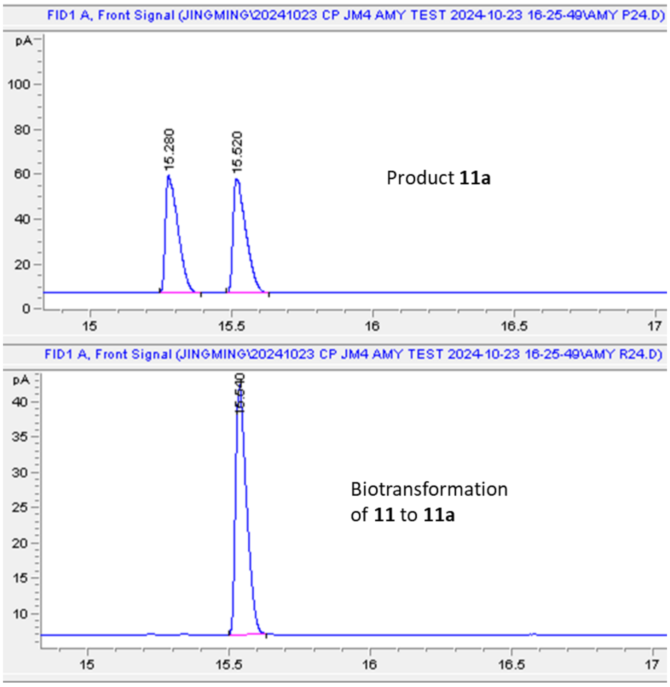

11a: Agilent 7890A GC system, an FID detector, and an Agilent J&W GC column (CP-Chirasil-Dex CB, 25 m x 0.25 mm, 0.25 µm film). A 1 µL sample was injected with a detector temperature 200 °C. The temperature gradient started from 80 °C, then increased to 200 °C (5 °C per min) and held for 2 min. The total run time was 30 min. Enantiomers eluted at 15.280 and 15.520 minutes.

References

[1] A. J. Burke, T. M. Lister, J. R. Marshall, M. J. B. Brown, R. Lloyd, A. P. Green, N. J. Turner, *ChemCatChem* **2023**, *15*, DOI 10.1002/cctc.202300256.

[2] P. Yang, C. Zhang, Y. Ma, C. Zhang, A. Li, B. Tang, J. S. Zhou, *Angew. Chem. Int. Ed.* **2017**, *56*, 14702 –14706.

[3] K. E. Atkin, R. Reiss, N. J. Turner, A. M. Brzozowski, G. Grogan, *Acta Crystallogr. Sect. F. Struct. Biol. Cryst. Commun.* **2008**, *64*, 182–185.

[4] W. Kabsch, *Acta Crystallogr. D. Biol. Crystallogr.* **2010**, *66*, 125–132.

[5] P. Evans, in *Acta Crystallogr. D. Biol. Crystallogr.* **2006**, pp. 72–82.

[6] G. Winter, *J. Appl. Crystallogr.* **2010**, *43*, 186–190.

[7] A. Vagin, A. Teplyakov, *J. Appl. Crystallogr.* **1997**, *30*, 1022–1025.

[8] V. Harawa, T. W. Thorpe, J. R. Marshall, J. J. Sangster, A. K. Gilio, L. Pirvu, R. S. Heath, A. Angelastro, J. D. Finnigan, S. J. Charnock, J. W. Nafie, G. Grogan, R. C. Whitehead, N. J. Turner, *J. Am. Chem. Soc.* **2022**, *144*, 21088–21095.

[9] P. Emsley, K. Cowtan, *Acta Crystallogr. D. Biol. Crystallogr.* **2004**, *60*, 2126–2132.

[10] G. N. Murshudov Alexe, A. Vagin, E. J. Dodson, *Acta Crystallogr.Sect. D. Biol*, **1997**, *53*, 240-255.

[11] J. Eberhardt, D. Santos-Martins, A. F. Tillack, S. Forli, *J. Chem. Inf. Model* **2021**, *61*, 3891–3898.

[12] F. Long, R. A. Nicholls, P. Emsley, S. Gražulis, A. Merkys, A. Vaitkus, G. N. Murshudov, *Acta Crystallogr. D. Struct. Biol.* **2017**, *73*, 112–122.
